# Supplementary material for: Vitamin B12 Status before and after Outpatient Treatment of Severe Acute Malnutrition in Children Aged 6–59 Months: A Sub-Study of a Randomized Controlled Trial in Burkina Faso
Source: Nutrients. 2023 Aug 8;15(16):3496. doi: 10.3390/nu15163496 (PMC10458940; doi:10.3390/nu15163496)
Supplement: Supplementary file 1 [file nutrients-15-03496-s001.zip › nutrients-2449093-supplementary.pdf]

id	study	dosage	sex	age_adm	age_exit	age_cat_a	age_cat_e	breastf_ad
1	No	Reduced	Male	10.11827	10.80815	< 12mo	< 12mo	Yes
2	No	Reduced	Female	16	18	>=12mo	>=12mo	Yes
3	No	Standard	Female	6.406045	10.08541	< 12mo	< 12mo	Yes
4	Yes	Reduced	Male	14.159	17.83837	>=12mo	>=12mo	Yes
5	No	Standard	Male	9.36268	13.04205	< 12mo	>=12mo	Yes
6	No	Standard	Male	6.471748	9.461235	< 12mo	< 12mo	Yes
7	No	Reduced	Female	13.10775	14.71748	>=12mo	>=12mo	Yes
8	No	Standard	Female	13.30486	16.98423	>=12mo	>=12mo	Yes
9	No	Standard	Male	14	14	>=12mo	>=12mo	Yes
10	No	Reduced	Female	6.208936	9.888305	< 12mo	< 12mo	Yes
11	No	Reduced	Female	24	26	>=12mo	>=12mo	No
12	No	Reduced	Male	24	28	>=12mo	>=12mo	Yes
13	Yes	Standard	Male	6	10	< 12mo	< 12mo	Yes
14	No	Reduced	Female	23.02891	23.94875	>=12mo	>=12mo	No
15	No	Standard	Female	29	31	>=12mo	>=12mo	No
16	No	Standard	Female	22.79895	23.71879	>=12mo	>=12mo	No
17	No	Reduced	Male	36	38	>=12mo	>=12mo	No
18	No	Reduced	Female	8.935611	10.54534	< 12mo	< 12mo	Yes
19	No	Standard	Female	25.49277	27.56242	>=12mo	>=12mo	Yes
20	No	Standard	Female	19	20	>=12mo	>=12mo	No
21	No	Standard	Male	18.5611	19.94087	>=12mo	>=12mo	No
22	Yes	Reduced	Male	8	9	< 12mo	< 12mo	Yes
23	Yes	Standard	Male	21.71485	23.32457	>=12mo	>=12mo	Yes
24	No	Reduced	Male	6.208936	9.658344	< 12mo	< 12mo	Yes
25	No	Standard	Female	12.54928	13.46912	>=12mo	>=12mo	Yes
26	Yes	Standard	Male	10.47963	12.54928	< 12mo	>=12mo	Yes
27	Yes	Reduced	Male	25.0657	28.28515	>=12mo	>=12mo	Yes
28	Yes	Standard	Male	33.6728	34.59264	>=12mo	>=12mo	No
29	Yes	Reduced	Female	28.84363	30.45335	>=12mo	>=12mo	No
30	Yes	Reduced	Female	6.636005	10.31537	< 12mo	< 12mo	Yes
31	Yes	Reduced	Male	13.37057	14.29041	>=12mo	>=12mo	Yes
32	No	Standard	Male	6.931669	10.61104	< 12mo	< 12mo	Yes
33	Yes	Standard	Female	20.69645	22.53614	>=12mo	>=12mo	No
34	Yes	Reduced	Female	34.42838	36.72799	>=12mo	>=12mo	No
35	Yes	Reduced	Male	9.264126	11.56373	< 12mo	< 12mo	Yes
36	Yes	Standard	Female	22.07622	22.7661	>=12mo	>=12mo	Yes
37	Yes	Standard	Female	16.2615	17.87122	>=12mo	>=12mo	Yes
38	Yes	Standard	Female	18.75821	21.51774	>=12mo	>=12mo	Yes
39	Yes	Reduced	Male	13.1406	15.21025	>=12mo	>=12mo	Yes
40	Yes	Reduced	Male	11.49803	15.1774	< 12mo	>=12mo	Yes
41	Yes	Reduced	Male	16.62286	17.31275	>=12mo	>=12mo	No
42	Yes	Standard	Female	17.87122	19.02103	>=12mo	>=12mo	Yes
43	No	Standard	Female	6	10	< 12mo	< 12mo	Yes
44	No	Standard	Female	19.31669	21.6163	>=12mo	>=12mo	No
45	No	Standard	Female	6.767411	8.837056	< 12mo	< 12mo	Yes
46	Yes	Reduced	Female	17	17	>=12mo	>=12mo	No
47	Yes	Standard	Female	11.53088	15.21025	< 12mo	>=12mo	Yes
48	Yes	Reduced	Male	18.39685	20.46649	>=12mo	>=12mo	Yes
49	Yes	Standard	Male	33	37	>=12mo	>=12mo	No
50	Yes	Standard	Male	40.70302	41.62286	>=12mo	>=12mo	No

51	Yes	Reduced	Male	19.21813	20.36794	>=12mo	>=12mo	Yes
52	Yes	Reduced	Male	13.17346	14.55322	>=12mo	>=12mo	Yes
53	Yes	Reduced	Male	24.34297	25.49277	>=12mo	>=12mo	Yes
54	No	Reduced	Female	7.654402	9.494086	< 12mo	< 12mo	Yes
55	Yes	Standard	Male	6.73456	9.264126	< 12mo	< 12mo	Yes
56	Yes	Standard	Female	16.95138	20.63075	>=12mo	>=12mo	Yes
57	No	Reduced	Male	6.340342	7.720105	< 12mo	< 12mo	Yes
58	Yes	Standard	Female	58	62	>=12mo	>=12mo	No
59	No	Reduced	Male	8.311432	8.541393	< 12mo	< 12mo	Yes
60	No	Standard	Female	16.78712	19.77661	>=12mo	>=12mo	Yes
61	Yes	Reduced	Male	9.691196	13.37057	< 12mo	>=12mo	Yes
62	Yes	Standard	Male	6.208936	9.888305	< 12mo	< 12mo	Yes
63	No	Reduced	Female	16.06439	19.74376	>=12mo	>=12mo	Yes
64	Yes	Reduced	Male	24.57293	25.03285	>=12mo	>=12mo	No
65	No	Standard	Female	11	15	< 12mo	>=12mo	Yes
66	No	Standard	Female	29	32	>=12mo	>=12mo	No
67	Yes	Standard	Female	9	10	< 12mo	< 12mo	Yes
68	No	Reduced	Female	6.143233	9.822602	< 12mo	< 12mo	Yes
69	Yes	Reduced	Female	9.13272	10.05256	< 12mo	< 12mo	Yes
70	Yes	Standard	Male	18.69251	19.61235	>=12mo	>=12mo	Yes
71	Yes	Reduced	Female	9.067018	12.0565	< 12mo	>=12mo	Yes
72	Yes	Standard	Female	15.99869	17.83837	>=12mo	>=12mo	Yes
73	Yes	Reduced	Female	9.36268	10.9724	< 12mo	< 12mo	Yes
74	Yes	Standard	Male	17.14849	17.83837	>=12mo	>=12mo	Yes
75	Yes	Reduced	Male	9.494086	13.17346	< 12mo	>=12mo	Yes
76	No	Reduced	Female	6.471748	9.921156	< 12mo	< 12mo	Yes
77	Yes	Reduced	Female	11.20237	14.88174	< 12mo	>=12mo	Yes
78	No	Reduced	Male	22.30618	25.0657	>=12mo	>=12mo	Yes
79	Yes	Standard	Female	10.18397	11.79369	< 12mo	< 12mo	Yes
80	Yes	Standard	Female	21.12352	22.50328	>=12mo	>=12mo	Yes
81	No	Standard	Male	18.62681	19.31669	>=12mo	>=12mo	Yes
82	No	Standard	Male	7.358738	11.03811	< 12mo	< 12mo	Yes
83	No	Reduced	Male	8.04862	9.428384	< 12mo	< 12mo	Yes
84	No	Reduced	Female	6.471748	9.231275	< 12mo	< 12mo	Yes
85	No	Reduced	Female	6.931669	10.15112	< 12mo	< 12mo	Yes
86	No	Reduced	Female	14.55322	18.23259	>=12mo	>=12mo	Yes
87	No	Standard	Male	6	10	< 12mo	< 12mo	Yes
88	No	Standard	Male	11.9251	14.45467	< 12mo	>=12mo	Yes
89	No	Standard	Male	6.340342	8.180026	< 12mo	< 12mo	Yes
90	No	Reduced	Female	7.194481	10.87385	< 12mo	< 12mo	Yes
91	No	Standard	Female	6.997372	8.147175	< 12mo	< 12mo	Yes
92	Yes	Reduced	Male	12.02365	12.9435	>=12mo	>=12mo	Yes
93	Yes	Standard	Male	12.87779	14.0276	>=12mo	>=12mo	Yes
94	No	Standard	Male	25.42707	25.42707	>=12mo	>=12mo	No
95	Yes	Reduced	Male	8.344284	9.954008	< 12mo	< 12mo	Yes
96	No	Standard	Female	9.165571	10.08541	< 12mo	< 12mo	Yes
97	No	Reduced	Male	21.18922	24.86859	>=12mo	>=12mo	Yes
98	Yes	Reduced	Male	11.66229	13.96189	< 12mo	>=12mo	Yes
99	No	Reduced	Female	37.38502	40.14455	>=12mo	>=12mo	No
100	No	Standard	Female	7.490145	10.47963	< 12mo	< 12mo	Yes
101	Yes	Standard	Female	6.570302	9.099869	< 12mo	< 12mo	Yes

102	No	Reduced	Male	6.30749	8.147175	< 12mo	< 12mo	Yes
103	Yes	Reduced	Female	22	23	>=12mo	>=12mo	Yes
104	Yes	Standard	Male	7.950066	9.099869	< 12mo	< 12mo	Yes
105	No	Reduced	Male	9.13272	10.05256	< 12mo	< 12mo	Yes
106	Yes	Standard	Female	9.921156	10.841	< 12mo	< 12mo	Yes
107	No	Reduced	Female	6	9	< 12mo	< 12mo	Yes
108	Yes	Standard	Male	6.143233	9.822602	< 12mo	< 12mo	Yes
109	Yes	Standard	Male	6.208936	9.198423	< 12mo	< 12mo	Yes
110	Yes	Reduced	Female	8.541393	11.07096	< 12mo	< 12mo	Yes
111	Yes	Standard	Female	8.607096	12.0565	< 12mo	>=12mo	Yes
112	No	Reduced	Female	6.110381	8.639948	< 12mo	< 12mo	Yes
113	Yes	Standard	Female	6.406045	10.08541	< 12mo	< 12mo	Yes
114	No	Standard	Female	6.241787	7.621551	< 12mo	< 12mo	Yes
115	Yes	Reduced	Female	6.208936	9.888305	< 12mo	< 12mo	Yes
116	Yes	Standard	Male	6.274639	9.954008	< 12mo	< 12mo	Yes
117	No	Reduced	Female	6.537451	10.21682	< 12mo	< 12mo	Yes
118	No	Standard	Female	6.143233	9.822602	< 12mo	< 12mo	Yes
119	Yes	Reduced	Female	6.208936	9.888305	< 12mo	< 12mo	Yes
120	Yes	Reduced	Female	6.504599	10.18397	< 12mo	< 12mo	Yes
121	No	Reduced	Female	6.208936	9.888305	< 12mo	< 12mo	Yes
122	Yes	Reduced	Male	6.110381	7.030223	< 12mo	< 12mo	Yes
123	Yes	Standard	Male	7.39159	8.541393	< 12mo	< 12mo	Yes
124	Yes	Standard	Male	6.274639	9.954008	< 12mo	< 12mo	Yes
125	No	Standard	Male	6.011827	7.851511	< 12mo	< 12mo	Yes
126	No	Standard	Female	6	8	< 12mo	< 12mo	Yes
127	No	Reduced	Female	6.208936	9.658344	< 12mo	< 12mo	Yes
128	Yes	Reduced	Male	6.438896	9.428384	< 12mo	< 12mo	Yes
129	No	Standard	Male	16.06439	16.98423	>=12mo	>=12mo	Yes
130	Yes	Reduced	Female	8.607096	10.44678	< 12mo	< 12mo	Yes
131	Yes	Standard	Male	23.12746	26.11695	>=12mo	>=12mo	Yes
132	No	Reduced	Female	9.55979	12.54928	< 12mo	>=12mo	Yes
133	No	Standard	Male	6.997372	10.67674	< 12mo	< 12mo	Yes
134	Yes	Reduced	Male	20.5979	23.12746	>=12mo	>=12mo	Yes
135	No	Reduced	Male	9.13272	10.05256	< 12mo	< 12mo	Yes
136	Yes	Standard	Male	13.79764	16.09724	>=12mo	>=12mo	Yes
137	Yes	Standard	Male	20.46649	22.7661	>=12mo	>=12mo	Yes
138	Yes	Reduced	Female	6.636005	8.245729	< 12mo	< 12mo	Yes
139	No	Standard	Male	7.687254	11.36662	< 12mo	< 12mo	Yes
140	No	Standard	Male	14.81603	15.50591	>=12mo	>=12mo	Yes
141	No	Reduced	Male	8.508541	10.34823	< 12mo	< 12mo	Yes
142	Yes	Reduced	Female	6.274639	9.954008	< 12mo	< 12mo	Yes
143	Yes	Standard	Male	9.986859	11.82654	< 12mo	< 12mo	Yes
144	Yes	Standard	Female	12	16	>=12mo	>=12mo	Yes
145	No	Reduced	Female	7.095926	10.08541	< 12mo	< 12mo	Yes
146	No	Reduced	Female	6.241787	9.691196	< 12mo	< 12mo	Yes
147	Yes	Standard	Female	6.373193	7.293036	< 12mo	< 12mo	Yes
148	Yes	Reduced	Female	16.29435	16.75427	>=12mo	>=12mo	Yes
149	No	Reduced	Female	6.208936	9.198423	< 12mo	< 12mo	Yes
150	No	Reduced	Male	7.161629	9.001314	< 12mo	< 12mo	Yes
151	No	Reduced	Female	6.110381	9.78975	< 12mo	< 12mo	Yes
152	No	Standard	Male	12.08936	12.77924	>=12mo	>=12mo	Yes

153	No	Standard	Female	7.095926	8.70565	< 12mo	< 12mo	Yes
154	No	Reduced	Male	6.077529	9.756899	< 12mo	< 12mo	Yes
155	No	Standard	Female	17.57556	18.95532	>=12mo	>=12mo	No
156	No	Standard	Male	6.340342	10.01971	< 12mo	< 12mo	Yes
157	No	Reduced	Female	7.81866	11.49803	< 12mo	< 12mo	Yes
158	No	Reduced	Male	12.87779	14.25756	>=12mo	>=12mo	Yes
159	No	Reduced	Male	11.95795	13.33771	< 12mo	>=12mo	Yes
160	No	Standard	Male	15.80158	17.64126	>=12mo	>=12mo	Yes
161	No	Standard	Male	15.93298	16.62286	>=12mo	>=12mo	Yes
162	No	Standard	Female	6.044678	7.884363	< 12mo	< 12mo	Yes
163	No	Standard	Male	25	25	>=12mo	>=12mo	No
164	No	Reduced	Female	10.9067	13.89619	< 12mo	>=12mo	Yes
165	No	Standard	Male	6.931669	8.081471	< 12mo	< 12mo	Yes
166	No	Reduced	Female	16	18	>=12mo	>=12mo	Yes
167	No	Standard	Female	8.70565	10.54534	< 12mo	< 12mo	Yes
168	No	Reduced	Male	11.26807	12.87779	< 12mo	>=12mo	Yes
169	No	Standard	Female	6.537451	9.526938	< 12mo	< 12mo	Yes
170	No	Standard	Male	15.1117	16.03154	>=12mo	>=12mo	Yes
171	No	Reduced	Male	12.81209	13.96189	>=12mo	>=12mo	Yes
172	No	Standard	Male	14.58607	18.26544	>=12mo	>=12mo	Yes
173	No	Reduced	Female	6.997372	8.147175	< 12mo	< 12mo	Yes
174	No	Standard	Male	16.42576	17.80552	>=12mo	>=12mo	Yes
175	No	Reduced	Male	6.668857	8.508541	< 12mo	< 12mo	Yes
176	No	Reduced	Female	10.41393	13.86334	< 12mo	>=12mo	Yes
177	No	Reduced	Male	11.79369	15.47306	< 12mo	>=12mo	Yes
178	Yes	Reduced	Female	15.73587	18.4954	>=12mo	>=12mo	Yes
179	No	Standard	Female	6.110381	7.490145	< 12mo	< 12mo	Yes
180	No	Standard	Male	24	25	>=12mo	>=12mo	No
181	No	Reduced	Female	12.54928	14.84888	>=12mo	>=12mo	Yes
182	No	Standard	Male	8.245729	11.9251	< 12mo	< 12mo	Yes
183	No	Standard	Female	6.208936	8.968463	< 12mo	< 12mo	Yes
184	No	Reduced	Female	15	17	>=12mo	>=12mo	Yes
185	Yes	Standard	Male	12.02365	14.78318	>=12mo	>=12mo	Yes
186	No	Standard	Female	17.93693	19.08673	>=12mo	>=12mo	Yes
187	No	Reduced	Female	7.982917	11.66229	< 12mo	< 12mo	No
188	Yes	Reduced	Female	10.9067	14.81603	< 12mo	>=12mo	Yes
189	No	Reduced	Female	10.93955	13.0092	< 12mo	>=12mo	Yes
190	No	Standard	Male	13.30486	15.60447	>=12mo	>=12mo	Yes
191	Yes	Standard	Male	6.701708	8.311432	< 12mo	< 12mo	Yes
192	Yes	Standard	Female	15.30881	16.45861	>=12mo	>=12mo	No
193	No	Reduced	Female	9.198423	12.87779	< 12mo	>=12mo	Yes
194	Yes	Reduced	Female	17.24704	20.46649	>=12mo	>=12mo	Yes
195	Yes	Reduced	Female	11.13666	12.51643	< 12mo	>=12mo	Yes
196	No	Standard	Male	10.41393	13.40342	< 12mo	>=12mo	Yes
197	Yes	Reduced	Female	7.260184	10.01971	< 12mo	< 12mo	Yes
198	No	Reduced	Male	21	23	>=12mo	>=12mo	Yes
199	Yes	Standard	Male	9.494086	13.17346	< 12mo	>=12mo	Yes
200	No	Standard	Female	13.27201	14.19185	>=12mo	>=12mo	Yes
201	No	Reduced	Female	18.4954	19.64521	>=12mo	>=12mo	No
202	Yes	Reduced	Female	9.954008	13.63338	< 12mo	>=12mo	Yes
203	No	Standard	Male	6.077529	8.837056	< 12mo	< 12mo	Yes

204	Yes	Standard	Male	12.68068	16.13009	>=12mo	>=12mo	Yes
205	Yes	Reduced	Male	19	20	>=12mo	>=12mo	No
206	No	Standard	Male	6.241787	9.921156	< 12mo	< 12mo	Yes
207	Yes	Standard	Male	32.49014	33.86991	>=12mo	>=12mo	No
208	Yes	Reduced	Female	9.756899	13.43627	< 12mo	>=12mo	Yes
209	Yes	Standard	Male	10.34823	13.33771	< 12mo	>=12mo	Yes
210	Yes	Standard	Female	6.701708	9.921156	< 12mo	< 12mo	Yes
211	No	Reduced	Male	12.12221	12.81209	>=12mo	>=12mo	Yes
212	No	Reduced	Male	12.02365	14.32326	>=12mo	>=12mo	Yes
213	Yes	Standard	Male	29.43495	33.11432	>=12mo	>=12mo	Yes
214	No	Standard	Female	8.541393	11.76084	< 12mo	< 12mo	Yes
215	No	Reduced	Female	8.081471	11.13666	< 12mo	< 12mo	Yes
216	No	Standard	Male	6.603154	10.05256	< 12mo	< 12mo	Yes
217	No	Reduced	Male	6.537451	6.767411	< 12mo	< 12mo	Yes
218	No	Standard	Female	10.47963	13.23916	< 12mo	>=12mo	Yes
219	No	Reduced	Male	6.865966	8.245729	< 12mo	< 12mo	Yes
220	Yes	Standard	Male	18.4954	19.41524	>=12mo	>=12mo	No
221	No	Reduced	Male	10	12	< 12mo	>=12mo	Yes
222	No	Reduced	Female	36	38	>=12mo	>=12mo	Yes
223	No	Standard	Male	8.771354	10.38108	< 12mo	< 12mo	Yes
224	No	Standard	Male	9.428384	10.57819	< 12mo	< 12mo	Yes
225	No	Reduced	Male	7.227333	8.837056	< 12mo	< 12mo	Yes
226	No	Reduced	Male	6.931669	9.231275	< 12mo	< 12mo	Yes
227	No	Standard	Female	9.165571	10.08541	< 12mo	< 12mo	Yes
228	No	Standard	Male	6	8	< 12mo	< 12mo	Yes
229	No	Reduced	Female	8.245729	11.9251	< 12mo	< 12mo	Yes
230	No	Reduced	Male	19.38239	21.45204	>=12mo	>=12mo	Yes
231	No	Reduced	Female	8.607096	12.28647	< 12mo	>=12mo	Yes
232	No	Standard	Male	10.87385	12.25361	< 12mo	>=12mo	Yes
233	No	Standard	Female	9.099869	11.39947	< 12mo	< 12mo	Yes
234	No	Standard	Male	6.537451	7.687254	< 12mo	< 12mo	Yes
235	No	Standard	Female	7.555848	10.7753	< 12mo	< 12mo	Yes
236	No	Reduced	Female	6	8	< 12mo	< 12mo	No
237	No	Standard	Female	22	25	>=12mo	>=12mo	Yes
238	No	Reduced	Female	34	36	>=12mo	>=12mo	No
239	No	Reduced	Female	6	6	< 12mo	< 12mo	Yes
240	No	Reduced	Male	9.329829	10.70959	< 12mo	< 12mo	Yes
241	No	Reduced	Female	6	9	< 12mo	< 12mo	Yes
242	No	Standard	Male	6.537451	9.296978	< 12mo	< 12mo	Yes
243	No	Standard	Female	6.964521	10.64389	< 12mo	< 12mo	Yes
244	No	Standard	Male	7.095926	8.245729	< 12mo	< 12mo	Yes
245	No	Reduced	Female	7.621551	9.921156	< 12mo	< 12mo	Yes
246	No	Standard	Female	11.95795	13.33771	< 12mo	>=12mo	Yes
247	No	Reduced	Male	7	8	< 12mo	< 12mo	Yes
248	No	Reduced	Female	6.570302	8.869908	< 12mo	< 12mo	Yes
249	No	Standard	Female	6.373193	6.373193	< 12mo	< 12mo	Yes
250	Yes	Reduced	Female	7	9	< 12mo	< 12mo	Yes
251	No	Standard	Female	6.865966	8.245729	< 12mo	< 12mo	Yes
252	Yes	Standard	Female	8.47569	9.395533	< 12mo	< 12mo	Yes
253	No	Standard	Male	13.89619	15.27595	>=12mo	>=12mo	Yes
254	No	Reduced	Female	7.227333	9.986859	< 12mo	< 12mo	Yes

255	No	Standard	Male	7.621551	11.30092	< 12mo	< 12mo	Yes
256	Yes	Reduced	Male	9.001314	11.76084	< 12mo	< 12mo	Yes
257	No	Reduced	Female	10.01971	13.69908	< 12mo	>=12mo	Yes
258	Yes	Reduced	Male	7.884363	11.33377	< 12mo	< 12mo	Yes
259	No	Standard	Male	12.74639	14.78318	>=12mo	>=12mo	Yes
260	No	Reduced	Female	6.997372	8.837056	< 12mo	< 12mo	Yes
261	No	Reduced	Female	6.077529	6.767411	< 12mo	< 12mo	Yes
262	Yes	Standard	Male	12.45072	13.1406	>=12mo	>=12mo	Yes
263	Yes	Standard	Male	17.14849	19.21813	>=12mo	>=12mo	Yes
264	No	Standard	Female	8.541393	9.921156	< 12mo	< 12mo	Yes
265	Yes	Reduced	Female	7.720105	11.39947	< 12mo	< 12mo	Yes
266	Yes	Reduced	Female	11.8594	13.0092	< 12mo	>=12mo	Yes
267	Yes	Reduced	Female	6.898818	10.57819	< 12mo	< 12mo	Yes
268	Yes	Standard	Male	7.950066	9.329829	< 12mo	< 12mo	Yes
269	Yes	Standard	Female	17	18	>=12mo	>=12mo	No
270	No	Reduced	Female	6.73456	8.574245	< 12mo	< 12mo	Yes
271	Yes	Standard	Female	9.329829	11.62944	< 12mo	< 12mo	Yes
272	Yes	Standard	Female	19.61235	23.29172	>=12mo	>=12mo	Yes
273	Yes	Reduced	Female	6.997372	7.687254	< 12mo	< 12mo	Yes
274	Yes	Reduced	Female	8.968463	10.80815	< 12mo	< 12mo	Yes
275	Yes	Reduced	Male	17	19	>=12mo	>=12mo	No
276	No	Standard	Female	25.52562	27.36531	>=12mo	>=12mo	Yes
277	Yes	Standard	Female	33.34428	33.80421	>=12mo	>=12mo	No
278	Yes	Standard	Male	8.869908	9.55979	< 12mo	< 12mo	Yes
279	Yes	Reduced	Male	8.278581	10.34823	< 12mo	< 12mo	Yes
280	Yes	Standard	Female	9.36268	13.04205	< 12mo	>=12mo	Yes
281	Yes	Reduced	Male	11.30092	12.22076	< 12mo	>=12mo	Yes
282	No	Reduced	Female	9.55979	11.62944	< 12mo	< 12mo	Yes
283	No	Standard	Female	22	23	>=12mo	>=12mo	No
284	Yes	Reduced	Male	16.42576	20.10513	>=12mo	>=12mo	No
285	Yes	Standard	Male	7.522996	9.822602	< 12mo	< 12mo	Yes
286	Yes	Reduced	Female	16	17	>=12mo	>=12mo	Yes
287	Yes	Reduced	Female	7	8	< 12mo	< 12mo	Yes
288	Yes	Standard	Female	11	12	< 12mo	>=12mo	Yes
289	Yes	Standard	Female	9	12	< 12mo	>=12mo	Yes
290	Yes	Standard	Female	8.04862	9.658344	< 12mo	< 12mo	Yes
291	Yes	Reduced	Male	14.65177	15.80158	>=12mo	>=12mo	Yes
292	Yes	Reduced	Female	13.17346	14.32326	>=12mo	>=12mo	Yes
293	No	Standard	Female	6	10	< 12mo	< 12mo	Yes
294	Yes	Reduced	Female	36.69514	40.37451	>=12mo	>=12mo	No
295	Yes	Standard	Male	24	24	>=12mo	>=12mo	Yes
296	Yes	Reduced	Female	17	18	>=12mo	>=12mo	Yes
297	Yes	Standard	Male	8.70565	12.15506	< 12mo	>=12mo	Yes
298	No	Standard	Female	36	38	>=12mo	>=12mo	No
299	Yes	Reduced	Male	8.607096	11.82654	< 12mo	< 12mo	Yes
300	Yes	Reduced	Female	14.25756	15.1774	>=12mo	>=12mo	Yes
301	Yes	Standard	Male	9.001314	12.68068	< 12mo	>=12mo	Yes
302	Yes	Standard	Female	9.822602	13.50197	< 12mo	>=12mo	Yes
303	No	Standard	Male	6.800263	7.030223	< 12mo	< 12mo	Yes
304	Yes	Reduced	Female	7.884363	8.804205	< 12mo	< 12mo	Yes
305	Yes	Reduced	Female	6.504599	10.18397	< 12mo	< 12mo	Yes

306	Yes	Standard	Male	24	25	>=12mo	>=12mo	Yes
307	No	Reduced	Male	8.311432	11.9908	< 12mo	< 12mo	Yes
308	No	Reduced	Male	7.884363	9.264126	< 12mo	< 12mo	Yes
309	Yes	Standard	Female	41.09724	42.47701	>=12mo	>=12mo	No
310	Yes	Reduced	Female	7.424441	11.10381	< 12mo	< 12mo	Yes
311	No	Reduced	Male	11.95795	13.33771	< 12mo	>=12mo	Yes
312	Yes	Standard	Female	6	8	< 12mo	< 12mo	Yes
313	Yes	Standard	Female	6.537451	8.837056	< 12mo	< 12mo	Yes
314	Yes	Standard	Male	9.658344	10.57819	< 12mo	< 12mo	Yes
315	Yes	Standard	Female	6.636005	8.47569	< 12mo	< 12mo	Yes
316	Yes	Reduced	Female	6.044678	9.034165	< 12mo	< 12mo	Yes
317	Yes	Reduced	Male	19.80946	21.64915	>=12mo	>=12mo	Yes
318	Yes	Standard	Female	7.884363	9.494086	< 12mo	< 12mo	Yes
319	Yes	Reduced	Male	6.011827	8.771354	< 12mo	< 12mo	Yes
320	Yes	Reduced	Male	6.110381	9.55979	< 12mo	< 12mo	Yes
321	Yes	Standard	Male	13	15	>=12mo	>=12mo	Yes
322	No	Reduced	Female	10.05256	10.9724	< 12mo	< 12mo	Yes
323	No	Standard	Male	13.23916	14.159	>=12mo	>=12mo	Yes
324	Yes	Standard	Male	13.40342	14.78318	>=12mo	>=12mo	Yes
325	No	Reduced	Male	7.39159	7.39159	< 12mo	< 12mo	Yes
326	No	Standard	Male	7.785808	11.46518	< 12mo	< 12mo	Yes
327	Yes	Reduced	Female	7.490145	9.78975	< 12mo	< 12mo	Yes
328	Yes	Standard	Female	12.74639	14.81603	>=12mo	>=12mo	Yes
329	No	Standard	Female	9.36268	13.04205	< 12mo	>=12mo	Yes
330	Yes	Reduced	Female	6.997372	8.147175	< 12mo	< 12mo	Yes
331	Yes	Reduced	Male	14.61892	15.07884	>=12mo	>=12mo	Yes
332	No	Reduced	Male	7.588699	9.888305	< 12mo	< 12mo	Yes
333	No	Standard	Female	8.114324	11.79369	< 12mo	< 12mo	Yes
334	No	Standard	Female	10.9724	13.73193	< 12mo	>=12mo	Yes
335	No	Reduced	Female	8.639948	11.8594	< 12mo	< 12mo	Yes
336	No	Reduced	Male	20	23	>=12mo	>=12mo	Yes
337	No	Standard	Female	7.293036	10.9724	< 12mo	< 12mo	Yes
338	No	Standard	Male	6.73456	10.41393	< 12mo	< 12mo	Yes
339	No	Reduced	Male	15.90013	16.59001	>=12mo	>=12mo	Yes
340	No	Reduced	Male	16	17	>=12mo	>=12mo	Yes
341	No	Standard	Male	11.46518	12.84494	< 12mo	>=12mo	Yes
342	No	Reduced	Female	10.9067	11.59658	< 12mo	< 12mo	Yes
343	No	Standard	Male	10.18397	10.87385	< 12mo	< 12mo	Yes
344	No	Standard	Male	6.471748	9.921156	< 12mo	< 12mo	Yes
345	No	Reduced	Female	26	29	>=12mo	>=12mo	No
346	No	Reduced	Male	7.325887	11.00526	< 12mo	< 12mo	Yes
347	Yes	Reduced	Male	11.56373	12.9435	< 12mo	>=12mo	Yes
348	Yes	Standard	Male	11.95795	12.87779	< 12mo	>=12mo	Yes
349	No	Standard	Female	6.668857	9.888305	< 12mo	< 12mo	Yes
350	Yes	Standard	Female	7.950066	10.47963	< 12mo	< 12mo	Yes
351	No	Reduced	Female	6.537451	7.917214	< 12mo	< 12mo	Yes
352	Yes	Standard	Male	12	13	>=12mo	>=12mo	Yes
353	No	Reduced	Male	8.968463	12.41787	< 12mo	>=12mo	Yes
354	Yes	Reduced	Male	12.35217	14.19185	>=12mo	>=12mo	Yes
355	Yes	Standard	Male	15.53877	16.68857	>=12mo	>=12mo	No
356	Yes	Reduced	Female	36.26807	36.95795	>=12mo	>=12mo	No

357	No	Standard	Male	9	11	< 12mo	< 12mo	Yes
358	Yes	Reduced	Female	27.98949	28.67937	>=12mo	>=12mo	No
359	No	Standard	Female	6	7	< 12mo	< 12mo	Yes
360	Yes	Reduced	Female	7.555848	8.47569	< 12mo	< 12mo	Yes
361	No	Standard	Female	7.424441	11.10381	< 12mo	< 12mo	Yes
362	Yes	Reduced	Male	10.24967	11.16951	< 12mo	< 12mo	Yes
363	No	Standard	Female	57.95007	60.47963	>=12mo	>=12mo	No
364	No	Standard	Male	32	34	>=12mo	>=12mo	No
365	Yes	Reduced	Female	10.93955	14.38896	< 12mo	>=12mo	Yes
366	Yes	Standard	Male	7.358738	11.03811	< 12mo	< 12mo	Yes
367	Yes	Reduced	Male	7.358738	8.278581	< 12mo	< 12mo	Yes
368	Yes	Standard	Female	6.865966	10.54534	< 12mo	< 12mo	Yes
369	Yes	Reduced	Female	8.081471	9.691196	< 12mo	< 12mo	Yes
370	Yes	Standard	Male	7.720105	9.099869	< 12mo	< 12mo	Yes
371	Yes	Reduced	Female	7.39159	11.07096	< 12mo	< 12mo	Yes
372	No	Reduced	Female	23.71879	26.93824	>=12mo	>=12mo	Yes
373	No	Reduced	Female	8.837056	11.13666	< 12mo	< 12mo	Yes
374	Yes	Standard	Male	11.53088	15.21025	< 12mo	>=12mo	Yes
375	Yes	Standard	Male	7.950066	11.16951	< 12mo	< 12mo	Yes
376	Yes	Reduced	Male	13.37057	15.90013	>=12mo	>=12mo	Yes
377	No	Standard	Female	6.898818	8.278581	< 12mo	< 12mo	Yes
378	No	Standard	Male	15.44021	16.81997	>=12mo	>=12mo	Yes
379	No	Standard	Female	11.72799	13.10775	< 12mo	>=12mo	Yes
380	No	Reduced	Female	7.227333	10.67674	< 12mo	< 12mo	Yes
381	No	Reduced	Male	8.672799	9.36268	< 12mo	< 12mo	Yes
382	Yes	Reduced	Male	26.37976	27.06965	>=12mo	>=12mo	No
383	Yes	Standard	Male	6.143233	7.752957	< 12mo	< 12mo	Yes
384	No	Standard	Female	6.044678	9.724048	< 12mo	< 12mo	Yes
385	Yes	Reduced	Male	11.69514	15.37451	< 12mo	>=12mo	Yes
386	No	Reduced	Female	7.654402	8.804205	< 12mo	< 12mo	Yes
387	Yes	Standard	Female	48	48	>=12mo	>=12mo	No
388	Yes	Standard	Female	6.176084	8.47569	< 12mo	< 12mo	Yes
389	No	Standard	Male	27	28	>=12mo	>=12mo	No
390	No	Reduced	Female	28.81078	30.4205	>=12mo	>=12mo	No
391	No	Reduced	Female	29.76347	32.29303	>=12mo	>=12mo	No
392	No	Standard	Female	7.325887	10.7753	< 12mo	< 12mo	Yes
393	No	Standard	Female	7.621551	11.30092	< 12mo	< 12mo	Yes
394	No	Reduced	Female	12.08936	15.76873	>=12mo	>=12mo	Yes
395	No	Reduced	Female	14	15	>=12mo	>=12mo	No
396	No	Reduced	Female	14	18	>=12mo	>=12mo	Yes
397	No	Standard	Female	17	21	>=12mo	>=12mo	Yes
398	No	Reduced	Male	44	45	>=12mo	>=12mo	No
399	No	Standard	Female	7.194481	9.724048	< 12mo	< 12mo	Yes
400	No	Standard	Female	6.208936	9.198423	< 12mo	< 12mo	Yes
401	No	Reduced	Female	7.227333	9.067018	< 12mo	< 12mo	Yes
402	No	Standard	Male	10.18397	11.79369	< 12mo	< 12mo	Yes
403	No	Standard	Female	7.095926	10.31537	< 12mo	< 12mo	Yes
404	No	Standard	Male	7.720105	8.409987	< 12mo	< 12mo	Yes
405	Yes	Reduced	Female	19.5138	20.89356	>=12mo	>=12mo	Yes
406	No	Reduced	Male	7.194481	10.87385	< 12mo	< 12mo	Yes
407	No	Reduced	Male	9.36268	10.28252	< 12mo	< 12mo	Yes

408	No	Standard	Female	8.147175	9.067018	< 12mo	< 12mo	Yes
409	Yes	Reduced	Female	7.128778	8.04862	< 12mo	< 12mo	Yes
410	Yes	Standard	Female	7	10	< 12mo	< 12mo	Yes
411	Yes	Standard	Male	22.66754	25.42707	>=12mo	>=12mo	Yes
412	No	Standard	Female	9.526938	10.67674	< 12mo	< 12mo	Yes
413	No	Standard	Male	22.96321	24.11301	>=12mo	>=12mo	Yes
414	No	Reduced	Female	8.935611	12.38502	< 12mo	>=12mo	Yes
415	No	Reduced	Male	15.86728	16.78712	>=12mo	>=12mo	Yes
416	Yes	Reduced	Male	11.82654	14.12615	< 12mo	>=12mo	Yes
417	Yes	Reduced	Female	11.13666	14.81603	< 12mo	>=12mo	Yes
418	Yes	Standard	Male	10.44678	12.74639	< 12mo	>=12mo	Yes
419	Yes	Reduced	Female	6.636005	10.54534	< 12mo	< 12mo	Yes
420	Yes	Standard	Male	10.34823	12.41787	< 12mo	>=12mo	Yes
421	No	Standard	Female	7.293036	7.982917	< 12mo	< 12mo	Yes
422	Yes	Reduced	Female	12.18791	12.87779	>=12mo	>=12mo	Yes
423	Yes	Standard	Female	7.522996	11.20237	< 12mo	< 12mo	Yes
424	Yes	Reduced	Female	6.504599	9.264126	< 12mo	< 12mo	Yes
425	No	Reduced	Female	9.55979	10.47963	< 12mo	< 12mo	Yes
426	Yes	Standard	Female	14.0276	17.01708	>=12mo	>=12mo	Yes
427	No	Reduced	Male	10.38108	11.9908	< 12mo	< 12mo	Yes
428	Yes	Standard	Female	6.931669	10.61104	< 12mo	< 12mo	Yes
429	Yes	Standard	Female	11.03811	12.64783	< 12mo	>=12mo	Yes
430	Yes	Reduced	Female	6.636005	10.31537	< 12mo	< 12mo	Yes
431	Yes	Reduced	Female	6.274639	9.954008	< 12mo	< 12mo	Yes
432	Yes	Standard	Female	6.73456	9.264126	< 12mo	< 12mo	Yes
433	No	Reduced	Female	6.208936	9.888305	< 12mo	< 12mo	Yes
434	Yes	Standard	Male	39.71748	43.39685	>=12mo	>=12mo	No
435	No	Standard	Female	25.16426	26.54402	>=12mo	>=12mo	Yes
436	No	Reduced	Female	14.55322	16.16294	>=12mo	>=12mo	Yes
437	No	Standard	Male	6.767411	7.917214	< 12mo	< 12mo	Yes
438	No	Reduced	Male	12.81209	13.27201	>=12mo	>=12mo	Yes
439	Yes	Standard	Male	33.77136	34.92116	>=12mo	>=12mo	No
440	Yes	Reduced	Male	14.65177	16.03154	>=12mo	>=12mo	Yes
441	Yes	Reduced	Male	8.212878	8.90276	< 12mo	< 12mo	Yes
442	No	Standard	Male	8.04862	9.428384	< 12mo	< 12mo	Yes
443	Yes	Standard	Female	7.030223	8.180026	< 12mo	< 12mo	Yes
444	No	Reduced	Male	10.64389	12.25361	< 12mo	>=12mo	Yes
445	No	Standard	Male	9.13272	12.81209	< 12mo	>=12mo	Yes
446	No	Standard	Female	12.38502	16.06439	>=12mo	>=12mo	Yes
447	No	Reduced	Male	15.1774	18.39685	>=12mo	>=12mo	Yes
448	Yes	Reduced	Female	20.99212	22.8318	>=12mo	>=12mo	Yes
449	No	Reduced	Female	9.526938	12.28647	< 12mo	>=12mo	Yes
450	No	Standard	Female	12.51643	16.1958	>=12mo	>=12mo	Yes
451	No	Standard	Male	23.58739	26.57687	>=12mo	>=12mo	No
452	Yes	Reduced	Female	20.49934	24.17871	>=12mo	>=12mo	Yes
453	No	Reduced	Male	17.3456	21.02497	>=12mo	>=12mo	Yes
454	No	Standard	Female	8.541393	11.76084	< 12mo	< 12mo	Yes
455	No	Standard	Male	6.406045	10.08541	< 12mo	< 12mo	Yes
456	No	Reduced	Male	16.68857	17.60841	>=12mo	>=12mo	Yes
457	No	Standard	Female	6.964521	8.574245	< 12mo	< 12mo	Yes
458	No	Reduced	Male	16.88568	18.72536	>=12mo	>=12mo	No

459	No	Standard	Male	6.110381	7.720105	< 12mo	< 12mo	Yes
460	No	Reduced	Male	15.99869	18.52825	>=12mo	>=12mo	Yes
461	No	Reduced	Male	16.52431	20.20368	>=12mo	>=12mo	Yes
462	No	Standard	Female	10.41393	14.0933	< 12mo	>=12mo	Yes
463	Yes	Standard	Female	9.625493	12.61498	< 12mo	>=12mo	Yes
464	No	Reduced	Male	14.48752	16.3272	>=12mo	>=12mo	Yes
465	No	Reduced	Male	9.231275	10.61104	< 12mo	< 12mo	Yes
466	No	Standard	Male	22.43758	23.58739	>=12mo	>=12mo	Yes
467	Yes	Standard	Female	14.81603	16.42576	>=12mo	>=12mo	Yes
468	Yes	Reduced	Female	8.04862	9.428384	< 12mo	< 12mo	Yes
469	Yes	Reduced	Male	6.110381	7.260184	< 12mo	< 12mo	No
470	No	Reduced	Male	9.329829	13.23916	< 12mo	>=12mo	Yes
471	No	Standard	Male	8.377135	9.526938	< 12mo	< 12mo	Yes
472	Yes	Standard	Male	8.70565	11.69514	< 12mo	< 12mo	Yes
473	No	Standard	Female	28.90933	30.97898	>=12mo	>=12mo	No
474	No	Standard	Female	10.08541	12.61498	< 12mo	>=12mo	Yes
475	No	Reduced	Male	13.10775	16.78712	>=12mo	>=12mo	Yes
476	Yes	Standard	Female	29	30	>=12mo	>=12mo	No
477	Yes	Standard	Male	13.30486	16.75427	>=12mo	>=12mo	Yes
478	Yes	Standard	Female	10.31537	13.99474	< 12mo	>=12mo	Yes
479	No	Reduced	Male	48.09461	49.9343	>=12mo	>=12mo	No
480	No	Reduced	Male	37	39	>=12mo	>=12mo	Yes
481	Yes	Reduced	Male	7.687254	9.067018	< 12mo	< 12mo	Yes
482	Yes	Reduced	Female	31.73456	32.6544	>=12mo	>=12mo	No
483	No	Reduced	Male	9.231275	12.91064	< 12mo	>=12mo	Yes
484	No	Standard	Female	9.067018	12.74639	< 12mo	>=12mo	Yes
485	No	Standard	Female	46.09067	49.77004	>=12mo	>=12mo	No
486	Yes	Reduced	Male	14.06045	14.75033	>=12mo	>=12mo	Yes
487	Yes	Standard	Male	25.32852	27.62812	>=12mo	>=12mo	No
488	Yes	Standard	Female	8.935611	10.7753	< 12mo	< 12mo	Yes
489	Yes	Reduced	Male	53.51511	54.66491	>=12mo	>=12mo	No
490	Yes	Reduced	Female	15.27595	18.72536	>=12mo	>=12mo	Yes
491	No	Standard	Female	8.837056	10.21682	< 12mo	< 12mo	Yes
492	No	Reduced	Male	8.311432	8.311432	< 12mo	< 12mo	Yes
493	No	Standard	Male	22.07622	25.75559	>=12mo	>=12mo	Yes
494	No	Standard	Male	21.41919	23.48883	>=12mo	>=12mo	Yes
495	Yes	Standard	Female	13.89619	15.73587	>=12mo	>=12mo	Yes
496	Yes	Standard	Female	10.841	12.22076	< 12mo	>=12mo	Yes
497	No	Reduced	Female	27.46386	29.30355	>=12mo	>=12mo	No
498	Yes	Reduced	Male	9.067018	12.74639	< 12mo	>=12mo	Yes
499	No	Reduced	Male	8.672799	10.28252	< 12mo	< 12mo	Yes
500	Yes	Standard	Male	11.30092	14.98029	< 12mo	>=12mo	Yes
501	No	Reduced	Female	26.77398	28.84363	>=12mo	>=12mo	No
502	No	Reduced	Female	6.931669	8.771354	< 12mo	< 12mo	Yes
503	No	Standard	Male	25.0657	26.90539	>=12mo	>=12mo	Yes
504	No	Reduced	Male	6.110381	9.78975	< 12mo	< 12mo	Yes
505	Yes	Standard	Male	8.90276	12.35217	< 12mo	>=12mo	Yes
506	Yes	Reduced	Male	8.147175	11.13666	< 12mo	< 12mo	Yes
507	Yes	Reduced	Female	7.81866	8.508541	< 12mo	< 12mo	Yes
508	Yes	Standard	Male	7.752957	11.43233	< 12mo	< 12mo	Yes
509	Yes	Standard	Female	6.406045	10.08541	< 12mo	< 12mo	Yes

510	Yes	Standard	Female	11.53088	12.91064	< 12mo	>=12mo	Yes
511	No	Reduced	Male	15	16	>=12mo	>=12mo	Yes
512	No	Standard	Female	12.9435	16.62286	>=12mo	>=12mo	Yes
513	No	Standard	Female	6.143233	6.373193	< 12mo	< 12mo	Yes
514	No	Standard	Female	22.63469	26.31406	>=12mo	>=12mo	Yes
515	No	Standard	Female	15	19	>=12mo	>=12mo	No
516	No	Reduced	Female	8.04862	10.57819	< 12mo	< 12mo	Yes
517	No	Reduced	Male	22.70039	26.37976	>=12mo	>=12mo	Yes
518	No	Reduced	Male	7.358738	9.888305	< 12mo	< 12mo	Yes
519	No	Reduced	Female	6.964521	9.724048	< 12mo	< 12mo	Yes
520	No	Standard	Female	14.32326	16.3929	>=12mo	>=12mo	Yes
521	No	Standard	Female	9.855454	13.53482	< 12mo	>=12mo	Yes
522	No	Reduced	Female	6.833114	10.05256	< 12mo	< 12mo	Yes
523	No	Standard	Female	6	10	< 12mo	< 12mo	Yes
524	No	Reduced	Female	11.76084	12.91064	< 12mo	>=12mo	Yes
525	No	Reduced	Female	8.47569	11.03811	< 12mo	< 12mo	Yes
526	No	Standard	Female	12.77924	15.30881	>=12mo	>=12mo	Yes
527	No	Reduced	Female	14.75033	18.4297	>=12mo	>=12mo	Yes
528	Yes	Standard	Male	17.18134	19.25099	>=12mo	>=12mo	Yes
529	No	Reduced	Male	6.208936	7.81866	< 12mo	< 12mo	Yes
530	No	Reduced	Female	23.06176	23.9816	>=12mo	>=12mo	Yes
531	No	Reduced	Male	16.36005	18.19974	>=12mo	>=12mo	Yes
532	No	Standard	Female	7.654402	8.804205	< 12mo	< 12mo	Yes
533	Yes	Standard	Male	6.668857	10.11827	< 12mo	< 12mo	Yes
534	Yes	Reduced	Female	12.64783	16.09724	>=12mo	>=12mo	Yes
535	No	Standard	Female	6.110381	9.78975	< 12mo	< 12mo	Yes
536	Yes	Standard	Male	14.19185	15.34166	>=12mo	>=12mo	No
537	Yes	Reduced	Female	8.771354	12.45072	< 12mo	>=12mo	Yes
538	No	Standard	Female	6.964521	9.954008	< 12mo	< 12mo	Yes
539	Yes	Reduced	Female	19.87516	22.17477	>=12mo	>=12mo	No
540	Yes	Reduced	Male	7.293036	10.51248	< 12mo	< 12mo	Yes
541	No	Standard	Male	10.24967	10.93955	< 12mo	< 12mo	Yes
542	No	Standard	Male	6.898818	10.34823	< 12mo	< 12mo	Yes
543	Yes	Standard	Male	6.406045	7.785808	< 12mo	< 12mo	Yes
544	Yes	Reduced	Male	6	7	< 12mo	< 12mo	Yes
545	Yes	Reduced	Male	10.74244	14.42181	< 12mo	>=12mo	Yes
546	Yes	Reduced	Female	6.143233	7.982917	< 12mo	< 12mo	Yes
547	Yes	Reduced	Male	8.968463	12.64783	< 12mo	>=12mo	Yes
548	Yes	Standard	Male	10.24967	11.62944	< 12mo	< 12mo	Yes
549	Yes	Standard	Female	7.588699	11.26807	< 12mo	< 12mo	Yes
550	Yes	Standard	Male	7.917214	10.67674	< 12mo	< 12mo	Yes
551	Yes	Reduced	Male	21.78055	22.70039	>=12mo	>=12mo	No
552	Yes	Standard	Female	7.720105	11.39947	< 12mo	< 12mo	Yes
553	Yes	Reduced	Male	17.18134	20.86071	>=12mo	>=12mo	Yes
554	Yes	Reduced	Male	30.15769	30.84757	>=12mo	>=12mo	No
555	Yes	Standard	Female	7.030223	9.78975	< 12mo	< 12mo	Yes
556	Yes	Reduced	Male	15.60447	19.28384	>=12mo	>=12mo	Yes
557	No	Reduced	Female	57	59	>=12mo	>=12mo	No
558	No	Standard	Male	8	12	< 12mo	>=12mo	Yes
559	No	Reduced	Female	14.159	17.83837	>=12mo	>=12mo	Yes
560	Yes	Standard	Female	13.0092	15.30881	>=12mo	>=12mo	No

561	No	Reduced	Male	9.165571	12.84494	< 12mo	>=12mo	Yes
562	Yes	Reduced	Female	6.570302	8.180026	< 12mo	< 12mo	Yes
563	No	Standard	Female	17.37845	20.13798	>=12mo	>=12mo	Yes
564	No	Standard	Female	7.752957	9.13272	< 12mo	< 12mo	Yes
565	No	Standard	Female	7.063075	9.592641	< 12mo	< 12mo	Yes
566	No	Reduced	Male	11.49803	15.1774	< 12mo	>=12mo	Yes
567	No	Reduced	Female	16.88568	18.72536	>=12mo	>=12mo	Yes
568	No	Standard	Male	6.931669	10.15112	< 12mo	< 12mo	Yes
569	No	Reduced	Male	19.5795	20.7293	>=12mo	>=12mo	Yes
570	No	Reduced	Male	17.37845	18.75821	>=12mo	>=12mo	No
571	Yes	Standard	Male	21.87911	23.71879	>=12mo	>=12mo	Yes
572	No	Standard	Male	20.46649	21.38633	>=12mo	>=12mo	Yes
573	No	Reduced	Female	18.13404	21.8134	>=12mo	>=12mo	Yes
574	No	Reduced	Female	9	10	< 12mo	< 12mo	Yes
575	No	Reduced	Female	9.954008	10.87385	< 12mo	< 12mo	Yes
576	No	Standard	Male	21.02497	23.32457	>=12mo	>=12mo	Yes
577	No	Standard	Male	9.526938	12.74639	< 12mo	>=12mo	Yes
578	No	Reduced	Male	7.227333	9.986859	< 12mo	< 12mo	No
579	No	Standard	Male	9	10	< 12mo	< 12mo	Yes
580	No	Standard	Male	7.950066	7.950066	< 12mo	< 12mo	Yes
581	No	Standard	Male	6.406045	10.08541	< 12mo	< 12mo	Yes
582	No	Reduced	Female	11.10381	13.40342	< 12mo	>=12mo	Yes
583	No	Reduced	Male	10.93955	12.54928	< 12mo	>=12mo	Yes
584	No	Reduced	Female	8.672799	10.28252	< 12mo	< 12mo	Yes
585	No	Standard	Female	10.38108	14.06045	< 12mo	>=12mo	Yes
586	No	Reduced	Female	11.13666	12.97635	< 12mo	>=12mo	Yes
587	Yes	Standard	Male	14.25756	16.3272	>=12mo	>=12mo	Yes
588	No	Reduced	Female	9.986859	11.59658	< 12mo	< 12mo	Yes
589	Yes	Standard	Female	7.457293	10.21682	< 12mo	< 12mo	Yes
590	Yes	Reduced	Female	20.76216	22.37188	>=12mo	>=12mo	Yes
591	No	Reduced	Male	25.32852	26.70828	>=12mo	>=12mo	No
592	No	Reduced	Female	11.66229	12.81209	< 12mo	>=12mo	Yes
593	No	Standard	Female	36	38	>=12mo	>=12mo	No
594	No	Standard	Female	27.03679	27.95664	>=12mo	>=12mo	No
595	Yes	Standard	Female	7.325887	9.625493	< 12mo	< 12mo	Yes
596	No	Standard	Female	11.56373	12.25361	< 12mo	>=12mo	Yes
597	No	Standard	Female	24	26	>=12mo	>=12mo	Yes
598	No	Reduced	Male	9.231275	12.91064	< 12mo	>=12mo	Yes
599	No	Standard	Female	23.81735	24.27727	>=12mo	>=12mo	No
600	No	Reduced	Male	19.87516	21.02497	>=12mo	>=12mo	Yes
601	No	Reduced	Male	31.01183	31.70171	>=12mo	>=12mo	No
602	No	Reduced	Male	48	49	>=12mo	>=12mo	No
603	No	Standard	Female	29.10644	30.02628	>=12mo	>=12mo	No
604	No	Reduced	Male	48	50	>=12mo	>=12mo	No
605	Yes	Reduced	Male	19.77661	23.45598	>=12mo	>=12mo	No
606	No	Reduced	Male	24.11301	25.26281	>=12mo	>=12mo	No
607	Yes	Standard	Female	11.26807	13.56767	< 12mo	>=12mo	Yes
608	No	Reduced	Male	11.9908	14.06045	< 12mo	>=12mo	No
609	No	Standard	Female	7.227333	10.9067	< 12mo	< 12mo	Yes
610	No	Standard	Female	10.64389	14.32326	< 12mo	>=12mo	Yes
611	No	Standard	Male	7.358738	11.03811	< 12mo	< 12mo	Yes

612	No	Standard	Male	7.555848	8.70565	< 12mo	< 12mo	Yes
613	No	Reduced	Male	7.851511	9.461235	< 12mo	< 12mo	Yes
614	No	Standard	Male	15.76873	16.91853	>=12mo	>=12mo	Yes
615	Yes	Reduced	Male	12.31932	15.76873	>=12mo	>=12mo	Yes
616	Yes	Reduced	Male	9.822602	10.74244	< 12mo	< 12mo	Yes
617	Yes	Standard	Female	8.47569	9.625493	< 12mo	< 12mo	Yes
618	Yes	Standard	Male	29	33	>=12mo	>=12mo	No
619	Yes	Reduced	Male	7.720105	10.47963	< 12mo	< 12mo	Yes
620	No	Reduced	Female	13.89619	15.73587	>=12mo	>=12mo	No
621	Yes	Standard	Female	9.067018	12.74639	< 12mo	>=12mo	Yes
622	Yes	Reduced	Male	11.30092	12.91064	< 12mo	>=12mo	Yes
623	No	Standard	Male	6.208936	8.968463	< 12mo	< 12mo	Yes
624	No	Reduced	Male	9.691196	13.1406	< 12mo	>=12mo	Yes
625	Yes	Standard	Male	8.574245	11.33377	< 12mo	< 12mo	Yes
626	No	Standard	Female	14.42181	16.2615	>=12mo	>=12mo	Yes
627	No	Reduced	Male	9.724048	11.56373	< 12mo	< 12mo	Yes
628	No	Standard	Male	22.04336	22.50328	>=12mo	>=12mo	No
629	Yes	Standard	Male	16.65572	17.3456	>=12mo	>=12mo	Yes
630	Yes	Reduced	Male	17.44415	18.82392	>=12mo	>=12mo	Yes
631	Yes	Reduced	Female	20.56505	22.17477	>=12mo	>=12mo	Yes
632	Yes	Reduced	Male	10.841	12.45072	< 12mo	>=12mo	Yes
633	Yes	Reduced	Female	16.49146	17.64126	>=12mo	>=12mo	Yes
634	Yes	Standard	Female	7.785808	9.395533	< 12mo	< 12mo	Yes
635	Yes	Reduced	Female	11.16951	12.77924	< 12mo	>=12mo	Yes
636	No	Standard	Female	14.98029	16.59001	>=12mo	>=12mo	No
637	No	Standard	Female	12.97635	14.58607	>=12mo	>=12mo	Yes
638	Yes	Reduced	Female	11.36662	15.04599	< 12mo	>=12mo	Yes
639	Yes	Standard	Female	29.13929	30.51905	>=12mo	>=12mo	No
640	Yes	Reduced	Female	7.293036	9.36268	< 12mo	< 12mo	Yes
641	Yes	Standard	Male	6.241787	9.691196	< 12mo	< 12mo	Yes
642	No	Reduced	Female	15.07884	16.45861	>=12mo	>=12mo	Yes
643	No	Standard	Female	6.767411	7.457293	< 12mo	< 12mo	Yes
644	No	Reduced	Female	6.044678	9.724048	< 12mo	< 12mo	Yes
645	Yes	Standard	Female	6.110381	9.329829	< 12mo	< 12mo	Yes
646	Yes	Standard	Male	7.982917	10.74244	< 12mo	< 12mo	Yes
647	Yes	Reduced	Male	6.077529	8.147175	< 12mo	< 12mo	Yes
648	Yes	Reduced	Female	16.3272	19.54665	>=12mo	>=12mo	No
649	No	Standard	Female	6.011827	7.39159	< 12mo	< 12mo	Yes
650	Yes	Reduced	Male	7.522996	10.28252	< 12mo	< 12mo	Yes
651	Yes	Reduced	Male	20.69645	23.45598	>=12mo	>=12mo	Yes
652	Yes	Standard	Female	6.800263	8.869908	< 12mo	< 12mo	Yes
653	Yes	Reduced	Male	8.541393	11.9908	< 12mo	< 12mo	Yes
654	Yes	Standard	Male	11.82654	13.20631	< 12mo	>=12mo	Yes
655	No	Standard	Female	7.851511	9.231275	< 12mo	< 12mo	Yes
656	Yes	Standard	Female	11.16951	12.54928	< 12mo	>=12mo	Yes
657	Yes	Reduced	Female	8.508541	10.11827	< 12mo	< 12mo	Yes
658	Yes	Standard	Female	6.438896	9.428384	< 12mo	< 12mo	Yes
659	Yes	Reduced	Male	10.9067	14.12615	< 12mo	>=12mo	Yes
660	No	Reduced	Female	12.68068	14.98029	>=12mo	>=12mo	Yes
661	Yes	Standard	Male	7.950066	11.62944	< 12mo	< 12mo	Yes
662	Yes	Reduced	Male	16.68857	18.29829	>=12mo	>=12mo	Yes

663	No	Standard	Female	12.54928	14.159	>=12mo	>=12mo	Yes
664	Yes	Standard	Female	16.03154	16.49146	>=12mo	>=12mo	Yes
665	Yes	Reduced	Male	7	9	< 12mo	< 12mo	Yes
666	Yes	Standard	Female	14.52037	15.90013	>=12mo	>=12mo	Yes
667	Yes	Standard	Female	8.70565	9.855454	< 12mo	< 12mo	Yes
668	Yes	Reduced	Male	19.08673	19.77661	>=12mo	>=12mo	No
669	Yes	Reduced	Female	6.833114	7.522996	< 12mo	< 12mo	Yes
670	Yes	Reduced	Male	9.067018	11.36662	< 12mo	< 12mo	Yes
671	Yes	Standard	Female	7.588699	9.888305	< 12mo	< 12mo	Yes
672	Yes	Reduced	Female	7.325887	8.70565	< 12mo	< 12mo	Yes
673	Yes	Standard	Female	6.668857	8.278581	< 12mo	< 12mo	Yes
674	Yes	Standard	Male	6.274639	9.954008	< 12mo	< 12mo	Yes
675	Yes	Standard	Male	25	27	>=12mo	>=12mo	No
676	Yes	Reduced	Male	17.64126	18.79107	>=12mo	>=12mo	Yes
677	Yes	Reduced	Male	6.668857	10.34823	< 12mo	< 12mo	Yes
678	Yes	Reduced	Male	6.668857	10.34823	< 12mo	< 12mo	Yes
679	Yes	Standard	Male	10.01971	10.93955	< 12mo	< 12mo	Yes
680	Yes	Standard	Female	10.80815	14.48752	< 12mo	>=12mo	Yes
681	Yes	Reduced	Female	12.25361	13.63338	>=12mo	>=12mo	Yes
682	Yes	Standard	Female	21.51774	22.20762	>=12mo	>=12mo	No
683	Yes	Reduced	Female	8.968463	9.888305	< 12mo	< 12mo	Yes
684	Yes	Standard	Female	11.79369	12.71353	< 12mo	>=12mo	Yes
685	No	Reduced	Male	7.81866	9.658344	< 12mo	< 12mo	Yes
686	Yes	Standard	Female	7.227333	9.756899	< 12mo	< 12mo	Yes
687	No	Reduced	Male	7.81866	11.49803	< 12mo	< 12mo	Yes
688	Yes	Standard	Male	6.701708	7.851511	< 12mo	< 12mo	Yes
689	No	Reduced	Male	20.20368	21.8134	>=12mo	>=12mo	Yes
690	No	Reduced	Female	7.687254	8.377135	< 12mo	< 12mo	Yes
691	Yes	Reduced	Male	10.80815	12.41787	< 12mo	>=12mo	Yes
692	No	Standard	Male	10.64389	12.25361	< 12mo	>=12mo	Yes
693	Yes	Standard	Female	11	13	< 12mo	>=12mo	Yes
694	Yes	Standard	Male	12.45072	15.67017	>=12mo	>=12mo	Yes
695	Yes	Reduced	Male	10.87385	12.02365	< 12mo	>=12mo	Yes
696	Yes	Standard	Female	7.851511	10.61104	< 12mo	< 12mo	Yes
697	No	Reduced	Female	7.982917	10.51248	< 12mo	< 12mo	Yes
698	Yes	Standard	Female	11.59658	14.12615	< 12mo	>=12mo	Yes
699	Yes	Reduced	Male	13	14	>=12mo	>=12mo	Yes
700	Yes	Standard	Male	8.508541	9.658344	< 12mo	< 12mo	Yes
701	No	Standard	Female	17.11564	19.41524	>=12mo	>=12mo	Yes
702	Yes	Reduced	Male	8.804205	10.18397	< 12mo	< 12mo	Yes
703	Yes	Standard	Male	12.15506	13.53482	>=12mo	>=12mo	Yes
704	No	Reduced	Male	9	11	< 12mo	< 12mo	Yes
705	Yes	Standard	Male	9.954008	12.02365	< 12mo	>=12mo	Yes
706	No	Reduced	Male	9.231275	12.91064	< 12mo	>=12mo	Yes
707	Yes	Reduced	Female	8.869908	10.70959	< 12mo	< 12mo	Yes
708	Yes	Reduced	Female	11.33377	12.48357	< 12mo	>=12mo	Yes
709	Yes	Reduced	Female	6.997372	10.67674	< 12mo	< 12mo	Yes
710	Yes	Standard	Female	9.888305	11.03811	< 12mo	< 12mo	Yes
711	Yes	Standard	Male	24.44153	26.51117	>=12mo	>=12mo	Yes
712	No	Reduced	Male	5.978975	9.428384	< 12mo	< 12mo	Yes
713	No	Reduced	Female	5.946124	9.625493	< 12mo	< 12mo	Yes

714	Yes	Reduced	Female	11.76084	13.37057	< 12mo	>=12mo	Yes
715	No	Reduced	Male	12.15506	14.45467	>=12mo	>=12mo	Yes
716	No	Reduced	Female	9.724048	13.40342	< 12mo	>=12mo	Yes
717	No	Standard	Female	22.27333	22.27333	>=12mo	>=12mo	Yes
718	No	Standard	Male	12.35217	14.65177	>=12mo	>=12mo	Yes
719	No	Standard	Male	36	36	>=12mo	>=12mo	No
720	No	Standard	Male	12.61498	16.29435	>=12mo	>=12mo	Yes
721	No	Standard	Male	7.358738	9.888305	< 12mo	< 12mo	Yes
722	No	Reduced	Female	26.90539	28.77792	>=12mo	>=12mo	No
723	No	Reduced	Female	6.044678	9.264126	< 12mo	< 12mo	Yes
724	No	Standard	Male	13.0092	15.76873	>=12mo	>=12mo	Yes
725	No	Reduced	Female	6.800263	10.47963	< 12mo	< 12mo	Yes
726	No	Reduced	Male	6.701708	9.001314	< 12mo	< 12mo	Yes
727	No	Reduced	Male	7.457293	8.837056	< 12mo	< 12mo	Yes
728	No	Standard	Male	6.767411	8.837056	< 12mo	< 12mo	Yes
729	Yes	Reduced	Male	13.89619	16.88568	>=12mo	>=12mo	Yes
730	No	Standard	Female	11.26807	13.33771	< 12mo	>=12mo	Yes
731	No	Reduced	Female	8.90276	12.58213	< 12mo	>=12mo	Yes
732	No	Reduced	Male	30.91327	33.21288	>=12mo	>=12mo	Yes
733	Yes	Standard	Female	27.85808	30.15769	>=12mo	>=12mo	Yes
734	No	Reduced	Male	7.260184	7.720105	< 12mo	< 12mo	Yes
735	Yes	Reduced	Male	24	26	>=12mo	>=12mo	Yes
736	No	Standard	Male	6.701708	10.38108	< 12mo	< 12mo	Yes
737	No	Reduced	Female	42.77267	45.07227	>=12mo	>=12mo	No
738	Yes	Standard	Male	12.64783	13.56767	>=12mo	>=12mo	Yes
739	Yes	Standard	Female	24.86859	26.24836	>=12mo	>=12mo	Yes
740	No	Reduced	Female	27.26675	27.49672	>=12mo	>=12mo	Yes
741	No	Standard	Male	11.36662	12.0565	< 12mo	>=12mo	Yes
742	No	Reduced	Male	19	20	>=12mo	>=12mo	No
743	Yes	Reduced	Female	6	9	< 12mo	< 12mo	Yes
744	Yes	Reduced	Female	12	14	>=12mo	>=12mo	Yes
745	Yes	Standard	Male	10.54534	11.23522	< 12mo	< 12mo	Yes
746	Yes	Standard	Male	9.822602	10.74244	< 12mo	< 12mo	Yes
747	No	Standard	Male	7.654402	11.33377	< 12mo	< 12mo	Yes
748	Yes	Reduced	Male	24.63863	26.47832	>=12mo	>=12mo	Yes
749	No	Reduced	Female	9.264126	11.33377	< 12mo	< 12mo	Yes
750	Yes	Standard	Female	6.701708	10.38108	< 12mo	< 12mo	Yes
751	Yes	Standard	Male	48	51	>=12mo	>=12mo	No
752	Yes	Standard	Female	11.69514	13.0749	< 12mo	>=12mo	Yes
753	No	Reduced	Female	7.884363	10.64389	< 12mo	< 12mo	Yes
754	Yes	Standard	Female	6.537451	8.147175	< 12mo	< 12mo	Yes
755	Yes	Standard	Female	36	37	>=12mo	>=12mo	No
756	No	Reduced	Male	11.23522	12.61498	< 12mo	>=12mo	Yes
757	Yes	Reduced	Male	6.800263	8.180026	< 12mo	< 12mo	No
758	Yes	Standard	Male	15.53877	17.37845	>=12mo	>=12mo	No
759	No	Reduced	Male	25.75559	26.44547	>=12mo	>=12mo	Yes
760	Yes	Reduced	Female	28.21945	29.82917	>=12mo	>=12mo	No
761	Yes	Standard	Female	24.24442	25.85414	>=12mo	>=12mo	Yes
762	Yes	Standard	Male	14.81603	18.03548	>=12mo	>=12mo	Yes
763	Yes	Standard	Female	16.88568	18.72536	>=12mo	>=12mo	Yes
764	Yes	Standard	Male	18.92247	20.76216	>=12mo	>=12mo	Yes

765	No	Standard	Male	11.20237	12.12221	< 12mo	>=12mo	Yes
766	Yes	Reduced	Male	6.865966	10.31537	< 12mo	< 12mo	Yes
767	No	Reduced	Male	10.41393	14.0933	< 12mo	>=12mo	Yes
768	No	Reduced	Male	10.38108	14.06045	< 12mo	>=12mo	Yes
769	No	Reduced	Male	8.114324	11.79369	< 12mo	< 12mo	Yes
770	No	Standard	Male	21.35348	25.03285	>=12mo	>=12mo	Yes
771	No	Reduced	Female	7.260184	10.93955	< 12mo	< 12mo	Yes
772	No	Reduced	Female	14.98029	16.81997	>=12mo	>=12mo	Yes
773	Yes	Standard	Female	18.52825	21.28778	>=12mo	>=12mo	Yes
774	Yes	Reduced	Female	10.9067	12.97635	< 12mo	>=12mo	Yes
775	No	Standard	Male	9	12	< 12mo	>=12mo	Yes
776	No	Reduced	Female	8.47569	12.15506	< 12mo	>=12mo	Yes
777	No	Standard	Female	7.293036	10.28252	< 12mo	< 12mo	Yes
778	Yes	Standard	Male	55.91327	59.59264	>=12mo	>=12mo	No
779	No	Reduced	Male	30.58476	34.26413	>=12mo	>=12mo	No
780	No	Reduced	Male	24.63863	28.318	>=12mo	>=12mo	No
781	No	Reduced	Male	56	58	>=12mo	>=12mo	No
782	Yes	Standard	Male	13.30486	16.98423	>=12mo	>=12mo	Yes
783	Yes	Standard	Male	20.79501	23.55453	>=12mo	>=12mo	Yes
784	Yes	Standard	Male	42.93692	43.85677	>=12mo	>=12mo	No
785	No	Reduced	Male	7.720105	11.16951	< 12mo	< 12mo	Yes
786	Yes	Standard	Male	8.968463	11.26807	< 12mo	< 12mo	Yes
787	Yes	Reduced	Male	20.82786	24.27727	>=12mo	>=12mo	Yes
788	Yes	Reduced	Female	11.36662	13.43627	< 12mo	>=12mo	Yes
789	Yes	Standard	Male	22.86465	25.16426	>=12mo	>=12mo	Yes
790	Yes	Standard	Male	11.9251	13.99474	< 12mo	>=12mo	Yes
791	Yes	Reduced	Male	20.00657	21.84625	>=12mo	>=12mo	Yes
792	No	Standard	Male	19.02103	20.86071	>=12mo	>=12mo	Yes
793	Yes	Standard	Female	7.522996	10.28252	< 12mo	< 12mo	Yes
794	Yes	Reduced	Female	36	37	>=12mo	>=12mo	No
795	No	Reduced	Female	7.522996	11.20237	< 12mo	< 12mo	Yes
796	Yes	Reduced	Female	20	23	>=12mo	>=12mo	Yes
797	Yes	Reduced	Male	20.43364	23.19317	>=12mo	>=12mo	No
798	Yes	Standard	Male	19.05388	22.27333	>=12mo	>=12mo	Yes
799	No	Standard	Male	23.71879	25.32852	>=12mo	>=12mo	Yes
800	Yes	Reduced	Male	13.60053	17.2799	>=12mo	>=12mo	Yes
801	Yes	Standard	Male	14.159	16.45861	>=12mo	>=12mo	Yes

breastf_ex	team	weight_ad	height_adr	muac_adm	weight_exi	height_exi	muac_exit	Cob_adm
Yes	Team 2	6.65	66.65	114.5	7.35	67.4	130.5	
Yes	Team 2	7	75.9	119.5	8.35	78.45	133.5	
Yes	Team 2	5.05	61.2	105.5	6.3	66.5	119	
Yes	Team 2	7.25	73.95	119.5	8.45	76.95	126.5	204.476
Yes	Team 2	6	68.35	116	7.5	71.45	131.5	
Yes	Team 2	4.75	64	98	5.6	68.05	109	
Yes	Team 2	6.15	70.65	111.5	7.35	72.25	127.5	
Yes	Team 2	6.75	71.95	111.5	8.15	75.45	127.5	
Yes	Team 2	5.85	71.85	113.5				
Yes	Team 1	5.1	62.4	110.5	6.05	66.75	117.5	
No	Team 2	9.2	95	99.5	11.9	96.35	136.5	
Yes	Team 2	6.7	73.45	108	7.3	76.85	113.5	
Yes	Team 2	5.05	61.75	108.5	6.3	65.45	125.5	90.118
No	Team 2	7.55	77.15	114.5	8.55	78	130.5	
No	Team 2	10.2	92.7	131.5	11.4	93.45	140.5	
No	Team 2	8	79.45	114.5	8.9	80.25	129.5	
No	Team 2	10.1	93.3	132.5	11.2	93.25	140.5	
Yes	Team 2	5.25	61.75	112.5	5.95	63.85	125.5	
No	Team 2	8.15	82.25	123.5	8.8	83.4	128.5	
No	Team 2	7.55	74.85	114.5	8.4	75.65	130.5	
No	Team 1	7	75.15	116	8.4	75.7	128	
Yes	Team 2	5.05	61.45	113.5	6.6	63.65	127.5	179.304
Yes	Team 2	8.6	82.95	128.5	9.9	83.95	135.5	159.257
Yes	Team 2	5.4	64.75	109.5	6	66.85	117	
No	Team 2	6.25	68.15	113.5	6.3	68.35	116.5	
Yes	Team 2	5.85	66.3	115.5	7.65	69.95	134.5	153.361
No	Team 2	8.25	80.95	113.5	9.9	83.95	133.5	295.434
No	Team 2	9.9	90.05	129.5	12	90	145.5	174.934
No	Team 2	7.6	78.35	114.5	9.6	81.05	136.5	630.073
Yes	Team 2	5.2	63.35	112.5	6.1	68.35	116.5	98.024
Yes	Team 2	7	72.95	124	8	74.45	140	150.534
No	Team 1	5.5	65.5	119	6.45	70.55	121	
Yes	Team 2	7.6	78.6	113.5	8.8	80.45	126.5	177.703
No	Team 2	9.1	90.05	118.5	10.4	93.15	130	256.31
Yes	Team 2	5.8	66.95	116.5	7.35	69.85	133.5	54.231
No	Team 2	7.55	78.75	116.5	8.95	79.45	141	463.067
Yes	Team 2	6.2	70.65	110.5	7.6	71.65	132.5	225.209
Yes	Team 2	6.35	72.35	104.5	7	74.75	125.5	96.994
Yes	Team 2	7.25	72.25	113.5	7.75	74.55	122.5	192.183
Yes	Team 2	5.7	67.45	105.5	7.75	72.35	134.5	94.852
No	Team 2	7.45	74.75	116.5	8.85	75.65	139.5	830.527
Yes	Team 2	6.15	68.75	113.5	7.5	70.25	131.5	168.227
No	Team 1	5.5	64.5	114	6.4	68.05	121.5	
No	Team 2	7	71.45	114.5	6.45	71.75	109.5	
Yes	Team 2	5	61.85	114.5	6	64.45	126.5	
No	Team 2	6.95	75.25	115	8.2	77	127.5	327.508
Yes	Team 2	6.45	72.45	109.5	7.5	75.7	122	176.335
Yes	Team 2	5.95	69.45	104.5	8.3	72.75	135.5	233.034
No	Team 2	8.4	82.05	118.5	9.6	85.4	128.5	330.324
No	Team 2	9.55	86.9	127.5	12.1	87.25	149.5	254.66

Yes	Team 2	6.45	70.95	119.5	7.8	72.25	132.5	145.519
Yes	Team 2	7.35	72.25	112.5	8.2	73.8	130.5	96.893
Yes	Team 2	8.1	78.75	111.5	9.6	80.95	133.5	83.741
Yes	Team 1	6.15	63	113.5	7.1	68.2	128	
Yes	Team 2	5.85	67.25	111.5	7.6	71.95	132.5	222.572
Yes	Team 2	6.2	69.65	124.5	7.25	73	134.5	181.466
Yes	Team 2	4.85	59.1	108.5	6	61.35	126.5	
No	Team 2	13.1	107.45	130.5	12.9	108.15	132.5	238.832
Yes	Team 2	5.9	66.95	114.5	6.4	67.25	117.5	
No	Team 2	7.15	77.55	123.5	8.05	79.85	134.5	
Yes	Team 2	4.4	60	98.5	5.5	64.55	110.5	95.857
Yes	Team 2	5.75	65.2	119.5	6.85	69	126.5	170.909
Yes	Team 2	6.45	72.4	114.5	7	75.35	121.5	
No	Team 2	8.15	79.95	122.5	9.2	80.9	135	312.55
No	Team 1	5.4	65.4	106	5.85	68.25	113.5	
No	Team 2	7.9	80.65	123.5	9	82.75	136	
Yes	Team 2	5.75	67.1	112.5	6.9	68.25	129.5	309.202
Yes	Team 2	4.4	63.2	98.5	5.65	65.45	113.5	
Yes	Team 2	5.3	64.4	116.5	6.3	65.25	127.5	175.012
Yes	Team 2	7.1	74.75	123.5	8.65	75.95	141.5	222.28
No	Team 2	5.45	64.95	113.5	6.8	67.45	126	385.865
Yes	Team 2	6.35	71.95	112.5	7.7	73.25	129.5	219.812
Yes	Team 2	5.65	66.95	110.5	7.3	69	127.5	306.212
Yes	Team 2	7.5	77.45	113.5	9.4	78.2	142.5	65.748
Yes	Team 2	4.7	63.45	102.5	6.3	67.75	125.5	213.568
Yes	Team 1	5.2	63.2	112	6.2	68.4	118	
Yes	Team 2	6.05	69.35	106.5	7.4	73.35	125	159.86
No	Team 2	7.3	76.25	125.5	8.5	77.45	133.5	
Yes	Team 2	5.3	64.95	112.5	6.3	66.25	125.5	196.3
Yes	Team 2	7.45	78.25	121.5	8.7	79.95	132.5	406.534
No	Team 2	7.5	71.95	113.5				
Yes	Team 2	5.45	66.45	112.5	6.15	68	117	
Yes	Team 1	5.2	64.5	114	6.8	66.5	136	
Yes	Team 1	4.3	59.3	113	5.4	64.45	116.5	
No	Team 1	4.9	61	107	5.05	64.25	111	
Yes	Team 1	5.5	65.75	107	6.6	70.2	115.5	
Yes	Team 1	4.65	60.15	110	4.2	60.95	101	
No	Team 1	5.9	68	111	7.55	70.25	130.5	
Yes	Team 1	5.3	61.95	112	6.55	64.75	131.5	
Yes	Team 1	5.1	62.45	113.5	6	67.5	116.5	
No	Team 1	5.35	61	113.5	5.7	62.15	117.5	
Yes	Team 1	6.4	67.5	112.5	7.5	68.85	133.5	60.861
Yes	Team 1	6.9	73	123.5	8.65	73.9	140.5	209.393
No	Team 1	7.4	76.8	113.5				
Yes	Team 1	5.45	62.25	110.5	6.4	64	125	75.7
Yes	Team 1	5.6	65.35	112.5	6.95	67	126.5	
No	Team 1	7.7	77.95	116.5	8.4	80.05	121.5	
Yes	Team 1	6.05	68.95	113.5	7.75	72.7	134.5	176.525
No	Team 1	7.05	77	104.5	8.8	78.65	125.5	
No	Team 1	5.15	63	110.5	5.8	64.35	118.5	
Yes	Team 1	4.85	61	101	6.95	65.5	127.5	158.909

Yes	Team 1	4.4	57.5	91.5	6.45	62.25	127.5	
Yes	Team 1	7.4	77.05	118.5	8.5	78.05	128.5	219.257
Yes	Team 1	5.35	66.45	112	7	68	127.5	159.388
Yes	Team 1	6	66.65	124.5	6.95	67.5	136.5	
Yes	Team 1	7	68.95	113.5	7.7	70.5	126	87.874
No	Team 1	5.85	65	114.5	6.25	67.9	114.5	
Yes	Team 1	4.15	58.15	97	6	65.5	114	164.148
Yes	Team 1	5.8	65.55	117.5	7.1	69.5	126.5	174.004
Yes	Team 1	5.7	67	114.5	7.35	71.65	135.5	138.741
Yes	Team 1	5.2	61.2	113.5	5.8	64.2	118.5	281.631
No	Team 1	5	62.3	105	5.55	64	109	
Yes	Team 1	5.2	63	110	6.2	68.7	117	340.681
Yes	Team 1	5.65	63	113.5	6.7	65.35	125	
Yes	Team 1	4.8	60.8	106.5	5.05	63	107	191.159
Yes	Team 1	5.9	63.45	113.5	6.15	65	110.5	523.764
Yes	Team 1	5.15	63.5	107.5	5.6	69.2	109	
Yes	Team 1	5	62	112.5	5.95	66.5	122.5	
Yes	Team 1	5.2	61.5	104.5	6.2	66.5	125	206.332
Yes	Team 1	5.3	63.05	113.5	5.2	65	110.5	377.765
Yes	Team 1	4.5	57.25	105	4.7	60.55	112.5	
Yes	Team 1	5.3	63.85	105.5	6.35	65.45	131.5	286.273
Yes	Team 1	5.1	59.5	114	5.35	60.85	114.5	193.729
Yes	Team 1	5.65	61.85	110	5.8	64.75	107	129.457
Yes	Team 1	4.3	57	108	5.85	61	134.5	
Yes	Team 1	4.9	62.4	105	6.35	64.75	126.5	
Yes	Team 1	4.9	58.15	109.5	6.2	62.15	126.5	
Yes	Team 1	5.7	62.45	112.5	5.55	63.3	115.5	157.981
Yes	Team 1	6.85	72.45	118.5	6.9	73.45	122.5	
Yes	Team 1	5.8	65.5	114.5	6.9	68.45	126	95.849
Yes	Team 1	6.5	70.45	118.5	7.9	72.45	128.5	416.621
Yes	Team 1	6.3	70.05	116	7.55	71.65	133	
Yes	Team 1	4.65	59.5	112.5	5.6	63.15	115.5	
No	Team 1	7.8	77	114	8.8	79.35	125.5	318.767
Yes	Team 1	5.5	65.5	114	7.1	66.5	134.5	
Yes	Team 1	6.5	71.1	119.5	8.1	73.35	133	81.236
Yes	Team 1	6.85	72.95	112.5	8.4	74.65	132	337.486
Yes	Team 1	5.1	63.2	113.5	6.55	64.65	133.5	204.548
No	Team 1	5.5	65.25	113.5	7	69	125.5	
Yes	Team 1	6.25	70	131	7.3	70.5	137	
No	Team 1	6	65.65	113.5	6.15	66.5	120	
Yes	Team 1	4.5	61	90	5.4	65.2	105	140.644
Yes	Team 1	6.15	68.95	110.5	7.85	71.15	133.5	133.234
Yes	Team 1	5.75	70	105	6.75	72.25	113.5	485.561
Yes	Team 1	5.2	61.45	107.5	5.2	62.65	106.5	
No	Team 1	5.45	64.85	98.5	6	65.65	114.5	
Yes	Team 1	5.15	63	113.5	6.3	64.55	133.5	66.603
Yes	Team 1	6.55	73	122.5	7.8	73.25	133.5	518.132
Yes	Team 1	5.3	62.75	111	6.8	66.25	127.5	
Yes	Team 1	5.5	61	114	6.15	63.3	127	
No	Team 1	5	60.3	108	5.95	65.2	119.5	
Yes	Team 1	7.4	73.3	114	8.8	73.5	130	

Yes	Team 1	5.1	62	110.5	6.55	65.3	128.5	
Yes	Team 1	4.3	59.5	98	6	66.35	119	
No	Team 1	7.3	74.5	110	8.1	75.7	125	
Yes	Team 1	4.7	62.1	106	6.1	67.45	116	
Yes	Team 1	5.5	64.6	111	6.6	70	116	
No	Team 1	6.6	72.5	122	7	72.75	125.5	
Yes	Team 1	5.6	67	106.5	7.05	68.25	128.5	
Yes	Team 1	5.2	64.6	95	7.4	68.4	132.5	
Yes	Team 1	6.95	70.5	114	7.4	70.95	126.5	
Yes	Team 1	4.7	59.05	112.5	4.9	61.45	113.5	
No	Team 1	8.2	80.1	124.5	9.5	81	140	
Yes	Team 1	5.75	65.05	111.5	5.95	66.5	117.5	
Yes	Team 1	4.9	60.95	110.5	5.15	62.6	112.5	
No	Team 1	5.6	63.85	113.5	5.35	65.35	108	
No	Team 1	5	65.5	99.5	5.5	66.35	108.5	
No	Team 1	5.6	65.55	90.5	6.65	69.95	113	
Yes	Team 1	5.9	65.1	114.5	7.1	69.45	127.5	
Yes	Team 1	6.4	72.15	105.5	8.1	73	135.5	
Yes	Team 1	6.85	72.35	123.5	7.85	73.5	133	
Yes	Team 1	6.7	73	115.5	8.7	78.5	137.5	
Yes	Team 1	5.1	64.05	112.5	6.3	65.4	127.5	
Yes	Team 1	7.4	77.45	124	8.9	78.95	142	
Yes	Team 1	5.1	62.65	110	6.6	65.85	129.5	
Yes	Team 1	6.6	70.25	113.5	8.15	74.45	126.5	
Yes	Team 1	5.85	67.45	113.5	7.6	71.7	135	
Yes	Team 1	5.8	68.3	118	7.65	71	136.5	232.593
Yes	Team 1	4.75	59.55	110	6	61.95	125.5	
Yes	Team 1	7.4	78.35	113.5	9.1	79	135	
No	Team 1	6.3	71	115.5	7.1	72.65	124.5	
Yes	Team 1	5.5	65.45	112.5	6.45	69	120	
Yes	Team 1	4.7	62.35	104.5	6.4	66.6	125.5	
No	Team 1	6.3	71.5	110	6.6	72.15	113.5	
Yes	Team 1	6.75	71	113.5	8.35	74.4	130.5	55.403
Yes	Team 1	4.5	57.7	112	5.5	60	125.5	
No	Team 1	7.55	74.5	112.5	8.5	77.6	122	
Yes	Team 1	7.15	69.5	114.5	8.3	72.6	126.5	177.01
No	Team 1	5.6	66	111.5	6.05	66.95	120.5	
Yes	Team 1	5.85	67.35	104.5	7.85	70.4	126	
Yes	Team 1	6.05	65.15	113.5	7.2	67.25	130.5	133.925
No	Team 1	6.35	69	112.5	7.8	70.45	134.5	163.406
No	Team 1	5.45	69.2	105	7.15	71.95	123	
Yes	Team 1	6.6	71.4	113.5	8	74.45	128.5	107.4175
Yes	Team 1	6.3	65	113.5	7.55	67.35	130.5	116.122
No	Team 1	6.3	70.75	117	6.75	71.7	119.5	
Yes	Team 1	5.5	63.4	112.5	6.8	67.5	127.5	145.493
No	Team 1	6.7	78	105.5	8.1	78.15	118.5	
Yes	Team 1	6.5	72.05	115.5	8.35	76.5	133.5	99.609
No	Team 1	7.05	74.95	113.5	7	75.15	113.5	
No	Team 1	7.2	75.45	113.5	8	75.65	124	
Yes	Team 1	5.5	64.45	108.5	6.5	69.45	113.5	140.225
No	Team 1	4.75	61.5	109.5	6.5	64.5	128.5	

Yes	Team 1	7.3	74.2	121	7.55	75.5	121.5	299.666
No	Team 1	8.2	78.1	114.5	9.65	79.35	127.5	336.065
No	Team 1	5.85	66.35	108.5	7.05	70.65	126	
No	Team 1	8.7	83.4	128	9.2	85	130	226.063
Yes	Team 1	5.55	67	114.5	6.25	69.5	117.5	178.616
Yes	Team 1	6.55	68	111.5	6.65	70	120.5	181.402
Yes	Team 1	5.4	64.45	111.5	6.9	67.95	125	100.959
Yes	Team 1	5.95	63.95	114.5	7.15	64.75	133.5	
Yes	Team 1	6.8	71.5	115.5	6.85	72.65	120	
No	Team 1	8.6	85.45	120.5	10	89.4	130.5	239.57
Yes	Team 1	5.05	64	106	6.6	70	121	
Yes	Team 1	5.65	67.8	110		70.75	114	
Yes	Team 1	5.8	62.75	114.5	6.1	63.2	119	
Yes	Team 1	4.4	61	112.5	4.8	61.5	112	
Yes	Team 1	5.2	63.6	111	6.75	67.05	125	
Yes	Team 1	6.1	65.3	112	7.6	65.9	131	
Yes	Team 1	6.9	75	111	9.4	75	144	160.063
Yes	Team 1	6.2	67	120	7.3	67.55	133	
No	Team 1	8.3	80	108	8.4	82.5	118	
Yes	Team 1	5.3	65.8	107	8.15	68.4	143	
Yes	Team 1	5.7	66.4	114	6.45	68.35	122	
Yes	Team 1	6.2	66.5	111	6.85	68.35	117	
Yes	Team 1	5.1	60	109	6.7	64.95	125	
Yes	Team 1	6.4	66.1	112.5	6.8	67.5	125	
Yes	Team 1	5.7	63.7	114	6.5	66.45	125.5	
Yes	Team 1	5.55	61.5	116	6.45	68.65	118.5	
Yes	Team 1	6.9	70.6	113	8.45	73.9	134.5	
No	Team 1	5.35	62	112	5.85	65.35	118.5	
Yes	Team 1	6.5	70.1	111.5	7.6	71.5	131.5	
Yes	Team 1	4.05	57	97	5.55	60.75	127.5	
Yes	Team 1	5.5	63.3	111	5.6	64.25	109.5	
Yes	Team 1	4.2	62.9	98	6.55	66.85	128.5	
No	Team 1	4.25	59.25	94.5	6.4	65.25	125.5	
Yes	Team 1	5.95	68.25	115.5	6.2	70.2	118.5	
No	Team 1	7.5	79.65	114.5	9.2	82	127.5	
Yes	Team 1	5.4	60.65	111.5				
Yes	Team 1	6.05	67.35	111.5	7.2	70	129	
Yes	Team 1	5.15	63.55	102.5	7.55	70.7	126.5	
Yes	Team 1	3.95	56.3	94	6.3	61.95	126.5	
Yes	Team 1	5.6	64.3	112	7	70.5	121	
Yes	Team 1	4.8	61.45	110	7.1	64.05	141.5	
Yes	Team 1	4.9	63.25	108	6.1	66	128.5	
Yes	Team 1	5.4	64.95	107.5	6.85	66.55	130.5	
No	Team 1	4.8	63.05	105	4.75	63.25	110.5	
No	Team 1	5.2	63.45	110	5.45	64.85	109.5	
Yes	Team 1	4.3	60.45	106.5				
Yes	Team 1	5.3	60.9	110	6.75	65	125.5	523.736
No	Team 1	7.05	73.55	110.5	8.15	74.95	128	
Yes	Team 1	4.85	61.9	107	5.8	62.45	126.5	145.131
Yes	Team 1	6.65	73.95	111	8.2	74.75	128.5	
Yes	Team 1	4.7	62.25	103.5	6.55	64.95	125.5	

Yes	Team 1	5.6	64.7	113	5.8	67.25	118.5	
Yes	Team 1	6.05	67.35	112.5	7.75	71.7	134	187.276
No	Team 1	5.45	66.25	110.5	6.65	71	120	
Yes	Team 1	5.75	65.75	115.5	7.5	71.35	129.5	126.719
No	Team 1	6.6	70.75	115.5	7.1	71.85	122.5	
No	Team 1	5.2	61.25	112.5	5.85	63.45	120.5	
Yes	Team 1	5.1	59.95	110.5	5	61.45	108.5	
Yes	Team 1	6.95	72.35	110.5	8.65	73.55	131.5	277.561
Yes	Team 1	7.6	77	121.5	7.85	78.3	121.5	308.134
Yes	Team 1	5.35	63.1	116.5	6.55	64.65	130.5	
Yes	Team 1	5.6	66.05	120	7	70.9	126.5	82.645
Yes	Team 1	5	65.45	114.5	6.45	67	129.5	198.567
Yes	Team 1	5.55	62	114.5	6.65	67.8	121	90.286
Yes	Team 1	6.5	68.35	114.5	7.95	70.7	135.5	91.728
No	Team 1	7.2	75.5	110	8.65	76.7	127.5	210.896
Yes	Team 1	5.2	61.5	110	6.55	65	129.5	
Yes	Team 1	6.2	66.55	114.5	6.7	68.55	125	146.686
Yes	Team 1	7.35	71.45	113.5	7.4	74.15	117.5	197.028
Yes	Team 1	5.6	63.9	113.5	6.35	64.85	126.5	204.109
Yes	Team 1	5.7	64	114.5	6.5	66.4	125.5	157.253
No	Team 1	5.75	69.45	105.5	7.8	73.5	126.5	331.164
Yes	Team 1	7.9	83.5	110.5	9.5	84.9	125	
No	Team 1	7.25	73	114	8	72.75	130.5	233.502
Yes	Team 1	5.65	63.45	114.5	6.65	64.9	133.5	122.849
Yes	Team 1	5.9	66.5	116.5	7.2	68.8	131	195.999
Yes	Team 1	6.35	70.45	117	7.7	74.95	125	111.397
Yes	Team 1	7.15	74.4	127.5	8.4	74.85	140.5	88.42
Yes	Team 1	6.3	69.95	113.5	7.5	72.45	131	
No	Team 1	6.7	71	114	7.9	72.3	129.5	
No	Team 1	5.75	68.45	110.5	6.2	69.55	115.5	204.024
Yes	Team 1	4.05	57.25	95.5	5.7	61	125	462.618
Yes	Team 1	6.4	68.85	114.5	7.65	70.65	125.5	121.928
Yes	Team 1	5.5	65.05	112.5	6.55	66.05	131	110.129
Yes	Team 1	5.4	63.5	111.5	6.35	65.5	125.5	132.5
Yes	Team 1	5.65	68	111.5	7.45	73.45	126	363.843
Yes	Team 1	5.65	64.05	113.5	6.15	66	117.5	108.863
Yes	Team 1	6.4	70	109.5	8.3	72	134.5	111.12
Yes	Team 1	7.05	74.45	114	9.1	76.05	133.5	93.771
No	Team 1	5	61	114	5.75	64.15	120.5	
No	Team 1	10.4	93.15	126	11.7	96.2	136	240.553
Yes	Team 1	8.3	82.45	118.5	9.4	81.75	130.5	276.616
Yes	Team 1	7	73.45	113.5	7.6	74.5	125	257.105
Yes	Team 1	6.05	68.25	115.5	7.9	72	132.5	551.357
Yes	Team 1	9.1	89.05	125.5	11	91.5	140	
Yes	Team 1	5.95	66.95	112.5	7.25	69.5	130	115.57
Yes	Team 1	6.2	67.95	113.5	6.85	69	125	183.747
Yes	Team 1	6.95	72	122.5	7.9	76.2	127	261.257
Yes	Team 1	5.2	60.75	112.5	6.3	66	125	497.141
Yes	Team 1	3.9	56.85	96.5	3.7	57.25	89.5	
Yes	Team 1	6.1	64.65	112	6.85	65.55	125.5	261.332
Yes	Team 1	4.6	61	103.5	6.6	66	125.5	228.993

Yes	Team 1	7.5	76	117.5	8.2	77.7	125	92.386
No	Team 1	5.55	64.35	117	6.4	66.2	122.5	
Yes	Team 1	5.8	65.95	117.5	6.7	67.6	129.5	
No	Team 1	9	86.7	123	10.2	88.65	137.5	196.303
Yes	Team 1	5.2	64	111.5	6.35	68.5	110	90.529
No	Team 1	5.2	63.3	112.5	5.4	63.5	117.5	
Yes	Team 1	5.55	60.05	114.5	6.4	63.85	126.5	148.438
Yes	Team 1	4.95	60.85	104.5	6.8	64.35	125	198.589
Yes	Team 1	6.2	68.2	113.5	7.25	68.75	128	93.184
Yes	Team 1	5.55	63.65	114.5	6.5	66	125.5	279.397
Yes	Team 1	4.7	58.75	111.5	6	63.2	125	215.537
Yes	Team 1	7.6	73.95	114	8.7	75.45	125.5	368.545
Yes	Team 1	5.75	65.25	113.5	5.6	65.65	113.5	174.768
Yes	Team 1	5.5	61.35	112	5.55	63	115	267.39
Yes	Team 1	5.2	61.4	106.5	6.65	65.5	126.5	167.988
Yes	Team 1	7.2	73.1	120	8.55	74.65	130	108.267
Yes	Team 1	5.6	67.15	111.5	6.8	68.25	130	
Yes	Team 1	6.6	71.85	119	8.85	73.15	155	
Yes	Team 1	7	72.25	110.5	8.6	73.45	134.5	63.032
Yes	Team 1	4.8	60.45	110.5				
Yes	Team 1	4.6	60.5	110	6.2	65.1	125	
Yes	Team 1	5.65	64.35	114.5	6.6	67	125	391.73
Yes	Team 1	5.55	67.4	114.5	7	70.15	130	73.69
Yes	Team 1	5.75	66.25	110.5	6.45	68.8	120	
Yes	Team 1	5.8	64.3	113.5	6.75	65.95	125.5	219.531
Yes	Team 1	7.2	74.15	116.5	8.35	75	130.5	126.359
Yes	Team 1	6	67.3	116.5	7.5	69	134	
Yes	Team 1	6.4	67.5	113	7.65	72.75	126.5	
Yes	Team 1	6.7	71.5	114	8.1	75.55	125.5	
No	Team 1	5.75	66.5	112	6.75	68.95	120.5	
Yes	Team 1	7.2	73.2	114	8.3	76.05	128.5	
Yes	Team 1	5.5	65.5	116	6.5	70.95	119.5	
Yes	Team 1	6.1	66.75	113.5	7.35	72.35	125.5	
Yes	Team 1	6.85	72.65	120	7.85	72.8	131.5	
Yes	Team 1	7.25	73.5	113	9.05	74.3	137	
Yes	Team 1	7.1	72.55	123.5	7.6	73.95	128.5	
Yes	Team 1	5.6	66.25	120.5	6.85	66.55	127.5	
Yes	Team 1	6.55	69.45	124.5	7.65	70.8	141	
Yes	Team 1	5.05	63.15	111	7.05	69	131.5	
No	Team 1	7.7	79.45	112.5	8.1	82	114	
Yes	Team 1	5.9	64.45	111	6.85	69	120	
Yes	Team 1	6.9	71	114.5	7.95	72.95	125	137.364
Yes	Team 1	6.75	71.65	114.5	8.25	73	135	99.114
No	Team 1	5.55	63.3	113.5	6.5	67.35	123.5	
Yes	Team 1	5.1	62.75	113.5	6.5	66.15	127.5	83.426
No	Team 1	5.2	62.35	109	5.5	62.7	113	
Yes	Team 1	5.8	68.8	105	7.5	70.65	127.5	639.597
Yes	Team 1	6.05	66.45	113.5	7.7	71.4	130	
No	Team 1	6.05	68.55	106	7.8	70.85	129.5	92.037
Yes	Team 1	5.85	65.25	112.5	7.1	67.1	130.5	119.643
No	Team 1	9.5	88.55	114.5	10.9	88.85	125.5	655.942

No	Team 1	6.4	70.35	103.5	8.9	72.7	132	
No	Team 1	9.95	83.45	114.5	10.5	84.15	126.5	128.552
Yes	Team 1	5.4	64.45	114.5	6.45	65.6	127	
Yes	Team 1	5.05	66.5	113.5	6.7	67.2	127	404.648
No	Team 1	4.7	62.25	105.5	5.9	65.5	120	
Yes	Team 1	5.95	68	111.5	7.2	67.95	126.5	270.919
No	Team 1	10.5	96.5	118	12.2	98.7	133	
No	Team 1	8.8	86.6	120.5	8.1	86.65	119.5	
Yes	Team 1	6.2	69	113.5	6.85	71.85	122.5	79.92
Yes	Team 1	6.2	66.5	112.5	5.85	68	109.5	105.913
Yes	Team 1	5.35	64.1	110.5	6.5	65	125.5	242.717
Yes	Team 1	4.9	58.3	112.5	5.75	63.6	119.5	139.919
Yes	Team 1	5.6	67.6	112	5.9	68.65	111.5	261.346
Yes	Team 1	6	65.45	112	7.6	66.85	132	172.895
Yes	Team 1	5.8	65.7	114.5	7.1	70	120.5	306.988
No	Team 1	8	78.45	113.5	9.1	80.2	128	
Yes	Team 1	5.45	63.65	114.5	6.6	65.25	127.5	
Yes	Team 1	6.6	70.15	115.5	7.5	73.5	122.5	257.232
Yes	Team 1	6.1	67.85	120.5	7.6	71.45	130.5	99.596
Yes	Team 1	6.75	71.85	115	8.1	75	129	274.382
Yes	Team 1	5.5	64.45	112.5	6.5	66.3	130	
No	Team 1	7.6	75.5	110	8.1	76.8	122.5	
Yes	Team 1	6.45	71.5	114.5	7.75	72.45	130.5	
Yes	Team 1	5.35	61	114.5	6.3	65.25	125	
Yes	Team 1	6	66.65	116.5	6.95	67	131.5	
No	Team 1	8.75	85.65	119.5	11.1	85.3	141.5	114.998
Yes	Team 1	5.6	64.55	113.5	6.6	66.25	129.5	101.01
Yes	Team 1	4.95	63.35	105.5	6.35	68.1	115	
Yes	Team 1	6.3	68.45	120	6.9	70.75	119.5	270.749
Yes	Team 1	5.25	64.65	105	6.65	65.25	126.5	
No	Team 1	11.5	101.7	113.5	14	103.3	135.5	811.391
Yes	Team 1	5.5	63.15	111	6.75	67	125.5	110.155
No	Team 1	7.9	77.75	116	8.9	78	128	
No	Team 1	7.9	80.5	120	8.8	81	127	
No	Team 1	8.6	85.2	108	11.2	85.8	137	
Yes	Team 1	4.7	63.7	104	7	69.25	133	
Yes	Team 1	5.1	62	104	5.75	66.6	105.5	
Yes	Team 1	6	67.4	112	7.1	71	127	
No	Team 1	6.5	69	114	7.35	70.6	127	
Yes	Team 1	4.2	57	100	6.1	62.9	125.5	
No	Team 1	7.1	72.5	114	8.5	78	125	
No	Team 1	9.1	84.2	111.5	9.1	84.05	124	
Yes	Team 1	5.4	65	110	6.25	68.95	119.5	
No	Team 1	4.8	60.65	107.5	4.7	61.15	106	
No	Team 1	5.5	61.6	111.5	6.05	62.5	121	
Yes	Team 1	5.35	65.25	106.5	6.8	66.65	130.5	
Yes	Team 1	5.2	64.9	106.5	7.25	69.3	128.5	
Yes	Team 1	5.85	66.85	111.5	7.2	68.25	130.5	
Yes	Team 1	7.5	75.7	111.5	8.5	77.3	125.5	92.885
Yes	Team 1	5.7	65.65	113.5	6.95	69.5	117.5	
No	Team 1	6.4	69.25	117.5	6.45	69.45	116.5	

Yes	Team 1	5.2	62.85	113.5	5.95	64.8	129.5	
Yes	Team 1	5.7	63.15	114	6.2	64.05	125.5	197.835
Yes	Team 1	5.95	67.15	110.5	7.75	72.15	128.5	198.928
Yes	Team 1	8.1	79.5	116.5	9.5	83.5	131.5	227.862
Yes	Team 1	5.2	65.35	108.5	6.5	67	126.5	
No	Team 1	7.6	77.45	115.5	9.5	79.05	134.5	
Yes	Team 1	6.35	71.5	111	6.75	73.65	112.5	
Yes	Team 1	7.3	74.35	120.5	8.65	76	134.5	
Yes	Team 1	6.45	69.25	114	7.7	72.55	132.5	118.509
Yes	Team 1	6.1	67.3	112.5	7	70.95	118.5	208.773
Yes	Team 1	4.9	65.45	97.5	7.25	68.6	134.5	110.36
Yes	Team 1	4.8	60.9	110.5	6.1	66.5	120	258.617
Yes	Team 1	6.15	65.25	114.5	7.7	69	130	79.1575
No	Team 1	5.35	62.85	113.5				
Yes	Team 1	5.7	68.45	109.5	7.45	70.8	131	394.887
Yes	Team 1	4.8	62.95	97.5	5.35	65.75	102	144.313
Yes	Team 1	5.15	61.6	112	6.35	66.55	125.5	147.567
No	Team 1	5.4	65.65	108.5	5.4	65.9	110	
Yes	Team 1	5.65	67.45	116	6.6	70	120	331.466
No	Team 1	6.8	72	118	7.05	73	121.5	
Yes	Team 1	5.4	64.95	110.5	5.75	68.5	111	249.6
Yes	Team 1	6.15	67.15	111	7	69.85	125	124.896
Yes	Team 1	5.2	62.9	111.5	6.05	66.75	116.5	118.561
Yes	Team 1	5.45	60.75	109.5	6.7	66.35	125	136.951
Yes	Team 1	5.3	61.65	111	6.75	65.95	127	323.045
No	Team 1	5.25	60.5	113	6.15	65	120	
No	Team 1	10.3	91.3	123.5	10.9	94	133.5	338.385
No	Team 1	9.1	84.6	112.5	10.2	85.15	129	
No	Team 1	6.15	70.95	110	7.7	72.5	132.5	
Yes	Team 1	5.85	65.75	112.5	7.2	67.7	128	
Yes	Team 1	6.2	64.75	114	7	65.5	130.5	
No	Team 1	9.8	87	114.5	10.55	88	129.5	399.776
Yes	Team 1	6.65	70.45	120	7.6	72.25	131.5	93.897
Yes	Team 1	6.1	64.5	113.5	7.4	65.35	132.5	266.7
Yes	Team 1	5.6	64.5	112	6.65	65.5	127.5	
Yes	Team 1	5.25	63.45	112	6.4	64.5	125.5	148.734
Yes	Team 2	7.4	73.9	113.5	7.2	74.25	117	
Yes	Team 2	4.5	59.1	100	5.75	63.5	115.5	
Yes	Team 2	5.65	67.85	111.5	6.15	70.55	115.5	
Yes	Team 2	8.2	80.25	122.5	9.9	84.95	132.5	
Yes	Team 2	6.8	75.3	110.5	8.25	76.75	129.5	102.518
Yes	Team 2	6.6	72.25	122.5	7.65	74.45	131.5	
Yes	Team 2	5.95	69.15	105.5	7.3	73.65	125.5	
No	Team 2	7.75	76.65	120.5	8.9	78.55	133.5	
Yes	Team 2	7.45	74.95	113.5	8.65	78.65	125.5	280.217
Yes	Team 2	6.9	72.25	119	6.5	72.4	117	
Yes	Team 2	6.3	65.45	115.5	5.75	65.5	112.5	
No	Team 2	5.15	63.45	112.5	6.05	68.35	118.5	
Yes	Team 2	7.4	75.65	129.5	7.55	76.95	129.5	
Yes	Team 2	6.2	68	109.5	7.95	70.45	128.5	
No	Team 2	7.9	78.6	119.5	8.5	79.95	127	

Yes	Team 2	5.6	62.45	114.5	6.25	64.8	126.5	
No	Team 2	6.8	73.5	112.5	6.85	75.45	114.5	
Yes	Team 2	7	73.5	125.5	7.4	75.95	124	
Yes	Team 2	6.45	69.7	119.5	7.65	73.9	128.5	
Yes	Team 2	5.7	66	114.5	6.5	69.35	125	386.307
Yes	Team 2	6.7	72.5	118.5	7.5	74.8	128	
Yes	Team 2	5.9	67.2	115.5	6.8	67.65	125.5	
Yes	Team 2	7.7	76.65	123.5	8.9	77.6	143.5	
No	Team 2	6.35	72.35	104.5	7.55	73.25	125	109.662
Yes	Team 2	4.75	61.25	107.5	5.8	62.65	126.5	211.957
No	Team 2	4.7	61.95	108.5	6.05	63.35	129.5	358.882
No	Team 2	6.2	68.7	116.5	7.3	72.25	129	
Yes	Team 2	4.15	60.65	88.5	4.05	61	85.5	
Yes	Team 2	5.85	65.65	110.5	7.1	69.25	126	117.541
No	Team 2	7.05	76.25	112.5	7.55	78.2	118.5	
Yes	Team 2	5.95	67.85	118	7.2	70.45	126.5	
No	Team 2	7.75	77.55	125.5	9	80.95	134.5	
No	Team 2	8.5	80.8	114.5	9.8	82.35	129.5	103.83
Yes	Team 2	6	66.95	109.5	7.65	71	126.5	173.258
Yes	Team 2	6.15	69.6	117.5	7.7	74.7	129	165.134
No	Team 2	8.6	83.1	120.5	9.3	84.45	130.5	
No	Team 2	9.6	88.6	125.5	10.7	89.9	139.5	
Yes	Team 2	5.15	63.5	112.5	5.3	64.35	110	302.124
No	Team 2	8.7	83.15	111.5	10	83.85	130.5	192.591
Yes	Team 2	6.55	69.3	125.5	6.75	72.3	124.5	
Yes	Team 2	5.9	67.75	111.5	7.2	71.95	127.5	
No	Team 2	11	98.2	132	12.8	101.85	144.5	
Yes	Team 2	6.25	68.65	115.5	7.6	69.45	134.5	53.439
No	Team 2	6.6	77.2	109.5	9.1	79.45	134	361.488
Yes	Team 2	5.45	62	112	5.7	64.2	114.5	305.338
No	Team 2	12	100.95	128.5	11.8	102	129.5	614.257
Yes	Team 2	6.3	71.3	114.5	7.3	74.75	126.5	225.899
Yes	Team 2	6.1	68.1	114.5	6.85	70.25	129	
Yes	Team 2	5.6	65.2	114.5				
No	Team 2	6.9	75.35	121.5	7.9	78.95	129.5	
Yes	Team 2	8.2	79.15	109.5	8.6	79.85	120.5	
Yes	Team 2	6.55	67.25	113.5	7.85	69.95	133.5	107.14
Yes	Team 2	5.1	65.4	120	6.65	66.95	126.5	151.042
No	Team 2	8.2	83.45	117	8.3	84.65	123.5	
Yes	Team 2	6.5	69.15	125.5	6.15	70.65	117.5	341.813
No	Team 2	6.05	69.35	113.5	6.15	69.95	115.5	
Yes	Team 2	5.75	66.95	115.5	6.2	70.5	118.5	295.787
No	Team 2	8.15	82.85	120.5	9.1	84.15	130.5	
No	Team 2	5.2	61.85	112.5	6.2	63.35	125	
No	Team 2	8.75	84.15	129.5	8.6	84.65	129.5	
No	Team 2	5.5	66.45	114.5	6.35	69.5	124.5	
Yes	Team 2	6.5	69.95	128.5	7.8	72.15	139.5	155.365
Yes	Team 2	4.7	63.45	107.5	6.85	67	132.5	105
Yes	Team 2	5.35	62.45	112.5	6.3	63.35	128.5	134.388
Yes	Team 2	6.45	69.45	108.5	7.15	73.45	116.5	112.45
Yes	Team 2	5.2	61.25	114.5	5.9	63.65	118.5	133.08

Yes	Team 2	5.5	65.65	115.5	6.75	66.65	133.5	132.148
No	Team 2	7.05	72.95	133.5	7.55	73.5	136.5	
No	Team 2	6.25	71.45	119.5	6.3	72.05	119	
Yes	Team 2	4.75	61.95	113	4.8	62.2	116.5	
Yes	Team 2	7.5	80.15	116.5	9.1	83.45	127.5	
No	Team 2	6.15	74.05	92.5	7.9	75.7	118.5	
Yes	Team 2	5.8	66.65	113	7.15	68.45	132	
No	Team 2	8.7	83.9	124	8.9	85.65	127.5	
Yes	Team 2	5.9	66.45	113	5.6	66.5	113	
Yes	Team 2	6.25	68.3	114	5.7	68.3	107.5	
Yes	Team 2	6.8	73.5	111.5	8.05	75.8	132.5	
No	Team 2	6	67.45	113.5	6.7	70.65	119.5	
Yes	Team 2	4.85	62.35	108.5	5.3	65.95	112.5	
Yes	Team 2	4.65	62.9	100.5	5.55	67.45	109.5	
Yes	Team 2	6.55	70.45	114.5	7.2	70.75	131.5	
No	Team 2	5.95	64.9	113.5	6.7	67.05	122.5	
Yes	Team 2	6	71.45	123.5	7.5	74	130.5	
Yes	Team 2	7.1	73.35	114.5	7.1	74.4	115.5	
Yes	Team 2	6.95	71.9	118	8.1	74.45	130.5	165.417
Yes	Team 2	5.95	66.25	119.5	6.25	69.05	120.5	
Yes	Team 2	6.3	70.15	118.5	6.55	71.75	123.5	
No	Team 2	7.3	74.75	124	7.8	74.95	125	
Yes	Team 2	5.05	59.8	112.5	5.35	62.2	116.5	
Yes	Team 2	5.75	64	113.5	7.1	69.45	126.5	259.513
Yes	Team 2	6.5	71.8	124.5	7.75	74.75	125.5	110.322
Yes	Team 2	4.55	56.65	109	5.45	61.35	114.5	
No	Team 2	6.85	72.95	117.5	9	74.95	142.5	275.596
Yes	Team 2	5.35	63.25	113.5	5.9	67.35	119.5	420.412
Yes	Team 2	6.45	68.45	113.5	7.4	72.95	125	
No	Team 2	7.35	74	114	8.45	75.7	125.5	141.334
Yes	Team 2	5.15	61.9	113.5	6.7	67.45	125.5	94.028
No	Team 2	6.55	69.45	122.5	7.8	70	139	
Yes	Team 2	5.55	65.45	116.5	6	69.45	115.5	
Yes	Team 2	5.7	65.1	115.5	6.65	66.65	128	209.754
Yes	Team 2	3.8	55.45	95.5	4.3	58	107	170.34
Yes	Team 2	6	68.25	114.5	7.6	71.65	130	154.52
Yes	Team 2	5	62	110.5	5.55	64.45	113.5	174.615
Yes	Team 2	6.25	67.7	127	7.3	72.95	137.5	248.373
Yes	Team 2	6.2	68.95	114.5	7.7	70.6	133.5	251.013
Yes	Team 2	5.25	65	103.5	6.25	68.95	114.5	124.896
Yes	Team 2	5.05	60.3	112.5	5.55	62.65	124.5	76.903
No	Team 2	6.9	73.75	113.5	8.6	74.95	137	133.076
Yes	Team 2	5.1	63.75	115	6.2	68	119	142.281
Yes	Team 2	7.65	76.85	126.5	9.05	80.25	133	189.646
Yes	Team 2	8.9	84.15	114.5	10.3	84.75	130.5	195.227
Yes	Team 2	3.85	56.95	115.5	5.4	60.65	128.5	143.759
Yes	Team 2	7.05	73.45	119.5	7.9	76.45	126.5	207.59
No	Team 2	10.6	99.35	114.5	10.9	99.8	120.5	
No	Team 2	5.15	64.2	110.5	6.2	68	123	
Yes	Team 2	6.7	73.75	114	7.3	76.55	120	
No	Team 2	7.55	77.95	109	9.4	79.95	126.5	453.981

Yes	Team 2	6.35	69.8	112.5	8.1	74.15	134	
Yes	Team 2	4.8	61.5	107.5	5.1	61.8	116.5	70.763
No	Team 2	7.35	75.65	113.5	8.3	77.45	121	
Yes	Team 2	5	65.1	104.5	6.7	67.05	126	
No	Team 2	5.7	65.7	108.5	6.6	67.15	120.5	
No	Team 2	6	67.9	113.5	6.8	71.45	121.5	
Yes	Team 2	6.2	67.35	113.5	7.65	70	129.5	
No	Team 2	6.15	67.3	111.5	6.9	71	120	
Yes	Team 2	7.3	76.85	110.5	9.55	78.65	135.5	
No	Team 2	6.9	73.05	106		74.95	123.5	
Yes	Team 2	8.1	76.5	113.5	9.3	77.75	128.5	231.912
Yes	Team 2	7	72.9	125	8.25	73.85	131.5	
Yes	Team 2	6.25	72.4	111.5	7.1	75.75	125.5	
Yes	Team 2	6.2	66.8	113.5	6.7	67.15	125	
Yes	Team 2	5.3	67.5	114.5	6.1	69.05	115.5	
Yes	Team 2	6.6	70.1	121	7.75	72.95	128.5	
No	Team 2	6.55	69.95	113.5	6.8	71.35	119.5	
No	Team 2	5.15	63.95	109.5	6.2	66.15	122.5	
No	Team 2	5.9	66.25	109.5				
Yes	Team 2	5.35	65.15	105.5				
No	Team 2	5.5	65	113.5	6.2	69.15	120.5	
Yes	Team 2	5.7	68.95	106.5	7.2	71.45	126.5	
Yes	Team 2	7	68.45	113.5	8.05	71.35	128.5	
Yes	Team 2	5.5	63.3	114.5	6.5	66.25	129.5	
Yes	Team 2	5.5	67	116	6.5	71.75	125.5	
Yes	Team 2	6	67.45	114.5	6.95	70.25	125.5	
Yes	Team 2	7	72.7	113	8.1	73.85	127.5	168.238
Yes	Team 2	6.2	65.5	113.5	7.2	67.9	127.5	
Yes	Team 2	6	65.25	114.5	7.05	68.2	128.5	242.824
No	Team 2	8.05	79.5	113.5	9	80.75	125.5	195.709
No	Team 2	8.9	83.2	114.5	9.9	85.45	131.5	
Yes	Team 2	5.35	66.1	112	7.25	67.95	134.5	
No	Team 2	10.3	94.95	124	10.9	96.45	128	
No	Team 2	7.35	77.95	100.5	9.3	77.95	131.5	
Yes	Team 2	5.2	60.95	103.5	6.3	64.95	125.5	119.98
Yes	Team 2	6.3	66.65	113.5	7.2	67	130.5	
No	Team 2	6.35	72.2	113.5	7.55	72.85	126.5	
No	Team 2	5.35	65.8	109.5	6.5	69.25	125.5	
No	Team 2	8.45	83.25	115.5		83.45	116.5	
Yes	Team 2	7.2	72.45	110.5	8.85	74.45	130.5	
No	Team 2	7.25	73.65	118.5	9.05	74.25	131.5	
No	Team 2	8.3	80.65	120.5	9.2	81.35	133.5	
No	Team 2	8.05	78.65	114	8.9	79.65	127.5	
No	Team 2	9.5	92.8	118.5	10.9	93.3	129	
No	Team 2	7.3	75.45	114.5	9	79.5	130.5	147.699
No	Team 2	6.95	76.65	105.5	8.7	77.25	130.5	
Yes	Team 2	5.4	66.4	110.5	7.35	69.45	131.5	66.986
No	Team 2	7.8	80.9	119.5	8.4	82.15	127	
Yes	Team 2	4.2	58.8	109.5	5.55	66	115	
Yes	Team 2	5.1	64.95	105.5	6.8	70.95	122.5	
Yes	Team 2	5	62.45	106.5	5.2	63.5	114.5	

No	Team 2	6	66.05	111.5	6.2	67.2	116.5	
Yes	Team 2	5.45	64.25	110.5	5	64.9	105.5	
No	Team 2	6.5	69.8	110.5	6.45	69.85	115.5	
Yes	Team 2	6.4	71.45	113	8.4	75.85	132.5	211.576
Yes	Team 2	5.8	66.95	116.5	7.25	68.25	129	164.459
Yes	Team 2	5.35	62.45	111.5	7.2	65.7	132.5	226.451
No	Team 2	8.3	83.45	117.5	9.3	87.25	125.5	690.517
No	Team 2	5.4	62.5	100.5	7.4	65.85	132.5	327.48
Yes	Team 2	6.7	72.95	119.5	8.45	75.25	138.5	
Yes	Team 2	5.35	63.45	114.5	6.45	67.65	121.5	168.887
Yes	Team 2	5.75	67	107.5	7.4	68.75	127.5	32.324
Yes	Team 2	4.6	61	109.5	5.15	63.6	113.5	
Yes	Team 2	6.2	68.25	115.5	7.7	71.35	126.5	
Yes	Team 2	5.25	64.25	107.5	7	67.95	131.5	135.008
Yes	Team 2	5.95	70.95	114.5	7.5	72.25	125.5	
Yes	Team 2	6.05	65.95	112.5	7.6	66.85	130.5	
No	Team 2	7.6	77.4	110.5	7.15	78.1	110	
Yes	Team 2	7.15	71.9	113.5	8.4	73.15	132.5	55.149
Yes	Team 2	6.35	69.65	111.5	6.3	70.35	117.5	328.094
Yes	Team 2	6.55	72.95	122.5	7.8	73.5	129.5	401.457
Yes	Team 2	5.35	67.95	105	8.1	69.85	136.5	94.888
Yes	Team 2	6.8	72.65	110.5	8.4	73.95	129.5	61.1375
Yes	Team 2	5.85	62.75	114.5	7.2	65.45	131.5	60.479
Yes	Team 2	6.4	69.95	109.5	7.6	71.45	128.5	115.398
No	Team 2	6.45	68.45	110.5	7.75	70.45	131.5	
Yes	Team 2	5.65	67.85	105.5	7.65	70.2	133.5	
No	Team 2	6.9	75.1	120.5	8	78.7	125.5	99.114
No	Team 2	7	75.65	110.5	8.2	76.65	127.5	262.104
Yes	Team 2	5.65	63.45	113.5	6.6	66.4	126.5	155.709
Yes	Team 2	5.05	63.15	107.5	7.25	68.95	128	55.708
Yes	Team 2	6.6	72.45	118.5	7.5	73.45	130.5	
Yes	Team 2	5.8	63.95	114.5	6.3	64.85	129	
Yes	Team 2	4.45	57.25	105.5	4.7	58.95	107	
Yes	Team 2	5.45	63.3	105.5	6.9	68.45	126	206.383
Yes	Team 2	5.45	65.25	111.5	5.45	67.55	105.5	249.924
Yes	Team 2	6	65.25	112.5	7.1	68.35	125.5	361.331
No	Team 2	5.5	71.1	109.5	8.1	75.95	131.5	301.904
Yes	Team 2	5.05	61.9	110.5	6.7	63.95	130.5	
Yes	Team 2	5.15	62.45	118	6.15	67.4	124	319.249
Yes	Team 2	6.7	73.2	114.5	7.2	75.25	115	285.928
Yes	Team 2	5.4	62.45	110.5	5.45	65.1	112	131.387
Yes	Team 2	4.25	59.35	101.5	6.65	64.9	131.5	321.799
Yes	Team 2	5.8	65.5	112.5	6.9	67.75	132	148.789
Yes	Team 2	4.55	57.95	106.5	6	61.35	133.5	
Yes	Team 2	5.15	67.35	98.5	7	68.9	129.5	309.338
Yes	Team 2	5.2	64.25	112.5	6.4	67.25	127	207.703
Yes	Team 2	4.4	59.45	95.5	6.6	63.85	130.5	60.26
Yes	Team 2	6.4	69	117.5	7.65	71.95	125.5	309.452
Yes	Team 2	3.45	56.45	75.5	3.8	58.35	91.5	
Yes	Team 2	6.1	68.65	114.5	7.6	72.35	125.5	208.232
Yes	Team 2	6.5	72.25	107.5	8.45	74.15	129.5	342.755

Yes	Team 2	5.85	66.3	110	6	67.45	114.5	
Yes	Team 2	6.55	70.85	112.5	7.7	70.95	129.5	139.331
Yes	Team 2	4.8	60.45	100.5	7.4	66.25	135	188.592
Yes	Team 2	6.95	69.4	113.5	7.85	70.7	127	228.412
Yes	Team 2	6.1	67.35	114.5	7.3	68.45	130.5	524.426
No	Team 2	7.55	76.9	109.5	9.2	77.35	131.5	442.209
Yes	Team 2	4.95	60	112.5	6.05	61.25	128.5	159.734
Yes	Team 2	5.8	66	114.5	7.1	69.25	129.5	248.938
Yes	Team 2	5.8	66.35	113.5	7.1	70	127	167.46
Yes	Team 2	5.55	63.25	114.5	6.65	65.65	126	87.035
Yes	Team 2	4.45	60.2	112.5	6.2	63.65	125.5	182.635
Yes	Team 2	5.5	63.95	112.5	6	69.8	112.5	130.752
No	Team 2	8.2	79.6	109.5	8.35	80	116.5	202.179
Yes	Team 2	7.2	73.25	113.5	8.8	75.6	143.5	440.499
Yes	Team 2	5.05	62.1	103.5	6.3	67.7	121.5	374.338
Yes	Team 2	5.05	61.4	99.5	5.45	63.65	103	215.172
Yes	Team 2	6.8	71.35	115.5	8	72.4	130	160.853
Yes	Team 2	5.5	64.95	113.5	7.05	70.45	126	79.466
Yes	Team 2	6.9	73.45	102.5	8.65	74.95	126	287.594
No	Team 2	7.75	76.95	113.5	9.5	78.45	132.5	229.884
Yes	Team 2	6.05	66.05	114.5	6.7	66.35	128.5	243.85
Yes	Team 2	6.55	68.1	114.5	7.15	69.45	129.5	241.137
No	Team 2	5.95	65.8	114.5	6.7	68.25	119.5	
Yes	Team 2	5.85	63.9	114.5	6.6	68.5	125	168.907
No	Team 2	6.5	69.4	116	7.3	73.65	117.5	
Yes	Team 2	5.65	64.65	114	6.7	67	126.5	188.307
No	Team 2	7.85	79.95	119.5	8.2	80.55	126.5	
No	Team 2	4.4	58.25	106	4.6	58.45	107.5	
Yes	Team 2	6.2	67.7	109.5	7.6	69.35	129.5	264.347
No	Team 2	6	65.75	107.5	6.1	67.15	111.5	
No	Team 2	5.6	64.95	106.5	6	66.85	117.5	112.692
Yes	Team 2	4.75	62.45	102.5	5.95	66.95	117.5	130.673
Yes	Team 2	6.2	68.75	116.5	7.7	69.75	137.5	138.44
Yes	Team 2	4.4	59.15	110.5	5.95	63.95	126.5	162.895
Yes	Team 2	5.45	65.15	113.5	6.85	68.25	126	
Yes	Team 2	5.1	64.15	111.5	6.45	66.95	127.5	265.858
Yes	Team 2	6.3	71.35	106.5	7.6	72.5	125.5	92.327
Yes	Team 2	5.1	61.65	111.5	6.25	62.95	127.5	303.199
Yes	Team 2	6.3	71.15	111.5	6	72.45	114.5	
Yes	Team 2	6.15	67.25	120	7.05	68.85	126.5	217.632
Yes	Team 2	7.15	73.45	120.5	8.25	75.25	131.5	167.023
No	Team 2	5.25	62.65	120.5	5.8	63.75	125.5	
Yes	Team 2	5.55	65.15	113.5	6.95	67.75	126.5	246.163
Yes	Team 2	5.15	62.95	114.5	5.6	65.2	114.5	
Yes	Team 2	5.85	67.25	116.5	6.85	68.45	132.5	221.727
Yes	Team 2	5.85	69.25	106.5	7.1	70.25	127.5	34.105
Yes	Team 2	5.45	67.65	106.5	6.7	69.95	126.5	481.828
Yes	Team 2	5.5	64.25	114.5	6.6	65.45	127.5	40.643
No	Team 2	8.4	82.2	126.5	9	82.85	134.5	188.592
Yes	Team 2	4.5	59	103.5	5.3	62.15	109.5	
Yes	Team 2	4.9	60.35	106.5	6	65.1	116	

Yes	Team 2	6.3	70.85	113.5	7.3	72.45	128.5	81.725
Yes	Team 2	6.75	72	106.5	8.15	73.5	127.5	
Yes	Team 2	5.45	63.85	109	6.65	67.65	116	
Yes	Team 2	6.35	72.55	114.5				
Yes	Team 2	6.6	70.35	115.5	7.85	72.85	130	
No	Team 2	9.8	89.1	117				
Yes	Team 2	6.45	71.05	122	7.4	74.95	130.5	
Yes	Team 2	6.05	66.15	113.5	5.8	67.55	116	
No	Team 2	6.2	75.2	99	9.3	77.45	128	
Yes	Team 2	5.7	67.2	109.5	6	70.25	109.5	
No	Team 2	6.55	70.35	110	7.4	71.35	121.5	
Yes	Team 2	5.45	65.95	112.5	7	69.95	126.5	
Yes	Team 2	5.9	65.65	113.5	7.5	68.45	129.5	
Yes	Team 2	5.05	63.95	99.5	6.8	65.85	131.5	
Yes	Team 2	4.65	61.6	109.5	5.3	65.75	120.5	
Yes	Team 2	6.9	71.25	110.5	8.2	74.95	128.5	40.084
No	Team 2	5.45	65.45	109.5	6.65	67.75	124.5	
No	Team 2	6.15	70.2	115.5	6.65	73.45	114.5	
Yes	Team 2	7.8	81.05	116.5	8.6	83.35	122.5	
No	Team 2	9	85.15	114.5	9.7	86.7	128	181.878
Yes	Team 2	5.4	65.1	103.5	5.15	65.45	95.5	
Yes	Team 2	6.5	71.45	114	8.25	74.45	132.5	165.7
Yes	Team 2	4.9	62.75	109.5	7.1	68.5	127.5	
No	Team 2	9.2	85.75	116.5	9.7	87.05	122.5	
Yes	Team 2	7.1	73.55	122.5	8.25	74.95	130.5	163.417
Yes	Team 2	8.2	81	114.5	9.3	82.65	133.5	487.866
Yes	Team 2	6.2	73.8	106.5	5.85	73.15	98	
No	Team 2	5.85	63.6	114.5	5.5	64.55	105.5	
No	Team 2	7.4	78.45	112.5	8.35	78.65	124.5	
Yes	Team 2	5.35	63.65	113.5	7.2	68.35	126.5	207.27
Yes	Team 2	6.65	72.35	121.5	7.75	74.25	129.5	412.329
Yes	Team 2	6.65	70.3	116.5	7.8	71.85	129.5	240.998
Yes	Team 2	5.15	60.4	112.5	6	62.2	130.5	266.526
Yes	Team 2	5.1	61.1	112.5	6.2	66.95	119	
Yes	Team 2	7.35	76.95	118.5	8.9	79.6	140.5	74.159
Yes	Team 2	6	70.75	105.5	7.9	72.65	132	
Yes	Team 2	3.75	56.45	92.5	4.85	60.85	109.5	111.986
No	Team 2	7.8	83.05	114.5	10	84.85	134.5	323.061
Yes	Team 2	5.8	65.8	112	7	68.2	129.5	80.452
Yes	Team 2	5.4	65.45	114.5	6.05	68.6	117	
Yes	Team 2	5.4	64.7	113	5.85	66.3	117.5	179.695
No	Team 2	8.35	80.4	113.5	9.1	82.25	126.5	397.185
No	Team 2	6.3	69.25	116.5	6.9	70	123.5	
No	Team 2	5.55	65.45	106.5	6.15	66.8	118.5	234.606
No	Team 2	6	67.5	109.5	7.7	70.3	126.5	253.78
No	Team 2	8.6	82.6	123.5				
No	Team 2	7.1	74.55	105.5	8.3	76.25	129.5	292.091
Yes	Team 2	7.45	78.25	115.5	8.6	80.2	126.5	266.16
Yes	Team 2	6.2	72.15	104.5	7.15	74.2	120.5	112.395
Yes	Team 2	6.6	71.15	114.5	7.4	73.95	127.5	159.446
Yes	Team 2	6.6	74.55	111.5	9	77.95	140.5	141.7

Yes	Team 2	4.95	63.95	109.5	4.9	64.55	102.5	
Yes	Team 2	4.65	60.65	111.5	5.15	62.45	118.5	163.183
Yes	Team 2	6.55	69.75	116	7.35	73.45	123.5	
Yes	Team 2	6.2	67.9	115.5	6.3	69.25	116.5	
Yes	Team 2	4.9	62.4	105.5	5.85	65.45	119.5	
No	Team 2	7.1	78.65	106.5	7.7	79.95	116.5	
Yes	Team 2	5.45	60.55	111.5	6	64.95	117.5	
No	Team 2	6.85	73.85	115.5	7.1	74.7	119.5	
Yes	Team 2	7.9	79.5	114.5	8.9	81.55	127.5	48.263
Yes	Team 2	6.5	70.25	114.5	7.45	72.25	126.5	214.7
No	Team 2	5.5	64.45	115	6.05	67.15	125.5	
Yes	Team 2	5.9	68	119.5	7.2	71.95	126.5	
Yes	Team 2	5.1	61.35	102	7	66.25	125.5	
No	Team 2	13	105.25	132.5	14.3	107.75	136.5	236.239
No	Team 2	7.65	76.9	124.5	8.3	80	126.5	
No	Team 2	10.2	91.15	127.5	11.6	94.25	136	
No	Team 2	11.95	99.35	123.5	13.4	101.5	142.5	
Yes	Team 2	6.85	73.65	116.5	8.5	76.7	130	223.13
Yes	Team 2	7.05	76.45	115.5	8.7	78.95	135.5	362.931
No	Team 2	10.15	90	123.5	11.6	90.75	140	164.325
No	Team 2	5.95	69.3	117.5	7	72.3	131	
Yes	Team 2	5.1	62.15	109.5	6.7	65.6	128.5	113.989
Yes	Team 2	6.05	71.25	98.5	8.8	73.95	127.5	344.711
Yes	Team 2	5.45	65.25	113.5	6.7	67.25	126.5	64.939
Yes	Team 2	8.3	82.4	123.5	9.8	83.85	136	217.643
Yes	Team 2	6.3	69	125	7.7	71.25	138.5	106.635
Yes	Team 2	7.45	79.25	124.5	9.3	81.25	144.5	232.077
Yes	Team 2	8.2	80.35	121.5	9.2	81.5	130	
Yes	Team 2	5.05	62.15	113.5	6.25	64.75	126.5	232.089
No	Team 2	8.1	89	100.5	10.9	89.45	127.5	552.173
No	Team 2	5.4	61.45	112.5	6.25	66.45	120.5	
No	Team 2	6.65	76.45	114.5	8.4	79.3	132.5	258.295
No	Team 2	7.4	76.25	120.5	8.9	78	135.5	352.316
No	Team 2	7.45	76.25	118.5	7.45	76.9	120.5	199.471
Yes	Team 2	5.6	63	114.5	5.7	64.65	115.5	
Yes	Team 2	6.8	72.25	124.5	7.85	75.3	131	445.838
Yes	Team 2	6.75	70.95	124.5	7.7	72.65	129.5	234.606

MMA_adm	tHcy_adm	Cob_exit	MMA_exit	tHcy_exit	cB12_adm	cB12_exit	Hb_adm	Hb_exit
							90	107
							118	121
							115	
0.889911	11.42564	311.722	0.325035	5.947304	-1.18904	0.07538	110	121
							76	109
							90	
							86	105
							89	90
							112	
							96	
							93	99
							105	
0.233126	11.07138	182.731	0.254497	4.972808	-0.85794	0.007288	103	112
							80	105
							124	122
							74	105
							133	
							111	113
							119	
							79	100
							61	108
0.868757	22.4373	434.652	0.264538	12.14513	-1.65292	-0.03279	104	105
0.132456	15.61601	125.052	0.333741	8.879874	-0.3782	-0.74358	95	113
							107	
							93	
0.653246	20.13358	226.933	0.925024	9.541394	-1.51298	-1.04195	104	98
0.88698	10.79692	307.691	0.565969	5.958084	-0.93251	-0.27441	85	88
0.103132	4.950909	205.148	0.365411	7.982265	0.542398	-0.43338	104	99
0.195228	10.86989	878.208	0.139816	7.654958	0.453336	1.089085	70	105
2.746852	11.42885	79.925	0.822467	11.72779	-2.28185	-1.71436	113	68
2.126239	12.66733	301.558	0.36552	7.483959	-1.94476	-0.15858	106	113
							98	
0.266612	3.12122	283.156	0.153706	5.479872	0.248902	0.530599	123	127
0.409673	6.485342	349.394	0.266243	5.501755	-0.24033	0.317428	124	132
0.352809	11.03391	102.424	0.369429	6.343096	-1.4076	-0.72227	109	115
0.249562	5.687534	377.979	0.164103	5.562446	0.511933	0.66052	117	117
0.62228	5.883401	281.653	0.673533	9.031591	-0.51496	-0.68739	112	117
1.123214	19.93166	192.434	0.200795	11.59306	-2.09275	-0.33478	115	99
0.188072	7.002545	345.716	0.307736	8.887445	0.013821	-0.07454	82	101
0.989171	14.21853	195.609	0.215574	5.924232	-1.83507	0.043645	99	112
0.11601	4.523817	532.373	0.110033	4.492019	1.503788	1.260497	86	105
0.262302	8.597777	276.345	0.348363	9.241295	-0.39751	-0.31177	100	108
							107	
							107	
							100	119
0.504673	8.90405	331.177	0.25113	6.268267	-0.41167	0.239674	96	121
0.84836	14.64516	217.197	0.231202	11.39035	-1.39655	-0.3362	123	
0.876808	16.60477	218.109	1.938673	19.46255	-1.3251	-1.92512	104	125
0.316113	13.81557	419.333	0.14569	8.810544	-0.38905	0.512771	112	124
0.585295	3.975483	365.341	0.371637	9.497327	-0.163	-0.19719	88	118

0.526593	24.22638	152.655	0.319764	9.670662	-1.526	-0.64856	108	108
2.284194	9.77773	138.78	0.690083	11.36008	-2.09197	-1.26511	67	101
0.133934	7.483195	121.467	0.184527	5.79596	-0.32827	-0.14029	60	104
							86	94
2.003216	20.32026	314.066	1.01782	11.2573	-1.95753	-1.00356	96	111
0.32139	9.2183	220.104	0.441935	8.43155	-0.51757	-0.53962	99	128
							107	91
0.239638	6.255549				0.067931	0.641407	133	
							89	39
							74	
1.189285	14.64628	284.708	1.154601	9.07816	-1.95378	-1.00901	77	101
0.332475	8.640601	196.291	0.542721	9.390574	-0.53528	-0.79874	116	114
							90	94
0.338289	7.070984	334.067	0.261957	8.640295	-0.05417	0.020825	99	112
							95	
							100	124
0.413181	5.83648	364.37	0.312087	4.74302	-0.06578	0.336952	86	101
							90	
0.496922	15.34501	300.406	0.290947	8.120013	-1.11001	-0.0709	113	111
0.248129	14.32868	255.855	0.529926	10.80792	-0.50509	-0.70908	96	105
0.300454	8.079816	293.919	0.191854	5.034932	0.06649	0.468565	117	111
1.265252	18.96177	287.531	1.124374	8.940816	-1.65526	-0.97799	104	109
0.236306	6.164511	427.66	0.25984	4.110171	0.239153	0.639613	102	107
0.265022	8.00791	144.048	0.269932	7.682889	-0.92997	-0.441	107	112
0.364608	13.53771	283.594	0.261278	8.131954	-0.72841	-0.04108	118	120
							115	
0.729901	12.18731	227.278	0.879043	11.51732	-1.25615	-1.12326	102	117
							86	
0.169288	9.639943	223.854	0.261632	6.933703	-0.10502	-0.08933	91	57
0.138812	6.059908	406.007	0.204529	6.10172	0.757068	0.510194	112	134
							97	
							126	126
							70	109
							104	
							111	
							72	
							111	
							68	
							106	89
							106	
							95	
1.175232	19.555	311.487	0.762134	17.05876	-2.37642	-1.08429	87	102
1.2784	11.84364	292.888	0.185994	8.017803	-1.41193	0.197133	102	122
							102	
0.254119	12.09769	110.812	0.8123	14.28572	-1.06788	-1.63089	100	105
							117	115
							87	
1.324627	13.5584	218.887	0.250474	5.783851	-1.61388	0.035246	87	110
							92	
							89	
0.204232	11.1543	221.345	0.216992	6.335046	-0.4384	0.07447	99	112

							63	99
0.531877	6.566862	258	0.870694	5.662825	-0.50262	-0.61324	93	120
0.464814	9.787626	198.928	0.395976	7.914467	-0.85589	-0.49586	103	107
							85	111
1.273255	24.04399	293.559	0.762369	12.79575	-2.33022	-0.94745	71	108
							119	
0.267891	13.75973	207.438	0.465258	9.040586	-0.71099	-0.64916	97	108
0.328319	9.573764	267.653	0.165308	6.026572	-0.57905	0.391237	114	110
0.795644	10.45749	315.129	0.24054	7.180717	-1.30075	0.151576	104	116
0.324003	10.09768				-0.31005	-0.35921	87	
							110	55
0.421604	11.59449	589.669	0.204635	7.695046	-0.43895	0.597804	98	102
							94	104
0.152651	13.85029				-0.28002	-0.47262	112	
0.576873	11.13765				-0.34332	0.030088	81	
							92	
							114	105
0.140418	6.228388	190.55	0.529659	11.74153	0.310623	-0.93661	122	126
0.604471	9.645433				-0.48336	-0.19888	155	
							109	
2.05943	27.71632	633.819	0.928869	11.89579	-2.0078	-0.55745	120	108
0.3767	8.44414	342.249	0.162539	6.199468	-0.52102		106	110
0.206339	13.26111				-0.67432	-0.4015	114	
							50	72
							87	124
							104	118
0.115483	8.2282				0.09422	0.777331	79	
							116	
1.037533	11.50029	110.597	0.436998	7.009334	-1.73257	-0.83771	112	102
0.503419	25.44195	322.941	0.377147	8.538849	-0.90061	-0.21662	108	96
							94	101
							96	95
0.120927	5.658392	242.025	0.126366	5.558122	0.734113	0.545992	88	99
							94	120
2.616853	22.01228	234.558	2.067708	11.50302	-2.73463	-1.61192	65	108
0.856492	5.67687	306.075	1.042541	5.725909	-0.44143	-0.62557	65	101
0.552999	23.45572	311.967	0.289441	4.701408	-1.33395	0.292863	106	115
							122	
							115	126
							101	
1.194742	12.41112	114.229	0.935121	11.40816	-1.63486	-1.56336	101	104
0.687918	22.18791	172.624	0.356089	9.125426	-1.68375	-0.60406	85	118
0.313444	6.596292	494.567	0.222437	7.726662	0.307792	0.433877	117	71
							105	
							105	
0.523858	12.60222	145.733	0.175644	8.404642	-1.5985	-0.22622	109	112
0.13616	5.972366	487.776	0.293694	5.082637	0.930178	0.512929	125	112
							92	107
							83	105
							98	
							99	104

							59	92
							107	
							101	115
							93	
							93	
							87	
							78	107
							69	101
							108	112
							123	85
							78	93
							78	
							98	
							78	
							68	
							78	
							105	111
							122	127
							82	96
							89	110
							80	87
							90	110
							109	122
							97	101
							71	115
1.574006	22.00556	336.62	0.634887	12.08336	-1.83758	-0.71992	74	106
							94	109
							94	106
							107	
							112	
							107	117
							88	
1.012649	12.24179	110.186	0.340637	5.31255	-2.07474	-0.52118	113	128
							77	
							88	
0.757383	18.47097	316.179	0.549481	12.14015	-1.46464	-0.67322	78	100
							72	
							96	120
0.55081	12.35816	189.186	0.710499	10.15567	-1.20211	-1.03023	85	88
0.575698	6.848244	283.914	0.358099	5.662332	-0.75441	-0.01162	82	97
							92	
1.679291	14.27689	312.113	0.864593	7.309022	-2.07329	-0.64826	90	103
1.214576	12.58379	287.648	0.683409	10.7344	-1.76527	-0.78793	92	106
							94	
0.421521	10.60925	134.772	0.219759	6.154219	-0.90051	-0.22047	98	107
							85	
0.465763	13.9077	303.915	0.492513	6.79821	-1.34893	-0.27762	70	106
							76	
							101	
0.705456	9.232255	150.661	0.364097	9.217706	-1.14826	-0.70612	78	107
							98	

0.358804	11.19815				-0.39761	1.088909	95	
0.823188	5.71997	476.407	0.340359	6.218065	-0.42444	0.281545	75	107
							104	
0.636414	7.472659				-0.67146	-0.51592	102	
1.408504	13.74507				-1.65116	-1.67561	105	
0.907947	10.24147				-1.20718	-1.09088	100	
3.076454	15.57095	192.904	0.637485	8.965017	-2.5069	-0.87834	119	120
							62	105
							103	70
1.095988	6.225911	271.759	0.231779	5.430164	-0.8546	0.255755	108	88
							51	71
							96	112
							64	103
							59	66
							73	113
							76	98
0.233479	11.00201	336.481	0.30586	8.945735	-0.50712	-0.09144	51	113
							99	78
							90	
							101	120
							83	
							93	
							79	105
							86	116
							102	105
							76	
							102	125
							110	
							59	112
							74	122
							71	
							109	105
							106	128
							101	105
							58	98
							105	
							95	111
							95	120
							115	85
							112	
							96	107
							105	100
							98	117
							111	
							108	
							96	
1.075891	13.41749	708.221	0.716091	5.531986	-0.83481	0.139043	102	112
							110	117
3.95819	23.79892	365.839	2.568577	14.22232	-2.68471	-1.60285	81	104
							76	92
							126	115

							94	
0.270229	14.26728	263.335	0.287851	10.02309	-0.65835	-0.27424	97	108
							107	
0.584826	24.06044	554.793	0.438885	9.754419	-1.6654	-0.05923	99	105
							85	
							115	
							90	81
0.383312	27.26138	645.883	0.680522	13.78955	-1.02244	-0.44657	94	118
0.181439	7.822784				0.259105	0.07555	108	
							78	109
1.298781	12.50319	278.513	0.238435	4.975603	-1.99928	0.307571	85	102
0.134632	6.880196	251.488	0.236442	4.805132	0.251278	0.271138	92	116
2.654078	11.07837	95.731	2.751973	6.147182	-2.29147	-1.93752	95	108
0.372393	11.85553	193.077	0.312775	7.875132	-1.16955	-0.36725	91	95
0.255277	7.734195	283.679	0.238329	4.653966	-0.1779	0.360682	78	112
							101	100
0.840053	12.79563	257.332	0.824448	5.952749	-1.41984	-0.61199	97	100
1.675083	7.458841				-1.33395	0.725466	86	
0.720357	12.32527	167.785	0.702886	9.482172	-1.10903	-1.05462	106	105
0.888499	10.1886	244.3	1.98292	10.53645	-1.27638	-1.51134	98	108
0.565374	6.8636	199.289	0.555069	8.476912	-0.31533	-0.74131	72	96
							103	101
0.870468	7.816703	422.402	0.497077	7.667023	-0.86836	-0.15526	104	107
1.167334	13.45123	179.786	1.846005	9.56481	-1.74807	-1.59305	107	116
0.558272	9.395895	505.841	0.214707	6.187648	-0.81703	0.608177	101	
0.590782	8.891957	164.69	0.313749	7.394246	-1.15734	-0.42772	113	124
0.805672	14.05473	238.433	0.527371	5.161981	-1.74921	-0.29945	60	107
							97	112
							60	104
0.6826	6.184098				-0.66138	0.202122	81	
0.665472	13.17312	157.601	0.605637	9.434743	-0.60809	-0.99982	109	111
1.269319	9.862371	374.726	1.168878	8.58573	-1.61924	-0.81745	78	96
2.004295	13.09219	431.074	1.265667	7.657259	-2.11117	-0.7117	84	103
0.666199	11.38681	203.627	0.310468	9.297141	-1.27311	-0.43154	88	
0.465122	7.835239	402.942	0.185789	5.381912	-0.21932	0.643566	83	110
0.964354	13.08688	266.704	0.808509	7.44597	-1.69073	-0.71412	86	109
0.473462	9.894433	241.039	0.531617	7.557416	-1.09028	-0.53016	89	109
0.461214	12.08205	291.293	0.607967	5.491414	-1.29531	-0.3018	97	109
							56	
0.140376	1.712322	484.247	0.116899	3.211743	1.214139	1.374318	108	108
0.272643	1.897271	369.701	0.196566	4.151033	0.820384	0.716743	74	99
0.470247	10.95553	346.539	0.150165	5.004649	-0.64196	0.727713	82	96
1.211173	6.533209	468.67	0.526099	6.117103	-0.43908	0.012571	98	110
							109	
1.483096	16.2282	210.367	0.525418	10.83969	-2.03308	-0.824	86	104
0.377861	10.29679	429.562	0.824288	11.97371	-0.67541	-0.72485	92	107
0.330859	7.934094	227.446	0.336941	9.420287	-0.22125	-0.42201	107	120
0.414626	9.590237	489.222	0.150717	6.403012	-0.08129	0.78696	112	118
							100	117
0.897994	13.91767	484.247	0.534073	8.460572	-1.16574		83	104
0.986722	15.9871	215.476	1.132382	9.83656	-1.38316	-1.21221	101	99

1.216236	15.06622	461.389	0.319615	7.960271	-2.00474	0.147849	72	104
							107	
							52	107
0.794674	11.68435	338.381	0.259262	7.450175	-1.15915		113	
1.969481	16.81066	206.333	1.317184	9.686386	-2.35808	-1.31897	113	116
							84	
3.265506	9.9659	196.794	0.610567	6.614182	-2.06292	-0.65641	105	112
0.2571	14.51147	258.617	0.195595	6.570463	-0.60286	0.212163	114	119
1.839395	22.5569	171.625	2.09242	17.76062	-2.47065	-2.05571	98	96
0.843287	8.629723	255.122	0.703551	7.203889	-0.80066	-0.63675	108	112
0.505818	4.098008	197.737	0.59069	5.009318	-0.1944	-0.46454	105	121
0.322441	8.161723	292.874	0.141178	6.340173	-0.01152	0.513757	75	76
0.324882	14.66376				-0.82812	-0.87075	91	
0.348193	16.89207				-0.69848	0.010277	90	
0.890462	13.32838	251.386	0.199202	6.478496	-1.39816	0.192045	108	114
1.015721	19.78871	223.133	1.090081	11.07631	-1.96626	-1.23948	82	103
							109	107
							104	113
4.831025	30.71968	69.665	1.657371	20.82746	-3.39967	-2.53158	93	115
							76	
							98	121
0.207536	6.652226	502.873	0.144195	4.94143	0.425153	0.994364	84	97
0.325409	9.559841	217.31	0.886848	9.711057	-1.09109	-1.05323	106	100
							105	
0.722391	12.37616	246.245	1.319233	8.773239	-1.06956	-1.15536	108	113
0.723766	18.26074	236.543	2.42178	21.81189	-1.63018		90	91
							67	78
							63	118
							77	107
							97	
							93	119
							93	
							78	66
							70	108
							78	108
							111	123
							98	115
							85	123
							105	126
							121	
							86	
1.498828	10.3667	360.742	1.760699	12.38052	-1.67628	-1.30525	115	107
0.113416	7.860468	149.366	0.123779	8.303207	-0.15337	0.011302	89	109
							115	
1.783786	30.18471	355.949	2.637125	13.37083	-2.6815	-1.59814	61	105
							95	
0.130655	4.764813	339.908	0.154427	5.294494	1.230694	0.663004	125	128
							83	107
1.91235	15.72857	161.142	0.614451	8.09404	-2.29349	-0.90298	104	104
0.657184	6.308174	177.61	0.350277	9.13216	-0.9726	-0.57723	78	116
0.118036	4.018678	421.684	0.15637	4.193279	1.418378	0.93601	72	88

							68	
0.841059	6.001092	210.479	0.860226	6.109241	-1.04763	-0.77512	65	112
							65	111
0.643557	11.55502	403.23	0.284207	9.565639	-0.58948	0.023845	89	105
							56	
0.320099	7.595984	288.843	0.446072	13.32775	-0.15202	-0.65823	80	97
							75	
							86	
4.435446	37.82701	273.906	1.585725	12.75798	-3.33719	-1.42452	82	100
1.970261	60.7725				-2.99597	-0.95843	98	
0.311236	10.84377	338.078	0.292482	7.715763	-0.41981	0.029994	109	119
0.337615	6.81392	171.636	0.425111	6.380949	-0.52184	-0.4978	106	107
1.948625	27.82434	0	0.302088	12.65206	-2.03086		114	96
1.468293	29.19171	266.002	1.345	10.28664	-2.13412	-1.21588	108	113
0.336324	10.65509	263.756	0.602722	8.484032	-0.31301	-0.62195	95	111
							115	
							68	95
0.675403	6.255127	376.38	0.207607	3.558453	-0.52119	0.790079	102	99
0.575865	11.68346	142.533	0.504943	8.405617	-1.37155	-0.88141	105	115
0.439138	8.796679	318.187	0.28599	6.912872	-0.42738	0.074181	92	118
							114	107
							47	
							100	116
							122	108
							106	117
0.768139	9.40936	208.673	1.244861	8.196732	-1.32865	-1.17901	125	108
0.677159	17.57726	130.795	1.647506	24.18738	-1.70032	-2.25312	98	102
							102	99
1.158941	5.479096	125.41	1.330556	8.533289	-0.73728	-1.54502	87	76
							123	116
0.162686	4.060109	552.632	0.234529	6.305368	1.343506	0.59653	86	101
0.403482	22.2143	135.886	0.319655	14.81007	-1.48188	-0.97586	104	
							79	
							52	105
							57	112
							100	118
							95	82
							88	97
							106	121
							120	110
							67	109
							57	
							103	
							87	
							116	
							81	105
							105	
							112	128
0.809104	7.079013	130.968	0.767515	5.448636	-1.31731	-0.92345	85	97
							109	
							105	

							101	124
0.252248	9.988338	311.302	0.66769	9.076407	-0.36621	-0.62445	109	113
0.824614	15.68857	124.098	1.253251	14.30461	-1.34896	-1.81981	85	103
0.24768	8.826822	325.188	0.14651	6.314411	-0.19296		95	101
							75	113
							117	112
							105	104
							109	137
2.565194	15.77377	213.729	0.572269	6.325926	-2.31864	-0.53993	113	113
2.307306	14.32359	361.9	1.321882	8.02195	-1.87331	-0.87169	108	84
0.207052	15.54206	187.017	0.417837	10.70602	-0.86876	-0.74918	115	99
1.36598	8.786021	273.791	1.083416	5.989199	-1.14775	-0.7436	98	108
0.663322	18.08443	267.656	0.250547	8.871766	-1.84781	-0.10437	108	108
							66	
0.762793	8.997012	284.586	1.609654	8.623724	-0.55557	-1.17752	102	105
0.678232	11.31754				-1.22923	-0.2671	111	
0.826592	7.416803	160.904	0.148762	5.552736	-1.0815	0.192037	104	108
							109	
0.162026	6.368871	555.776	0.129629	3.495197	0.502263		107	111
							88	
0.406434	9.616656				-0.49218	0.495569	112	
3.585727	20.73081	180.51	4.136102	14.34952	-2.63566	-2.29698	102	130
1.745343	24.47876	271.118	0.959879	9.655614	-2.3496	-0.96448	109	110
0.574448	11.50767	124.27	0.433152	7.420259	-1.17127	-0.79641	101	112
0.310329	11.52984	249.094	0.590834	8.216	-0.28089	-0.62509	97	108
							107	
0.408843	6.915249	356.901	0.23531	8.005106	-0.10809	0.174919	120	120
							106	112
							108	118
							94	101
							100	117
0.289236	5.758056	480.594	0.261641	7.877623	0.321358	0.30336	100	115
4.117224	21.41207	106.056	1.650176	17.84315	-2.89231	-2.19955	107	117
1.202394	16.88435	258.758	1.172723	9.582979	-1.4424	-1.1081	109	105
							105	121
0.28265	16.60211	237.862	0.270807	12.42698	-0.91623	-0.43038	123	124
							96	
							70	105
							105	
							118	126
0.758087	11.07803	334.283	0.233792	6.658601	-1.48587	0.252341	74	111
							116	105
							124	
							113	117
0.463773	5.684542	317.559	0.506511	8.165099	-0.18094	-0.37986	90	119
							95	
							108	
							105	
							109	
							119	116
							114	

							111	113
							93	
							114	
							97	
0.10711	7.267763	385.583	0.157534	6.437876	0.773624	0.607253	98	105
							115	
							118	107
							95	
0.54803	3.979269	213.73	0.324951	6.651958	-0.63736	-0.22524	104	109
0.222024	14.23334	358.046	0.198569	9.740488	-0.46231	0.160557	72	100
0.463907	14.60383	338.951	0.412435	5.140722	-0.60578	0.070042	94	94
							103	
							111	
0.80268	11.05393	253.912	0.455868	7.153739	-1.43744	-0.37137	98	115
							110	113
							122	108
							64	
1.002896	9.671634	281.646	0.232374	6.362494	-1.56353	0.178261	91	108
7.048655	20.37422	623.229	0.301054	6.066435	-2.82247	0.539924	79	124
0.219914	6.887544	243.414	0.237263	4.576438	-0.16537	0.278982	113	120
							116	117
							43	
0.386362	11.58483	170.632	1.47615	8.915258	-0.45844		114	
0.210913	7.957009	265.738	0.260433	8.25306	-0.13391	-0.08815	106	126
							105	
							115	
							124	125
1.657117	23.28396	80.292	0.856506	13.56051	-2.74483	-1.8206	91	105
0.452526	8.709876	419.841	0.175882	6.608963	-0.27128	0.575294	83	119
0.245028	6.854085	251.495	0.349056	5.155528	0.149419	-0.01279	74	97
0.159319	6.395345	589.622	0.125702	5.062314	0.895489	1.165598	125	123
0.190464	7.201821				0.088363	0.31083	121	
							119	109
							99	
							109	
							77	
0.411803	8.156139	290.231	0.292884	6.511133	-0.91238	0.039717	84	115
0.191868	18.73488	247.25	0.238497	11.3421	-0.74612	-0.27336	73	106
							91	
0.21591	7.802058				0.217296	0.068066	108	
							102	
0.419823	4.942543	197.154	0.281235	7.608316	-0.0006	-0.26845	105	117
							115	
							111	
							119	
							108	
0.893298	11.40742	228.801	1.134944	9.243655	-1.35401	-1.14056	116	118
0.334748	7.992599	432.142	0.255526	12.1713	-0.7874	-0.01635	117	112
0.190137	6.533713	161.95	0.153161	6.04102	-0.17015	0.125968	105	96
0.419441	11.46797				-1.09899	-0.49105	109	
0.255016	7.271384				-0.42107	-1.19373	111	

0.411584	21.99438	312.548	2.143477	15.75463	-1.37984	-1.6493	98	104
							108	
							111	
							112	75
							111	115
							70	
							104	113
							90	
							74	
							108	
							82	108
							121	
							120	
							94	
							114	100
							96	
							104	106
							94	
0.336274	7.21479	207.581	0.114535	4.549979	-0.45232	0.636319	95	117
							101	
							118	
							91	
							97	
0.586961	11.50231	318.031	0.395484	7.620428	-0.79994	-0.18536	111	111
1.735278	20.67969	226.024	0.462181	12.27159	-2.29079	-0.77814	97	106
							84	
0.232076	6.742607	250.572	0.244515	6.400214	0.129795	0.071	67	116
1.017166	5.524324	464.832	0.324837	3.971931	-0.39569	0.573713	113	107
							114	109
0.563446	9.245826	220.609	0.490032	6.623919	-1.00973	-0.45425	71	105
0.932969	14.0244	179.738	0.364539	8.005791	-1.79789	-0.51423	88	87
							78	106
							94	103
0.584677	16.04407	586.419	0.308711	11.36665	-1.12562	0.097404	117	115
1.438564	17.908	258.115	0.511751	10.10933	-1.84663	-0.64213	124	120
3.473168	14.80769	340.305	1.872052	9.309005	-2.30399	-1.20659	106	103
1.130783	15.56143	282.738	0.750581	9.149425	-1.60825	-0.75845	98	110
0.379868	9.335657				-0.43595	-0.42404	88	
0.203892	4.020555	474.172	0.172781	6.29865	0.472499	0.6923	112	113
0.292071	18.8662	156.306	0.852257	11.22598	-1.1175	-1.31291	117	109
2.378065	15.44992				-2.51082	-0.27114	92	
1.033542	15.44293	293.084	0.41801	8.349277	-1.71071	-0.32518	95	129
0.931219	14.61277	337.61	0.246398	6.871587	-1.5774	0.206493	100	116
0.363976	7.451329	285.281	0.333559	7.10727	-0.43692	-0.10477	113	122
0.574567	9.610733	242.096	0.234883	5.928249	-0.85041	0.121827	108	100
0.897314	11.80105	186.465	0.618286	8.316991	-1.42291	-0.83513	85	123
0.617067	10.11353	245.487	0.736424	8.921939	-0.88715	-0.81707	103	94
							120	
							71	
							105	
0.128488	4.112565	475.581	0.137928	4.551355	1.118009	1.038888	59	79

							118	98
6.772497	17.1454				-3.20216	-2.58084	113	
							99	
							100	106
							103	
							52	
							69	94
							78	
							68	115
							77	
0.203895	6.203659	216.958	0.493843	6.791122	0.154615	-0.48431	77	88
							53	83
							85	
							73	93
							106	
							65	105
							91	
							114	
							95	
							106	
							83	
							105	138
							95	105
							91	109
							109	
							71	85
0.527645	13.02942	195.7	1.302068	8.277694	-1.0716	-1.25	78	94
							64	136
1.500371	7.941642	445.558	0.909959	5.323147	-1.1811	-0.26948	108	115
0.706883	11.03444	439.387	0.491809	5.308247	-1.05662	0.101774	86	99
							69	100
							86	115
							88	
							95	121
1.012196	17.1853	488.288	0.326506	8.772852	-1.82191	0.109653	108	122
							91	89
							63	
							60	
							78	
							83	104
							86	72
							92	107
							79	118
							80	
0.366133	5.524554	134.298	0.374562	4.698825	-0.41052	-0.38367	117	108
							106	113
1.390483	36.95925	66.568	1.175811	21.95516	-2.779	-2.39167	74	113
							99	
							101	
							88	
							104	

							117	
							106	
							110	
0.289464	13.47977	322.876	0.252985	8.14235	-0.59162	0.057881	76	117
1.307599	32.26161	235.213	0.915277	18.44461	-2.15384	-1.40752	113	116
0.586069	9.631952	295.435	1.830388	7.61675	-0.77415	-1.15776	96	96
0.223405	6.784189	705.971	0.168643	6.044351	0.720194	0.982526	93	
0.153912	5.843213	348.918	0.202808	7.258157	0.580358	0.31354	76	96
							120	114
1.124581	16.91034	420.241	0.555853	9.675708	-1.67361	-0.3692	113	
1.045623	20.63283	0	2.471975	15.41554	-2.70047		63	94
							103	
							110	115
1.245189	15.93036	169.986	1.387804	14.98248	-1.82971	-1.72226	118	109
							90	91
							103	86
							108	
3.728463	19.49337	192.9	2.653248	11.74902	-3.08112	-1.88537	75	74
1.660455	6.618534				-0.95223	-1.03162	105	
0.324909	4.771717	410.377	0.233511	5.973599	0.368362	0.447633	102	98
0.652957	15.17632	162.558	0.420411	11.64049	-1.62927	-0.88799	105	99
3.164439	38.81052	78.896	2.39736	20.53718	-3.3131	-2.66248	86	117
1.728377	24.95522	325.688	0.694093	11.02664	-2.73787	-0.73847	104	103
0.694753	17.25348	196.673	0.916431	12.89071	-1.62611	-1.30169	103	106
							123	102
							101	111
1.830359	24.17758	235.521	1.051543	9.991471	-2.47223	-1.12412	100	110
0.201866	4.712577	281.326	0.147643	5.629921	0.406883	0.5348	97	91
0.268107	15.7308	405.079	0.126486	7.260629	-0.82428	0.699968	104	107
1.860814	26.70564	187.019	0.495943	14.34862	-2.86366	-1.02881	58	75
							106	116
							77	101
							104	
0.382126	13.48541	449.955	0.316778	7.125542	-0.77513	0.206314	112	113
0.365522	12.21792				-0.57245	-1.04782	114	
1.392609	10.58439	357.32	1.324459	12.57597	-1.07056	-1.15042	110	110
0.269729	6.599234	264.974	0.186963	5.134814	0.106532	0.407983	106	82
							51	100
1.544833	10.47663	247.275	1.1205	8.862669	-1.20057	-1.06118	115	123
0.420401	9.935482	390.638	0.247777	6.450122	-0.44988	0.332575	98	95
0.26852	3.810362				-0.06485	-0.10842	94	
1.113523	10.85536	323.714	0.795677	5.461469	-1.02108	-0.39834	105	115
0.778282	8.980654	512.552	0.523095	11.03703	-1.15504	-0.29119	89	113
							107	113
1.187961	20.11738	279.397	1.446996	6.764455	-1.45118	-0.97921	92	109
0.815569	9.102645	258	0.535709	9.616173	-0.99125	-0.63982	100	112
7.863444	37.39799	268.112	0.336477	8.460132	-3.79085	-0.25501	92	111
0.232027	6.604078	339.558	0.295852	7.483537	0.214355		97	119
							86	102
0.422728	20.52081	288.852	0.339427	9.812291	-1.08314	-0.30547	97	106
0.234458	14.97656	366.232	0.195543	6.206773	-0.23313	0.463389	113	123

							109	
1.588902	19.90143	213.368	0.991094	14.88918	-2.08297	-1.38551	97	104
0.941623	15.14709	304.369	0.081905	2.767395	-1.43855	1.399802	96	105
1.928042	14.48393	376.702	0.852721	5.979506	-1.72197	-0.40338	70	101
0.218041	7.014193	566.506	0.14395	3.904221	0.542934	1.218677	92	110
0.967203	11.55545	469.312	0.553627	7.728196	-0.78248	-0.16163	74	99
1.514804	15.80633	239.085	0.430934	7.916221	-1.84142	-0.43554	100	111
0.35168	16.07063	383.308	0.162467	10.35003	-0.71762		95	107
0.658954	11.87923	259.573	0.415988	9.409294	-1.15209	-0.46921	102	105
2.222123	20.75772	113.01	0.839799	14.23595	-2.56983	-1.63687	80	79
1.571729	15.76176	458.541	0.240976	6.136554	-1.78292	0.480329	78	103
2.176514	29.18773	371.807	0.907348	12.1055	-2.5204	-0.87662	103	102
0.472747	10.21663				-0.74841	-0.84866	46	46
0.256698	8.160916	603.359	0.178035	7.654697	0.239344	0.702298	52	106
1.551016	12.9978	476.21	0.237395	6.126666	-1.23664	0.51417	115	112
1.081331	13.86431				-1.3899	-0.24569	72	
0.335717	12.17758	254.004	0.541129	12.97651	-0.78585	-0.83661	95	98
0.64483	16.70654	224.773	0.36146	8.44913	-1.78259	-0.40571	56	118
0.219398	6.827664	331.823	0.245959	5.167621	0.183079	0.373435	101	114
0.647383	7.032049	307.679	1.333449	8.791111	-0.63482	-1.02959	80	97
1.03796	15.07033	385.575	0.341227	9.155791	-1.3408	-0.08925	94	117
1.193747	18.28831	443.199	0.928737	10.26897	-1.54502	-0.68518	95	106
							90	
0.824265	7.629927	291.976	0.213153	5.925242	-1.0158	0.298042	109	128
							111	
1.053889	6.637861	241.899	0.999813	6.833196	-1.01444	-0.84951	108	109
							103	
							64	
0.525567	11.19033	402.889	0.226716	6.882168	-0.70536	0.366612	81	101
							55	
3.173757	19.42049				-2.58773		97	
1.298461	10.36356				-1.62101	0.213041	77	
0.349543	13.46805	198.05	0.566115	7.86747	-0.9613	-0.71187	81	102
0.626822	11.17832	188.824	0.423335	5.837444	-1.10232	-0.38284	79	106
							101	85
0.887214	10.60457	426.022	0.377344	5.081747	-0.98528		92	115
4.326834	27.43103	380.324	1.630753	18.72898	-3.06734		95	108
0.465083	10.90293	169.9225	0.302757	10.91815	-0.53221	-0.62401	62	81
							114	
1.228077	16.55978	475.492	0.319056	13.23674	-1.5637	-0.14563	115	113
0.31041	11.72256	390.629	0.267114	9.126646	-0.69266	0.071459	73	90
							109	
0.826498	20.23512	124.271	0.318944	14.79544	-1.37493	-1.02759	99	110
							67	
0.231699	8.666049	311.798	0.16037	6.95514	-0.15752	0.415881	66	76
11.88227	23.10436	47.822	1.374572	14.60281	-4.05299	-2.43694	122	123
0.275824	8.12873				0.252818	0.043944	107	
2.958757	41.88818	303.826	0.587756	16.5328	-3.53968	-0.92443	97	107
0.201814	5.493305				0.108365	0.038836	98	
							137	
							86	104

0.883556	13.25063	219.722	0.590228	11.63293	-1.81491	-0.91045	107	69
							113	118
							95	
							85	
							84	109
							85	
							110	
							61	
							99	
							115	
							90	
							113	112
							120	118
							100	138
							70	
0.250063	4.661262	116.529	0.514857	6.460375	-0.8671	-0.8559	126	120
							101	
							94	
							115	
0.995652	7.802356	298.712	0.596184	5.798712	-1.0981	-0.30775	113	120
							102	
1.0577	6.740408	512.226	0.241459	5.450244	-1.10248	0.621901	84	121
							113	113
							116	
1.314434	30.68626	445.701	0.475199	12.29732	-2.13158	-0.38395	75	106
0.286874	6.737368	498.174	0.251572	4.528751	0.352529	0.694527	104	100
							65	31
							110	116
							91	
1.493951	10.8101	253.408	0.270388	5.056066	-1.45621	0.161374	115	121
0.154585	5.864271	671.593	0.336065	7.207362	0.719155	0.41082	91	90
1.043147	7.288336	277.082	0.762231	6.443783	-0.91617	-0.56758	83	88
0.659263	10.77405	420.424	0.246518	7.464085	-0.81449	0.290784	69	106
							101	110
1.241382	17.63949	111.058	0.563338	9.75137	-2.23508	-1.18587	112	119
							113	103
0.746842	13.79287	396.411	0.238756	8.387745	-1.5544	0.201971	80	99
0.30969	3.393489	344.337	0.231193	7.646523	0.474933	0.192005	120	105
2.124282	16.80802	146.047	1.076933	11.25125	-2.46879	-1.49339	98	109
							87	114
0.458579	12.08733	323.067	0.289642	9.467466	-0.90265	-0.11778	105	
0.138716	4.03544	463.752	0.150004	4.744486	0.997722	0.944262	46	124
							107	
1.351368	15.15958	242.551	0.342349	8.458227	-1.52366	-0.32673	105	121
0.467449	2.159891	238.966	0.700229	5.105358	0.349919	-0.46437	67	108
							98	
0.187795	5.138656	385.554	0.156604	4.230674	0.465303	0.873342	79	107
0.30428	4.653272	271.378	0.265817	3.77031	0.170039	0.396107	55	95
0.384671	13.84487				-1.16021	0.072455	92	
0.208222	4.812499	161.377	0.116672	2.869679	0.067143	0.755394	71	109
0.962901	18.78785	209.649	0.422496	9.261284	-1.74739	-0.59887	68	104

							51	63
1.571685	13.39078				-1.75335	-1.34027	123	
							93	103
							112	
							58	
							64	
							98	104
							82	
0.908366	11.0253	64.392	1.228213	10.21258	-2.03099	-1.99424	66	102
0.366982	14.17128	226.047	0.243563	10.10312	-0.7568	-0.2703	110	110
							94	
							96	99
							95	122
0.119399	4.234678	537.031	0.158412	5.144498	0.735945	0.951308	118	
							127	
							126	
							111	124
0.662552	10.42686	183.882	0.243049	7.815236	-0.90488	-0.23821	126	117
0.639518	7.749768	353.732	0.238223	3.505962	-0.40878		101	120
1.390081	8.674273	235.705	0.469741	6.302061	-1.42064	-0.35771	102	116
							100	
0.530936	24.85467	289.903	0.350553	12.34151	-1.68991	-0.46288	102	108
0.226028	2.804894	120.844	0.815562	8.235971	0.830795	-1.25549	78	96
1.43757	42.03847	171.187	2.910464	9.751986	-2.88736	-1.90034	110	112
1.160902	9.646035	364.899	0.337759	5.650026	-1.20941	0.180575	115	116
0.335896	10.08065	142.355	0.864377	6.1384	-0.9201	-1.01642	99	114
0.531911	13.60512	403.018	0.395586	6.269344	-0.90917	0.080129	74	106
							86	110
0.594582	13.90774	368.898	0.335799	7.93059	-0.98935	-0.01824	107	98
0.208085	7.615282	501.823	0.409818	9.761273	0.552943	-0.07922	87	113
							105	
0.267952	5.102222	436.719	0.269158	4.215264	0.173153	0.614963	104	79
0.207348	3.990407	351.228	0.223017	3.976279	0.677959	0.632778	112	122
0.322351	13.34835				-0.68665	0.267519	97	
							91	
0.822458	6.128658	256.936	0.553811	5.730148	-0.29345	-0.34749	113	84
0.181386	7.114823	284.72	0.24503	6.151817	0.149354	0.173005	109	100

anaemia_a	anaemia_e	hb_cat_ad	hb_cat_exi	SFAI_adm	SFAI_exit	sTfRAI_adr	sTfRAI_exi	ID_adm
Yes	Yes	Hb_adm<1	Hb_exit<1	52.22728	52.22728	5.554462	5.554462	No
No	No	Hb_adm>=	Hb_exit<1	18.76222	18.76222	9.237684	9.237684	No
No		Hb_adm>=110		12.50235	12.50235	10.74024	10.74024	No
No	No	Hb_adm>=	Hb_exit<1	30.196	30.196	9.056914	9.056914	No
Yes	Yes	Hb_adm<1	Hb_exit<1	8.9657	8.9657	12.26895	12.26895	Yes
Yes		Hb_adm<110						
Yes	Yes	Hb_adm<1	Hb_exit<1	13.26715	13.26715	4.91421	4.91421	No
Yes	Yes	Hb_adm<1	Hb_exit<1	12.08211	12.08211	39.68294	39.68294	No
No		Hb_adm>=110		8.680145		8.038543		Yes
Yes		Hb_adm<110		1.57032	1.57032	16.09603	16.09603	Yes
Yes	Yes	Hb_adm<1	Hb_exit<1	41.3251	41.3251	4.484792	4.484792	No
Yes		Hb_adm<110		6.487833	6.487833	9.9158	9.9158	Yes
Yes	No	Hb_adm<1	Hb_exit<1	10.80422	3.665112	6.395438	10.93759	Yes
Yes	Yes	Hb_adm<1	Hb_exit<1	54.38659	84.13298	15.25303	2.672641	No
No	No	Hb_adm>=	Hb_exit<1	43.20545	43.20545	6.27736	6.27736	No
Yes	Yes	Hb_adm<1	Hb_exit<1	5.155142	5.155142	13.16716	13.16716	Yes
No		Hb_adm>=110		51.93679	51.93679	6.350133	6.350133	No
No	No	Hb_adm>=	Hb_exit<1	17.64683	17.64683	6.681858	6.681858	No
No		Hb_adm>=110		23.01311	23.01311	5.456139	5.456139	No
Yes	Yes	Hb_adm<1	Hb_exit<1	4.083048	1.499876	8.23699	5.445122	Yes
Yes	Yes	Hb_adm<1	Hb_exit<1	39.22769	39.22769	18.38544	18.38544	No
Yes	Yes	Hb_adm<1	Hb_exit<1	49.6096	49.6096	4.928461	4.928461	No
Yes	No	Hb_adm<1	Hb_exit<1	7.143939	7.143939	5.873792	5.873792	Yes
Yes		Hb_adm<110						
Yes		Hb_adm<110		19.81392	19.81392	5.101151	5.101151	No
Yes	Yes	Hb_adm<1	Hb_exit<1	98.01836	98.01836	13.2031	13.2031	No
Yes	Yes	Hb_adm<1	Hb_exit<1	45.28687	45.28687	15.58146	15.58146	No
Yes	Yes	Hb_adm<1	Hb_exit<1	6.982912	6.982912	25.70184	25.70184	Yes
Yes	Yes	Hb_adm<1	Hb_exit<1	0.372967	3.927735	29.10639	6.792043	Yes
No	Yes	Hb_adm>=	Hb_exit<1	23.66484	23.66484	4.673318	4.673318	No
Yes	No	Hb_adm<1	Hb_exit<1	10.09547	10.09547	9.300396	9.300396	Yes
Yes		Hb_adm<110		7.494846	7.494846	9.269552	9.269552	Yes
No	No	Hb_adm>=	Hb_exit<1	19.08053	19.08053	5.610407	5.610407	No
No	No	Hb_adm>=	Hb_exit<1	7.084909	7.084909	6.876646	6.876646	Yes
Yes	No	Hb_adm<1	Hb_exit<1	13.52357	13.52357	9.061215	9.061215	No
No	No	Hb_adm>=	Hb_exit<1	40.19474	40.19474	4.601352	4.601352	No
No	No	Hb_adm>=	Hb_exit<1	7.186906	6.426235	8.450385	11.30873	Yes
No	Yes	Hb_adm>=	Hb_exit<1	35.175	15.28568	6.345	5.832119	No
Yes	Yes	Hb_adm<1	Hb_exit<1	14.77241	101.5292	12.45872	4.450588	No
Yes	No	Hb_adm<1	Hb_exit<1	7.218545	7.218545	6.023703	6.023703	Yes
Yes	Yes	Hb_adm<1	Hb_exit<1	3.797865	3.797865	13.10108	13.10108	Yes
Yes	Yes	Hb_adm<1	Hb_exit<1	1.310407	1.310407	13.66633	13.66633	Yes
Yes		Hb_adm<110		11.70173	11.70173	9.264328	9.264328	Yes
Yes		Hb_adm<110		8.146289	8.146289	6.505944	6.505944	Yes
Yes	No	Hb_adm<1	Hb_exit<1	36.06109	36.06109	1.621449	1.621449	No
Yes	No	Hb_adm<1	Hb_exit<1	45.94252	26.7836	6.742353	10.81686	No
No		Hb_adm>=110		24.645	24.645	5.928038	5.928038	No
Yes	No	Hb_adm<1	Hb_exit<1	34.56753	39.21371	5.249384	10.14191	No
No	No	Hb_adm>=	Hb_exit<1	16.5111	16.5111	8.094405	8.094405	No
Yes	No	Hb_adm<1	Hb_exit<1	33.06088	33.06088	5.107193	5.107193	No

Yes	Yes	Hb_adm<1	Hb_exit<1:	134.9544	134.9544	7.817788	7.817788	No
Yes	Yes	Hb_adm<1	Hb_exit<1:	9.427966	9.427966	13.71339	13.71339	Yes
Yes	Yes	Hb_adm<1	Hb_exit<1:	11.03622	11.03622	26.57862	26.57862	Yes
Yes	Yes	Hb_adm<1	Hb_exit<1:	17.53679	17.53679	7.385091	7.385091	No
Yes	No	Hb_adm<1	Hb_exit<1:	9.556003	9.556003	7.768468	7.768468	Yes
Yes	No	Hb_adm<1	Hb_exit<1:	10.05709	67.23848	9.354885	2.069413	Yes
Yes	Yes	Hb_adm<1	Hb_exit<1:	23.5647	23.5647	18.79966	18.79966	No
No		Hb_adm>=110		43.0965	43.0965	5.763716	5.763716	No
Yes	Yes	Hb_adm<1	Hb_exit<1:	5.105331	5.105331	10.60623	10.60623	Yes
Yes		Hb_adm<110						
Yes	Yes	Hb_adm<1	Hb_exit<1:	5.336765	5.336765	14.79621	14.79621	Yes
No	No	Hb_adm>=	Hb_exit<1:	40.01822	40.01822	5.150105	5.150105	No
Yes	Yes	Hb_adm<1	Hb_exit<1:	31.5488	31.5488	29.53987	29.53987	No
Yes	No	Hb_adm<1	Hb_exit<1:	17.56712	10.6328	10.74835	14.90284	No
Yes		Hb_adm<110		3.974777	3.974777	9.274343	9.274343	Yes
Yes	No	Hb_adm<1	Hb_exit<1:	2.748792	2.748792	13.55142	13.55142	Yes
Yes	Yes	Hb_adm<1	Hb_exit<1:	2.395191	2.395191	9.496624	9.496624	Yes
Yes		Hb_adm<110		10.27613	10.27613	7.259402	7.259402	Yes
No	No	Hb_adm>=	Hb_exit<1:	7.185784	7.185784	6.090583	6.090583	Yes
Yes	Yes	Hb_adm<1	Hb_exit<1:	4.929633	29.5501	16.48013	8.134992	Yes
No	No	Hb_adm>=	Hb_exit<1:	48.99269	48.99269	5.783182	5.783182	No
Yes	Yes	Hb_adm<1	Hb_exit<1:	23.55	23.55	7.13	7.13	No
Yes	Yes	Hb_adm<1	Hb_exit<1:	6.325202	6.325202	8.348016	8.348016	Yes
Yes	No	Hb_adm<1	Hb_exit<1:	36.02286	36.02286	6.08235	6.08235	No
No	No	Hb_adm>=	Hb_exit<1:	14.30836	14.30836	7.739808	7.739808	No
No		Hb_adm>=110		5.145	5.145	9.19	9.19	Yes
Yes	No	Hb_adm<1	Hb_exit<1:	26.38	26.38	8.97	8.97	No
Yes		Hb_adm<110		67.60448	67.60448	24.91396	24.91396	No
Yes	Yes	Hb_adm<1	Hb_exit<1:	7.913492	7.913492	18.98458	18.98458	Yes
No	No	Hb_adm>=	Hb_exit<1:	18.8988	18.8988	7.186841	7.186841	No
Yes		Hb_adm<110		4.217543		24.62792		Yes
No	No	Hb_adm>=	Hb_exit<110					
Yes	Yes	Hb_adm<1	Hb_exit<1:	4.288889	4.288889	13.49235	13.49235	Yes
Yes		Hb_adm<110						
No		Hb_adm>=110		17.615	17.615	9.625	9.625	No
Yes		Hb_adm<110		3.057754	3.057754	34.18574	34.18574	Yes
No		Hb_adm>=110		10.75248	10.75248	6.562413	6.562413	Yes
Yes		Hb_adm<110		40.19892	40.19892	7.850294	7.850294	No
Yes	Yes	Hb_adm<1	Hb_exit<1:	12.58248	12.58248	10.93567	10.93567	No
Yes		Hb_adm<110		47.02598	47.02598	4.251713	4.251713	No
Yes		Hb_adm<110		3.517598	3.517598	31.01984	31.01984	Yes
Yes	Yes	Hb_adm<1	Hb_exit<1:	0.762303	4.950089	16.53022	10.63218	Yes
Yes	No	Hb_adm<1	Hb_exit<1:	14.5	9.255991	6.105	10.07565	No
Yes		Hb_adm<110		8.978599		14.45866		Yes
Yes	Yes	Hb_adm<1	Hb_exit<1:	10.14204	11.65535	7.435881	9.780898	Yes
No	No	Hb_adm>=	Hb_exit<1:	14.14788	14.14788	6.361514	6.361514	No
Yes		Hb_adm<110		2.318313	2.318313	22.61248	22.61248	Yes
Yes	No	Hb_adm<1	Hb_exit<1:	2.406536	2.406536	11.91026	11.91026	Yes
Yes		Hb_adm<110		5.406145	5.406145	19.17861	19.17861	Yes
Yes		Hb_adm<110		47.13666	47.13666	5.989016	5.989016	No
Yes	No	Hb_adm<1	Hb_exit<1:	16.83869	16.83869	6.365399	6.365399	No

Yes	Yes	Hb_adm<1	Hb_exit<110					
Yes	No	Hb_adm<1	Hb_exit<1:	9.22378	9.22378	6.076991	6.076991	Yes
Yes	Yes	Hb_adm<1	Hb_exit<1:	7.607156	7.607156	9.06837	9.06837	Yes
Yes	No	Hb_adm<1	Hb_exit<110					
Yes	Yes	Hb_adm<1	Hb_exit<1:	54.01048	54.01048	13.01023	13.01023	No
No		Hb_adm>=110		16.3874	16.3874	9.38204	9.38204	No
Yes	Yes	Hb_adm<1	Hb_exit<1:	4.345182	4.345182	13.19933	13.19933	Yes
No	No	Hb_adm>=	Hb_exit<1:	11.12317	11.12317	6.042094	6.042094	Yes
Yes	No	Hb_adm<1	Hb_exit<1:	11.13	11.13	8.26	8.26	Yes
Yes		Hb_adm<110		18.02	3.151749	8.53	11.46536	No
No	Yes	Hb_adm>=	Hb_exit<1:	16.58	16.58	5.78	5.78	No
Yes	Yes	Hb_adm<1	Hb_exit<1:	9.749607	9.749607	7.709252	7.709252	Yes
Yes	Yes	Hb_adm<1	Hb_exit<110					
No		Hb_adm>=110		37.33341	37.33341	8.510354	8.510354	No
Yes		Hb_adm<110		5.709337	5.709337	12.21227	12.21227	Yes
Yes		Hb_adm<110		5.548557	5.548557	7.306453	7.306453	Yes
No	Yes	Hb_adm>=	Hb_exit<1:	11.25641	11.25641	5.826751	5.826751	Yes
No	No	Hb_adm>=	Hb_exit<1:	23.32927	23.32927	5.202404	5.202404	No
No		Hb_adm>=110		45.76774	45.76774	4.348117	4.348117	No
Yes		Hb_adm<110		16.93967	16.93967	6.69109	6.69109	No
No	Yes	Hb_adm>=	Hb_exit<1:	77.8943	46.06663	6.071292	6.422466	No
Yes	No	Hb_adm<1	Hb_exit<1:	9.048721	9.048721	10.55727	10.55727	Yes
No		Hb_adm>=110		52.48531	52.48531	5.111928	5.111928	No
Yes	Yes	Hb_adm<1	Hb_exit<110					
Yes	No	Hb_adm<1	Hb_exit<1:	25.55593	25.55593	4.855497	4.855497	No
Yes	No	Hb_adm<1	Hb_exit<1:	12.04285	12.04285	10.77106	10.77106	No
Yes		Hb_adm<110		1.882295	1.882295	29.22732	29.22732	Yes
No		Hb_adm>=110		14.46788	14.46788	5.236244	5.236244	No
No	Yes	Hb_adm>=	Hb_exit<1:	14.00343	14.00343	6.459575	6.459575	No
Yes	Yes	Hb_adm<1	Hb_exit<1:	12.32034	40.50609	5.029106	2.834232	No
Yes	Yes	Hb_adm<1	Hb_exit<1:	35.97665	35.97665	3.057822	3.057822	No
Yes	Yes	Hb_adm<1	Hb_exit<1:	10.97799	10.97799	15.4576	15.4576	Yes
Yes	Yes	Hb_adm<1	Hb_exit<1:	21.42138	21.42138	7.514981	7.514981	No
Yes	No	Hb_adm<1	Hb_exit<1:	2.323727	2.323727	8.496919	8.496919	Yes
Yes	Yes	Hb_adm<1	Hb_exit<1:	6.097963	6.097963	5.98646	5.98646	Yes
Yes	Yes	Hb_adm<1	Hb_exit<1:	12.84135	12.84135	15.73201	15.73201	No
Yes	No	Hb_adm<1	Hb_exit<1:	32.0101	32.0101	6.989929	6.989929	No
No		Hb_adm>=110		25.61836	25.61836	5.128472	5.128472	No
No	No	Hb_adm>=	Hb_exit<1:	5.094705	5.094705	6.428717	6.428717	Yes
Yes		Hb_adm<110		7.513431	7.513431	9.10042	9.10042	Yes
Yes	Yes	Hb_adm<1	Hb_exit<1:	28.30087	28.30087	7.583384	7.583384	No
Yes	No	Hb_adm<1	Hb_exit<1:	14.04985	14.04985	8.511057	8.511057	No
No	Yes	Hb_adm>=	Hb_exit<1:	36.66477	36.66477	3.576324	3.576324	No
Yes		Hb_adm<110		5.610872	5.610872	8.584583	8.584583	Yes
Yes		Hb_adm<110		21.92443	21.92443	6.82724	6.82724	No
Yes	No	Hb_adm<1	Hb_exit<1:	72.26475	72.26475	19.13235	19.13235	No
No	No	Hb_adm>=	Hb_exit<1:	24.38776	24.38776	11.15942	11.15942	No
Yes	Yes	Hb_adm<1	Hb_exit<1:	13.38712	13.38712	5.218962	5.218962	No
Yes	Yes	Hb_adm<1	Hb_exit<1:	2.884552	2.884552	17.78116	17.78116	Yes
Yes		Hb_adm<110		53.68381	53.68381	6.889684	6.889684	No
Yes	Yes	Hb_adm<1	Hb_exit<110					

Yes	Yes	Hb_adm<1	Hb_exit<1:	73.32078	73.32078	10.78197	10.78197	No
Yes		Hb_adm<110						
Yes	No	Hb_adm<1	Hb_exit<110					
Yes		Hb_adm<110		20.32226	20.32226	14.1976	14.1976	No
Yes		Hb_adm<110						
Yes		Hb_adm<110						
Yes	Yes	Hb_adm<1	Hb_exit<1:	6.229085	6.229085	23.60259	23.60259	Yes
Yes	Yes	Hb_adm<1	Hb_exit<1:	4.943566	4.943566	6.42233	6.42233	Yes
Yes	No	Hb_adm<1	Hb_exit<1:	16.64328	16.64328	7.60975	7.60975	No
No	Yes	Hb_adm>=	Hb_exit<110					
Yes	Yes	Hb_adm<1	Hb_exit<1:	23.36776	23.36776	9.372984	9.372984	No
Yes		Hb_adm<110		5.850402	5.850402	18.07486	18.07486	Yes
Yes		Hb_adm<110		40.761	40.761	5.989845	5.989845	No
Yes		Hb_adm<110		0.686291	0.686291	23.63489	23.63489	Yes
Yes		Hb_adm<110		3.459589	3.459589	26.348	26.348	Yes
Yes		Hb_adm<110		4.873563	4.873563	12.12863	12.12863	Yes
Yes	No	Hb_adm<1	Hb_exit<1:	8.93	8.93	8.145	8.145	Yes
No	No	Hb_adm>=	Hb_exit<1:	22.805	30.51677	5.365	4.855693	No
Yes	Yes	Hb_adm<1	Hb_exit<1:	15.66182	15.66182	10.59048	10.59048	No
Yes	No	Hb_adm<1	Hb_exit<1:	21.72028	21.72028	6.475754	6.475754	No
Yes	Yes	Hb_adm<1	Hb_exit<1:	48.69935	48.69935	3.108992	3.108992	No
Yes	No	Hb_adm<1	Hb_exit<1:	58.78976	58.78976	6.94583	6.94583	No
Yes	No	Hb_adm<1	Hb_exit<1:	11.44211	11.44211	7.085487	7.085487	Yes
Yes	Yes	Hb_adm<1	Hb_exit<1:	15.83223	15.83223	8.329897	8.329897	No
Yes	No	Hb_adm<1	Hb_exit<1:	14.22679	14.22679	14.83596	14.83596	No
Yes	Yes	Hb_adm<1	Hb_exit<1:	1.346527	1.346527	19.58783	19.58783	Yes
Yes	Yes	Hb_adm<1	Hb_exit<1:	17.91372	17.91372	7.051275	7.051275	No
Yes	Yes	Hb_adm<1	Hb_exit<1:	1.069751	1.069751	17.93031	17.93031	Yes
Yes		Hb_adm<110		29.86327	29.86327	5.19709	5.19709	No
No		Hb_adm>=110						
Yes	No	Hb_adm<1	Hb_exit<1:	8.676991	8.676991	5.482123	5.482123	Yes
Yes		Hb_adm<110		10.51281	10.51281	8.663511	8.663511	Yes
No	No	Hb_adm>=	Hb_exit<1:	39.64479	39.64479	6.684704	6.684704	No
Yes		Hb_adm<110						
Yes		Hb_adm<110		38.33413	38.33413	13.5445	13.5445	No
Yes	Yes	Hb_adm<1	Hb_exit<1:	9.268842	9.268842	18.83807	18.83807	Yes
Yes		Hb_adm<110		40.36144	40.36144	9.558571	9.558571	No
Yes	No	Hb_adm<1	Hb_exit<1:	41.71839	41.71839	12.4715	12.4715	No
Yes	Yes	Hb_adm<1	Hb_exit<1:	23.85341	23.85341	9.549941	9.549941	No
Yes	Yes	Hb_adm<1	Hb_exit<1:	14.06	14.06	8.49	8.49	No
Yes		Hb_adm<110		89.5702	89.5702	12.70942	12.70942	No
Yes	Yes	Hb_adm<1	Hb_exit<1:	39.78082	39.78082	14.23124	14.23124	No
Yes	Yes	Hb_adm<1	Hb_exit<1:	30.4899	30.4899	18.31945	18.31945	No
Yes		Hb_adm<110		33.94155	33.94155	8.110086	8.110086	No
Yes	Yes	Hb_adm<1	Hb_exit<1:	11.36867	11.36867	8.73515	8.73515	Yes
Yes		Hb_adm<110		1.300063	1.300063	38.00482	38.00482	Yes
Yes	Yes	Hb_adm<1	Hb_exit<1:	27.57266	27.57266	11.01305	11.01305	No
Yes		Hb_adm<110		47.44954	47.44954	11.78862	11.78862	No
Yes		Hb_adm<110		32.29922	32.29922	15.03155	15.03155	No
Yes	Yes	Hb_adm<1	Hb_exit<1:	36.69532	36.69532	23.60466	23.60466	No
Yes		Hb_adm<110		76.47504	76.47504	7.014962	7.014962	No

Yes		Hb_adm<110		5.687269	5.687269	14.94789	14.94789	Yes
Yes	Yes	Hb_adm<1	Hb_exit<1:	37.07941	37.07941	18.45426	18.45426	No
Yes		Hb_adm<110		52.06278	52.06278	6.018726	6.018726	No
Yes		Hb_adm<110		36.89481	36.89481	9.883712	9.883712	No
Yes		Hb_adm<110		15.88392	6.998407	4.478805	14.64033	No
Yes		Hb_adm<110		8.15042	8.15042	5.515492	5.515492	Yes
No	No	Hb_adm>=	Hb_exit<1:	15.79011	15.79011	6.589789	6.589789	No
Yes	Yes	Hb_adm<1	Hb_exit<110					
Yes	Yes	Hb_adm<1	Hb_exit<1:	6.38277	6.38277	7.31752	7.31752	Yes
Yes	Yes	Hb_adm<1	Hb_exit<1:	37.18424	37.18424	4.893558	4.893558	No
Yes	Yes	Hb_adm<1	Hb_exit<1:	38.08422	38.08422	10.24849	10.24849	No
Yes	No	Hb_adm<1	Hb_exit<110					
Yes	Yes	Hb_adm<1	Hb_exit<1:	22.01375	22.01375	5.088388	5.088388	No
Yes	Yes	Hb_adm<1	Hb_exit<110					
Yes	No	Hb_adm<1	Hb_exit<1:	77.42505	77.42505	9.795056	9.795056	No
Yes	Yes	Hb_adm<1	Hb_exit<1:	7.245989	7.245989	7.893439	7.893439	Yes
Yes	No	Hb_adm<1	Hb_exit<1:	33.13098	13.97972	16.8501	8.08496	No
Yes	Yes	Hb_adm<1	Hb_exit<1:	12.45202	12.45202	8.078341	8.078341	No
Yes		Hb_adm<110						
Yes	No	Hb_adm<1	Hb_exit<1:	3.02844	3.02844	15.24419	15.24419	Yes
Yes		Hb_adm<110		12.86523	12.86523	6.648743	6.648743	No
Yes		Hb_adm<110						
Yes	Yes	Hb_adm<1	Hb_exit<1:	1.814691	1.814691	18.43059	18.43059	Yes
Yes	No	Hb_adm<1	Hb_exit<110					
Yes	Yes	Hb_adm<1	Hb_exit<1:	16.38586	16.38586	10.07182	10.07182	No
Yes		Hb_adm<110		5.759342	5.759342	20.91162	20.91162	Yes
Yes	No	Hb_adm<1	Hb_exit<1:	23.09678	23.09678	3.751468	3.751468	No
No		Hb_adm>=110		15.98129	15.98129	5.619037	5.619037	No
Yes	No	Hb_adm<1	Hb_exit<1:	52.82881	52.82881	5.167782	5.167782	No
Yes	No	Hb_adm<1	Hb_exit<1:	2.343926	16.31672	33.71524	7.787278	Yes
Yes		Hb_adm<110		13.11755	13.11755	9.070537	9.070537	No
Yes	Yes	Hb_adm<1	Hb_exit<1:	18.14044	18.14044	6.001201	6.001201	No
Yes	No	Hb_adm<1	Hb_exit<1:	9.694844	9.694844	8.054209	8.054209	Yes
Yes	Yes	Hb_adm<1	Hb_exit<1:	29.48477	29.48477	5.958129	5.958129	No
Yes	Yes	Hb_adm<1	Hb_exit<1:	46.92273	56.61038	17.44679	19.18335	No
Yes		Hb_adm<110						
Yes	No	Hb_adm<1	Hb_exit<1:	3.268648	3.268648	12.04393	12.04393	Yes
Yes	No	Hb_adm<1	Hb_exit<1:	45.59022	45.59022	7.158941	7.158941	No
No	Yes	Hb_adm>=	Hb_exit<1:	22.30906	22.30906	6.428234	6.428234	No
No		Hb_adm>=110		11.88654	11.88654	9.043991	9.043991	Yes
Yes	Yes	Hb_adm<1	Hb_exit<1:	55.80855	55.80855	6.227394	6.227394	No
Yes	Yes	Hb_adm<1	Hb_exit<1:	9.72085	9.72085	5.361188	5.361188	Yes
Yes	No	Hb_adm<1	Hb_exit<1:	3.572924	3.572924	16.95052	16.95052	Yes
No		Hb_adm>=110		18.90922	18.90922	5.316906	5.316906	No
Yes		Hb_adm<110		21.41124	21.41124	7.551528	7.551528	No
Yes		Hb_adm<110		2.907346		12.94522		Yes
Yes	No	Hb_adm<1	Hb_exit<1:	62.68419	62.68419	7.94027	7.94027	No
No	No	Hb_adm>=	Hb_exit<1:	4.23617	4.23617	12.19709	12.19709	Yes
Yes	Yes	Hb_adm<1	Hb_exit<1:	36.08276	36.08276	9.734613	9.734613	No
Yes	Yes	Hb_adm<1	Hb_exit<1:	2.757733	2.757733	23.96451	23.96451	Yes
No	No	Hb_adm>=	Hb_exit<1:	18.12351	18.12351	5.684165	5.684165	No

Yes		Hb_adm<110						
Yes	Yes	Hb_adm<1	Hb_exit<1	15.60551	17.29304	6.200048	7.292017	No
Yes		Hb_adm<110		28.5245	28.5245	6.413899	6.413899	No
Yes	Yes	Hb_adm<1	Hb_exit<1	2.929918	2.929918	13.81771	13.81771	Yes
Yes		Hb_adm<110		5.50279	5.50279	9.000676	9.000676	Yes
No		Hb_adm>=110		8.258509	8.258509	6.89145	6.89145	Yes
Yes	Yes	Hb_adm<1	Hb_exit<1	3.799683	3.799683	6.466385	6.466385	Yes
Yes	No	Hb_adm<1	Hb_exit<1	12.72441	31.22022	11.40932	4.590272	No
Yes		Hb_adm<110		10.15	10.15	9.925	9.925	Yes
Yes	Yes	Hb_adm<1	Hb_exit<1	9.544504	9.544504	11.37775	11.37775	Yes
Yes	Yes	Hb_adm<1	Hb_exit<1	6.898118	6.898118	6.661369	6.661369	Yes
Yes	No	Hb_adm<1	Hb_exit<1	7.192407	7.192407	11.33083	11.33083	Yes
Yes	Yes	Hb_adm<1	Hb_exit<1	7.56458	7.56458	10.88737	10.88737	Yes
Yes	Yes	Hb_adm<1	Hb_exit<1	63.56378	63.56378	6.635651	6.635651	No
Yes	No	Hb_adm<1	Hb_exit<1	40.38698	40.38698	6.877063	6.877063	No
Yes	Yes	Hb_adm<1	Hb_exit<1	10.66224	10.66224	6.996707	6.996707	Yes
Yes	Yes	Hb_adm<1	Hb_exit<1	25.61656	25.61656	7.544404	7.544404	No
Yes		Hb_adm<110		29.68671	29.68671	14.28952	14.28952	No
Yes	Yes	Hb_adm<1	Hb_exit<1	2.846589	2.846589	7.412137	7.412137	Yes
Yes	Yes	Hb_adm<1	Hb_exit<1	25.83179	24.81943	8.703975	13.44866	No
Yes	Yes	Hb_adm<1	Hb_exit<1	19.55729	19.55729	4.679483	4.679483	No
Yes	Yes	Hb_adm<1	Hb_exit<1	67.27631	67.27631	6.35493	6.35493	No
Yes	Yes	Hb_adm<1	Hb_exit<1	21.96263	21.96263	5.07135	5.07135	No
Yes	No	Hb_adm<1	Hb_exit<1	8.734038	8.734038	11.60359	11.60359	Yes
Yes		Hb_adm<110		6.783385	6.783385	8.414738	8.414738	Yes
No	No	Hb_adm>=	Hb_exit<1	13.99904	13.99904	6.884331	6.884331	No
Yes	Yes	Hb_adm<1	Hb_exit<1	59.25413	59.25413	7.353113	7.353113	No
Yes	No	Hb_adm<1	Hb_exit<1	39.88054	39.88054	9.778976	9.778976	No
Yes	Yes	Hb_adm<1	Hb_exit<1	131.3148	131.3148	13.21279	13.21279	No
Yes		Hb_adm<110		56.5753	56.5753	3.960511	3.960511	No
Yes	No	Hb_adm<1	Hb_exit<1	10.88601	10.88601	6.218234	6.218234	Yes
Yes	Yes	Hb_adm<1	Hb_exit<1	26.58088	54.07029	10.21478	17.13976	No
Yes	Yes	Hb_adm<1	Hb_exit<1	13.875	16.13763	12.13	19.77573	No
Yes		Hb_adm<110		34.16642	34.16642	9.121536	9.121536	No
Yes	No	Hb_adm<1	Hb_exit<1	45.79836	45.79836	15.37128	15.37128	No
Yes	Yes	Hb_adm<1	Hb_exit<1	44.67689	44.67689	17.19816	17.19816	No
Yes	Yes	Hb_adm<1	Hb_exit<1	120.3014	120.3014	9.213929	9.213929	No
Yes	Yes	Hb_adm<1	Hb_exit<1	39.45728	39.45728	5.96339	5.96339	No
Yes		Hb_adm<110						
Yes	Yes	Hb_adm<1	Hb_exit<1	47.36367	47.36367	4.05362	4.05362	No
Yes	Yes	Hb_adm<1	Hb_exit<1	40.62204	12.08337	3.990657	12.71194	No
Yes	Yes	Hb_adm<1	Hb_exit<1	6.277224	6.277224	15.4526	15.4526	Yes
Yes	No	Hb_adm<1	Hb_exit<1	4.06229	4.06229	20.19216	20.19216	Yes
Yes		Hb_adm<110		17.20276	17.20276	12.14774	12.14774	No
Yes	Yes	Hb_adm<1	Hb_exit<1	26.70267	26.70267	11.33774	11.33774	No
Yes	Yes	Hb_adm<1	Hb_exit<1	12.16294	12.16294	16.01091	16.01091	No
Yes	No	Hb_adm<1	Hb_exit<1	6.590299	6.590299	8.827252	8.827252	Yes
No	No	Hb_adm>=	Hb_exit<1	12.06595	12.06595	7.352772	7.352772	No
Yes	No	Hb_adm<1	Hb_exit<1	15.036	15.036	8.378413	8.378413	No
Yes	Yes	Hb_adm<1	Hb_exit<1	9.216748	9.216748	15.3339	15.3339	Yes
Yes	Yes	Hb_adm<1	Hb_exit<1	11.59753	11.59753	9.282699	9.282699	Yes

Yes	Yes	Hb_adm<1	Hb_exit<1:	2.154463	2.154463	13.01504	13.01504	Yes
Yes		Hb_adm<110						
Yes	Yes	Hb_adm<1	Hb_exit<1:	7.180042	7.180042	14.71863	14.71863	Yes
No		Hb_adm>=110		16.09123	16.09123	5.438533	5.438533	No
No	No	Hb_adm>=	Hb_exit<1:	11.43818	11.43818	5.339667	5.339667	Yes
Yes		Hb_adm<110		7.296079	7.296079	9.022989	9.022989	Yes
Yes	No	Hb_adm<1	Hb_exit<1:	11.40962	11.40962	6.782838	6.782838	Yes
No	No	Hb_adm>=	Hb_exit<1:	17.88906	17.88906	6.758044	6.758044	No
Yes	Yes	Hb_adm<1	Hb_exit<1:	11.78832	11.78832	14.00676	14.00676	Yes
Yes	No	Hb_adm<1	Hb_exit<1:	23.84011	23.84011	7.395219	7.395219	No
Yes	No	Hb_adm<1	Hb_exit<1:	23.1	23.1	6.52	6.52	No
Yes	Yes	Hb_adm<1	Hb_exit<1:	115.2658	115.2658	32.76662	32.76662	No
Yes		Hb_adm<110		4.315121	3.421942	15.97007	25.07797	Yes
Yes		Hb_adm<110		18.98934	18.98934	12.76827	12.76827	No
Yes	No	Hb_adm<1	Hb_exit<1:	11.17942	11.17942	8.130709	8.130709	Yes
Yes	Yes	Hb_adm<1	Hb_exit<1:	8.586573	8.586573	11.2481	11.2481	Yes
Yes	Yes	Hb_adm<1	Hb_exit<1:	8.164405	8.164405	12.19903	12.19903	Yes
Yes	No	Hb_adm<1	Hb_exit<1:	3.175754	3.175754	15.12436	15.12436	Yes
Yes	No	Hb_adm<1	Hb_exit<1:	2.962584	2.962584	9.394828	9.394828	Yes
Yes		Hb_adm<110		2.803036		12.77148		Yes
Yes	No	Hb_adm<1	Hb_exit<110					
Yes	Yes	Hb_adm<1	Hb_exit<1:	8.215806	8.215806	6.783897	6.783897	Yes
Yes	Yes	Hb_adm<1	Hb_exit<1:	16.60878	16.60878	5.620897	5.620897	No
Yes		Hb_adm<110		30.13771	30.13771	12.36811	12.36811	No
Yes	No	Hb_adm<1	Hb_exit<1:	44.7083	46.20144	5.661015	4.366195	No
Yes	Yes	Hb_adm<1	Hb_exit<1:	5.455637	5.455637	8.383941	8.383941	Yes
Yes	Yes	Hb_adm<1	Hb_exit<1:	11.29381	11.29381	7.93656	7.93656	Yes
Yes	No	Hb_adm<1	Hb_exit<1:	25.14	25.14	5.255	5.255	No
Yes	Yes	Hb_adm<1	Hb_exit<1:	12.97633	12.97633	8.18974	8.18974	No
Yes		Hb_adm<110		18.89979	18.89979	4.629025	4.629025	No
Yes	No	Hb_adm<1	Hb_exit<1:	7.359516	7.359516	20.47434	20.47434	Yes
Yes		Hb_adm<110		72.91389	72.91389	7.990621	7.990621	No
Yes	Yes	Hb_adm<1	Hb_exit<1:	18.07667	18.07667	31.94418	31.94418	No
Yes	Yes	Hb_adm<1	Hb_exit<1:	6.62251	6.62251	18.48795	18.48795	Yes
Yes	Yes	Hb_adm<1	Hb_exit<1:	2.67376	2.67376	31.05915	31.05915	Yes
No	No	Hb_adm>=	Hb_exit<1:	13.46611	13.46611	6.403712	6.403712	No
Yes	No	Hb_adm<1	Hb_exit<1:	74.34249	74.34249	8.529502	8.529502	No
Yes	No	Hb_adm<1	Hb_exit<1:	15.28989	15.28989	11.01782	11.01782	No
Yes	No	Hb_adm<1	Hb_exit<1:	15.04852	15.04852	6.618623	6.618623	No
No		Hb_adm>=110		11.51347	11.51347	6.585459	6.585459	Yes
Yes		Hb_adm<110		7.687373	7.687373	14.05284	14.05284	Yes
No	Yes	Hb_adm>=	Hb_exit<1:	1.068054	1.068054	8.958161	8.958161	Yes
Yes	Yes	Hb_adm<1	Hb_exit<1:	1.312738	1.312738	12.65685	12.65685	Yes
No		Hb_adm>=110		12.72621	12.72621	4.902978	4.902978	No
Yes	Yes	Hb_adm<1	Hb_exit<1:	42.77166	42.77166	6.975971	6.975971	No
Yes		Hb_adm<110		49.91897	49.91897	3.493963	3.493963	No
No	No	Hb_adm>=	Hb_exit<1:	28.56024	28.56024	8.368783	8.368783	No
Yes	Yes	Hb_adm<1	Hb_exit<1:	11.2086	11.2086	11.13739	11.13739	Yes
Yes	Yes	Hb_adm<1	Hb_exit<1:	8.512976	8.512976	9.268195	9.268195	Yes
Yes	No	Hb_adm<1	Hb_exit<1:	4.027572	4.027572	23.80903	23.80903	Yes
Yes	Yes	Hb_adm<1	Hb_exit<1:	2.801419	2.801419	11.8549	11.8549	Yes

Yes		Hb_adm<110		9.370198	9.370198	22.25112	22.25112	Yes
Yes	No	Hb_adm<1	Hb_exit<1:	86.12154	86.12154	13.97082	13.97082	No
Yes	No	Hb_adm<1	Hb_exit<1:	50.19528	50.19528	16.45878	16.45878	No
Yes	Yes	Hb_adm<1	Hb_exit<1:	10.75734	58.37096	8.028772	7.712881	Yes
Yes		Hb_adm<110						
Yes	Yes	Hb_adm<1	Hb_exit<1:	4.685699	1.388488	21.42971	23.74126	Yes
Yes		Hb_adm<110		19.14142	19.14142	11.62702	11.62702	No
Yes		Hb_adm<110		22.61632	22.61632	23.82333	23.82333	No
Yes	Yes	Hb_adm<1	Hb_exit<1:	2.023914	2.023914	13.0804	13.0804	Yes
Yes		Hb_adm<110		15.12897	15.12897	9.323971	9.323971	No
Yes	No	Hb_adm<1	Hb_exit<1:	14.14781	14.14781	10.88179	10.88179	No
Yes	Yes	Hb_adm<1	Hb_exit<1:	78.39	78.39	7.31	7.31	No
No	Yes	Hb_adm>=	Hb_exit<1:	15.17916	15.17916	12.73901	12.73901	No
Yes	No	Hb_adm<1	Hb_exit<1:	14.73889	14.73889	6.232929	6.232929	No
Yes	No	Hb_adm<1	Hb_exit<1:	5.987367	5.987367	6.948984	6.948984	Yes
No		Hb_adm>=110		4.193367	4.193367	4.827688	4.827688	Yes
Yes	Yes	Hb_adm<1	Hb_exit<1:	6.658235	6.658235	30.20073	30.20073	Yes
Yes	Yes	Hb_adm<1	Hb_exit<1:	6.862751	6.862751	9.306171	9.306171	Yes
Yes	No	Hb_adm<1	Hb_exit<1:	34.06684	34.06684	5.951294	5.951294	No
Yes	No	Hb_adm<1	Hb_exit<1:	3.106958	3.106958	17.02787	17.02787	Yes
No	Yes	Hb_adm>=	Hb_exit<1:	21.60058	21.60058	8.338347	8.338347	No
Yes		Hb_adm<110		12.29881	12.29881	9.485136	9.485136	No
Yes	No	Hb_adm<1	Hb_exit<1:	3.155431	3.155431	8.170212	8.170212	Yes
No	Yes	Hb_adm>=	Hb_exit<1:	45.46069	45.46069	5.578773	5.578773	No
Yes	No	Hb_adm<1	Hb_exit<1:	3.797804	3.797804	12.80215	12.80215	Yes
No	Yes	Hb_adm>=	Hb_exit<1:	11.379	11.379	4.517539	4.517539	Yes
Yes	Yes	Hb_adm<1	Hb_exit<1:	6.052678	6.052678	10.80152	10.80152	Yes
Yes	Yes	Hb_adm<1	Hb_exit<1:	29.61984	29.61984	12.79434	12.79434	No
Yes	Yes	Hb_adm<1	Hb_exit<1:	2.275219	2.275219	18.06616	18.06616	Yes
No	No	Hb_adm>=	Hb_exit<1:	30.40866	30.40866	3.741549	3.741549	No
Yes	Yes	Hb_adm<1	Hb_exit<1:	8.148695	8.148695	18.23896	18.23896	Yes
Yes		Hb_adm<110		26.38707	26.38707	6.957086	6.957086	No
Yes		Hb_adm<110		18.35985	18.35985	9.551783	9.551783	No
Yes	Yes	Hb_adm<1	Hb_exit<1:	44.73132	44.73132	5.300325	5.300325	No
Yes	No	Hb_adm<1	Hb_exit<1:	47.0815	47.0815	12.46624	12.46624	No
Yes	No	Hb_adm<1	Hb_exit<1:	91.418	91.418	6.459143	6.459143	No
Yes	Yes	Hb_adm<1	Hb_exit<110					
Yes	Yes	Hb_adm<1	Hb_exit<1:	36.40354	36.40354	11.36234	11.36234	No
Yes	No	Hb_adm<1	Hb_exit<1:	28.63799	28.63799	6.729549	6.729549	No
No	No	Hb_adm>=	Hb_exit<1:	12.77166	12.77166	7.743733	7.743733	No
Yes	Yes	Hb_adm<1	Hb_exit<1:	13.82753	13.82753	8.305409	8.305409	No
Yes		Hb_adm<110		38.52611	38.52611	7.623625	7.623625	No
Yes		Hb_adm<110						
Yes		Hb_adm<110		76.73425	76.73425	16.02647	16.02647	No
No		Hb_adm>=110		15.77314	15.77314	7.341436	7.341436	No
Yes	Yes	Hb_adm<1	Hb_exit<1:	3.435875	2.891082	18.3311	18.40935	Yes
Yes		Hb_adm<110		38.26547	38.26547	4.483539	4.483539	No
No	No	Hb_adm>=	Hb_exit<1:	21.60033	21.60033	5.094954	5.094954	No
Yes	Yes	Hb_adm<1	Hb_exit<1:	32.30785	32.30785	8.665586	8.665586	No
Yes		Hb_adm<110		11.37225	11.37225	11.32178	11.32178	Yes
Yes		Hb_adm<110						

Yes	No	Hb_adm<1	Hb_exit<1:	27.229	27.229	7.104725	7.104725	No
Yes	No	Hb_adm<1	Hb_exit<1:	89.60122	89.60122	5.469371	5.469371	No
Yes	Yes	Hb_adm<1	Hb_exit<1:	15.13886	15.13886	7.588828	7.588828	No
Yes	Yes	Hb_adm<1	Hb_exit<1:	4.538416	4.538416	11.35881	11.35881	Yes
Yes	No	Hb_adm<1	Hb_exit<1:	59.05851	59.05851	7.139038	7.139038	No
No	No	Hb_adm>=	Hb_exit<1:	5.121114	5.121114	6.705464	6.705464	Yes
Yes	Yes	Hb_adm<1	Hb_exit<1:	24.44835	24.44835	4.378776	4.378776	No
Yes	No	Hb_adm<1	Hb_exit<1:	15.92971	8.44	8.545882	12.16	No
No	No	Hb_adm>=	Hb_exit<1:	29.56082	29.56082	7.106675	7.106675	No
Yes	Yes	Hb_adm<1	Hb_exit<1:	68.92233	68.92233	12.7567	12.7567	No
No	Yes	Hb_adm>=	Hb_exit<1:	5.314779	5.314779	10.73483	10.73483	Yes
Yes	Yes	Hb_adm<1	Hb_exit<1:	12.71581	16.5084	6.503861	6.703216	No
Yes	Yes	Hb_adm<1	Hb_exit<1:	29.13689	29.13689	9.544619	9.544619	No
Yes		Hb_adm<110		51.90923		14.12027		No
Yes	Yes	Hb_adm<1	Hb_exit<1:	31.14164	31.14164	10.20998	10.20998	No
No		Hb_adm>=110		16.38311	16.38311	8.325785	8.325785	No
Yes	Yes	Hb_adm<1	Hb_exit<1:	43.816	43.816	12.20384	12.20384	No
Yes		Hb_adm<110		57.66314	57.66314	7.838284	7.838284	No
Yes	No	Hb_adm<1	Hb_exit<1:	5.095984	5.095984	7.69367	7.69367	Yes
Yes		Hb_adm<110		48.87785	48.87785	8.573274	8.573274	No
No		Hb_adm>=110		9.44267	9.44267	6.282415	6.282415	Yes
Yes	No	Hb_adm<1	Hb_exit<1:	9.275627	9.275627	10.86034	10.86034	Yes
Yes	No	Hb_adm<1	Hb_exit<1:	25.40223	25.40223	7.824564	7.824564	No
Yes	No	Hb_adm<1	Hb_exit<1:	34.66	32.66368	7.39	7.650901	No
Yes	Yes	Hb_adm<1	Hb_exit<1:	49.08053	49.08053	5.400255	5.400255	No
Yes		Hb_adm<110		26.20693	26.20693	6.755222	6.755222	No
No	No	Hb_adm>=	Hb_exit<1:	32.86445	32.86445	6.705406	6.705406	No
Yes	No	Hb_adm<1	Hb_exit<1:	6.491965	6.491965	8.216761	8.216761	Yes
Yes	No	Hb_adm<1	Hb_exit<1:	17.96774	17.96774	8.52329	8.52329	No
Yes	Yes	Hb_adm<1	Hb_exit<1:	22.25183	11.32686	8.557108	11.54916	No
Yes	No	Hb_adm<1	Hb_exit<1:	2.737048	2.737048	11.56711	11.56711	Yes
Yes	No	Hb_adm<1	Hb_exit<1:	8.862556	8.862556	8.259611	8.259611	Yes
Yes	No	Hb_adm<1	Hb_exit<1:	3.049374	3.049374	6.351679	6.351679	Yes
Yes	Yes	Hb_adm<1	Hb_exit<1:	15.26387	15.26387	4.935455	4.935455	No
Yes	No	Hb_adm<1	Hb_exit<1:	28.36864	36.10263	7.199741	6.888201	No
No	No	Hb_adm>=	Hb_exit<1:	12.75058	12.75058	8.069489	8.069489	No
Yes		Hb_adm<110		3.140783	3.140783	16.81607	16.81607	Yes
Yes	Yes	Hb_adm<1	Hb_exit<110					
Yes		Hb_adm<110		14.85936	14.85936	7.53099	7.53099	No
No	No	Hb_adm>=	Hb_exit<1:	12.08189	12.08189	6.674014	6.674014	No
Yes	No	Hb_adm<1	Hb_exit<1:	81.39815	81.39815	3.939136	3.939136	No
No	Yes	Hb_adm>=	Hb_exit<1:	13.09624	13.09624	4.192906	4.192906	No
No		Hb_adm>=110		35.23499	35.23499	7.882332	7.882332	No
No	No	Hb_adm>=	Hb_exit<1:	26.30927	47.85688	5.113919	4.636503	No
Yes	No	Hb_adm<1	Hb_exit<1:	7.300114	7.300114	8.307593	8.307593	Yes
Yes		Hb_adm<110		5.388951	5.388951	10.60621	10.60621	Yes
Yes		Hb_adm<110		17.10118	17.10118	6.255856	6.255856	No
Yes		Hb_adm<110						
Yes		Hb_adm<110		3.866131	3.866131	5.519876	5.519876	Yes
No	No	Hb_adm>=	Hb_exit<1:	22.38295	22.38295	4.913402	4.913402	No
No		Hb_adm>=110		5.959787	5.959787	5.260226	5.260226	Yes

No	No	Hb_adm>=	Hb_exit<1:	13.17695	13.17695	5.48568	5.48568	No
Yes		Hb_adm<110		7.007482	7.007482	5.564091	5.564091	Yes
No		Hb_adm>=110		11.28432	11.28432	6.382707	6.382707	Yes
Yes		Hb_adm<110		5.139231	5.139231	18.24934	18.24934	Yes
Yes	Yes	Hb_adm<1	Hb_exit<1:	46.56977	46.56977	7.035248	7.035248	No
No		Hb_adm>=110						
No	Yes	Hb_adm>=	Hb_exit<1:	14.69269	14.69269	6.241387	6.241387	No
Yes		Hb_adm<110		16.21489	3.14121	6.041611	7.637252	No
Yes	Yes	Hb_adm<1	Hb_exit<1:	4.47094	4.47094	6.495558	6.495558	Yes
Yes	Yes	Hb_adm<1	Hb_exit<1:	1.79438	1.79438	18.57123	18.57123	Yes
Yes	Yes	Hb_adm<1	Hb_exit<1:	36.8826	36.8826	9.628541	9.628541	No
Yes		Hb_adm<110		6.760108	6.760108	8.456922	8.456922	Yes
No		Hb_adm>=110		21.45179	21.45179	1.232258	1.232258	No
Yes	No	Hb_adm<1	Hb_exit<1:	8.793067	8.793067	6.770532	6.770532	Yes
No	No	Hb_adm>=	Hb_exit<1:	71.3144	71.3144	5.195004	5.195004	No
No	Yes	Hb_adm>=	Hb_exit<1:	18.68754	18.68754	5.911038	5.911038	No
Yes		Hb_adm<110		16.40644	16.40644	22.37699	22.37699	No
Yes	Yes	Hb_adm<1	Hb_exit<1:	16.69844	16.69844	4.850794	4.850794	No
Yes	No	Hb_adm<1	Hb_exit<1:	9.455244	9.455244	21.59408	21.59408	Yes
No	No	Hb_adm>=	Hb_exit<1:	30.96	30.96	8.78	8.78	No
No	No	Hb_adm>=	Hb_exit<1:	0.767552	0.767552	16.95782	16.95782	Yes
Yes		Hb_adm<110		65.23973	65.23973	9.926439	9.926439	No
No		Hb_adm>=110		2.441186	2.441186	31.05855	31.05855	Yes
Yes	No	Hb_adm<1	Hb_exit<1:	15.09921	15.09921	5.479455	5.479455	No
Yes		Hb_adm<110		9.504754	9.504754	7.390668	7.390668	Yes
No		Hb_adm>=110		30.09414	30.09414	5.879032	5.879032	No
No	No	Hb_adm>=	Hb_exit<1:	10.93379	10.93379	11.51536	11.51536	Yes
Yes	Yes	Hb_adm<1	Hb_exit<1:	5.853856	5.853856	8.064684	8.064684	Yes
Yes	No	Hb_adm<1	Hb_exit<1:	37.65031	37.65031	11.95255	11.95255	No
Yes	Yes	Hb_adm<1	Hb_exit<1:	101.7074	18.48606	13.65934	16.95254	No
No	No	Hb_adm>=	Hb_exit<1:	9.318997	9.318997	8.655619	8.655619	Yes
No		Hb_adm>=110		9.724017	9.724017	7.819748	7.819748	Yes
No	Yes	Hb_adm>=	Hb_exit<1:	22.38146	22.38146	7.54675	7.54675	No
Yes		Hb_adm<110		12.81831		11.02617		No
Yes		Hb_adm<110		3.709611	3.709611	13.20773	13.20773	Yes
Yes		Hb_adm<110		34.43609	23.89203	12.90073	18.66779	No
Yes	No	Hb_adm<1	Hb_exit<1:	20.40526	20.40526	5.732276	5.732276	No
Yes	Yes	Hb_adm<1	Hb_exit<1:	19.8659	19.8659	2.895685	2.895685	No
Yes		Hb_adm<110		45.56186	45.56186	8.450797	8.450797	No
Yes		Hb_adm<110		26.76287	26.76287	8.186337	8.186337	No
Yes		Hb_adm<110		112.3486	112.3486	2.933003	2.933003	No
Yes	No	Hb_adm<1	Hb_exit<1:	11.31232	13.26237	9.439209	6.556453	Yes
No		Hb_adm>=110		49.16079	49.16079	4.15277	4.15277	No
No		Hb_adm>=110		10.71468	10.71468	7.376924	7.376924	Yes
No		Hb_adm>=110		7.050208	7.050208	4.818912	4.818912	Yes
Yes		Hb_adm<110		9.305854	9.305854	7.78201	7.78201	Yes
No	No	Hb_adm>=	Hb_exit<1:	4.860955	4.860955	8.926075	8.926075	Yes
No	No	Hb_adm>=	Hb_exit<1:	21.12	21.12	5.41	5.41	No
Yes	Yes	Hb_adm<1	Hb_exit<1:	8.431677	8.431677	6.873292	6.873292	Yes
Yes		Hb_adm<110		11.86817	11.86817	7.701907	7.701907	Yes
No		Hb_adm>=110		19.85606	19.85606	6.484838	6.484838	No

Yes	Yes	Hb_adm<1	Hb_exit<1:	9.384598	9.384598	6.961774	6.961774	Yes
Yes		Hb_adm<110		9.371788	9.371788	9.802356	9.802356	Yes
No		Hb_adm>=110		22.13555	22.13555	5.43294	5.43294	No
No	Yes	Hb_adm>=	Hb_exit<110					
No	No	Hb_adm>=	Hb_exit<1:	7.24	7.24	10.07	10.07	Yes
Yes		Hb_adm<110		46.29002	46.29002	4.513955	4.513955	No
Yes	No	Hb_adm<1	Hb_exit<1:	4.407326	4.407326	9.448406	9.448406	Yes
Yes		Hb_adm<110		11.28196	11.28196	7.877964	7.877964	Yes
Yes		Hb_adm<110						
Yes		Hb_adm<110		10.78623	10.78623	5.904344	5.904344	Yes
Yes	Yes	Hb_adm<1	Hb_exit<1:	23.62624	23.62624	1.863127	1.863127	No
No		Hb_adm>=110		4.78	4.78	8.1	8.1	Yes
No		Hb_adm>=110		12.76533	12.76533	5.871038	5.871038	No
Yes		Hb_adm<110		2.706995	2.706995	10.48439	10.48439	Yes
No	Yes	Hb_adm>=	Hb_exit<1:	59.58704	43.68402	6.847526	5.653744	No
Yes		Hb_adm<110		10.45423	10.45423	5.851913	5.851913	Yes
Yes	Yes	Hb_adm<1	Hb_exit<1:	4.228427	4.228427	6.755425	6.755425	Yes
Yes		Hb_adm<110		4.994087	4.994087	5.932545	5.932545	Yes
Yes	No	Hb_adm<1	Hb_exit<1:	19.10114	19.10114	11.25835	11.25835	No
Yes		Hb_adm<110		8.123296	8.123296	8.499944	8.499944	Yes
No		Hb_adm>=110		13.94108	13.94108	5.38669	5.38669	No
Yes		Hb_adm<110		2.103258	2.103258	14.7247	14.7247	Yes
Yes		Hb_adm<110						
No	No	Hb_adm>=	Hb_exit<1:	16.04118	16.04118	1.598148	1.598148	No
Yes	Yes	Hb_adm<1	Hb_exit<1:	25.77487	25.77487	10.48421	10.48421	No
Yes		Hb_adm<110						
Yes	No	Hb_adm<1	Hb_exit<1:	0.480397	0.480397	26.93359	26.93359	Yes
No	Yes	Hb_adm>=	Hb_exit<1:	9.009884	9.009884	10.97538	10.97538	Yes
No	Yes	Hb_adm>=	Hb_exit<1:	32.25444	32.25444	4.250556	4.250556	No
Yes	Yes	Hb_adm<1	Hb_exit<1:	44.44526	44.44526	7.502357	7.502357	No
Yes	Yes	Hb_adm<1	Hb_exit<1:	53.57029	53.57029	7.027424	7.027424	No
Yes	Yes	Hb_adm<1	Hb_exit<1:	14.02764	14.02764	24.60758	24.60758	No
Yes	Yes	Hb_adm<1	Hb_exit<110					
No	No	Hb_adm>=	Hb_exit<1:	7.423246	7.423246	7.345045	7.345045	Yes
No	No	Hb_adm>=	Hb_exit<1:	83.32157	83.32157	4.906793	4.906793	No
Yes	Yes	Hb_adm<1	Hb_exit<1:	3.19328	3.19328	17.63559	17.63559	Yes
Yes	No	Hb_adm<1	Hb_exit<1:	9.043604	4.774701	7.311268	2.625756	Yes
Yes		Hb_adm<110		7.433407	7.433407	9.825954	9.825954	Yes
No	No	Hb_adm>=	Hb_exit<1:	16.49	16.49	6.59	6.59	No
No	Yes	Hb_adm>=	Hb_exit<1:	19.49642	13.13813	8.253569	4.053117	No
Yes		Hb_adm<110		14.26665	14.26665	7.607702	7.607702	No
Yes	No	Hb_adm<1	Hb_exit<1:	42.7757	42.7757	13.21931	13.21931	No
Yes	No	Hb_adm<1	Hb_exit<1:	3.045629	3.045629	10.47628	10.47628	Yes
No	No	Hb_adm>=	Hb_exit<1:	8.417609	8.417609	7.90254	7.90254	Yes
Yes	Yes	Hb_adm<1	Hb_exit<1:	13.89474	13.89474	10.7918	10.7918	No
Yes	No	Hb_adm<1	Hb_exit<1:	3.78	3.05	7.4	23.57	Yes
Yes	Yes	Hb_adm<1	Hb_exit<1:	22.9066	22.9066	8.563769	8.563769	No
No		Hb_adm>=110		78.26492	78.26492	7.286365	7.286365	No
Yes		Hb_adm<110		1.29312	1.29312	17.47558	17.47558	Yes
Yes		Hb_adm<110		45.32827	45.32827	6.229585	6.229585	No
Yes	Yes	Hb_adm<1	Hb_exit<1:	15.97906	15.97906	11.89218	11.89218	No

No	Yes	Hb_adm>=	Hb_exit<1:	13.7437	13.7437	10.22529	10.22529	No
No		Hb_adm>=110		23.6023	23.6023	14.44735	14.44735	No
Yes		Hb_adm<110		6.77202	6.77202	13.19526	13.19526	Yes
Yes	Yes	Hb_adm<1	Hb_exit<1:	51.36712	31.97257	4.86747	0.61636	No
Yes		Hb_adm<110		6.07837	6.07837	12.4095	12.4095	Yes
Yes		Hb_adm<110		51.18407	51.18407	24.76965	24.76965	No
Yes	Yes	Hb_adm<1	Hb_exit<1:	16.20174	16.20174	1.419713	1.419713	No
Yes		Hb_adm<110		40.43849	40.43849	5.188506	5.188506	No
Yes	No	Hb_adm<1	Hb_exit<1:	28.13117	25.95469	16.54248	5.568536	No
Yes		Hb_adm<110		36.02554	36.02554	11.04356	11.04356	No
Yes	Yes	Hb_adm<1	Hb_exit<1:	31.7271	23.00523	9.143296	13.11831	No
Yes	Yes	Hb_adm<1	Hb_exit<1:	28.39473	28.39473	30.80299	30.80299	No
Yes		Hb_adm<110						
Yes	Yes	Hb_adm<1	Hb_exit<1:	20.99512	20.99512	12.60877	12.60877	No
Yes		Hb_adm<110		11.50931	11.50931	11.03364	11.03364	Yes
Yes	Yes	Hb_adm<1	Hb_exit<1:	11.73598	11.73598	29.55452	29.55452	Yes
Yes		Hb_adm<110		4.745962	4.745962	12.26052	12.26052	Yes
No		Hb_adm>=110		27.93367	27.93367	2.678669	2.678669	No
Yes		Hb_adm<110		1.890298		9.63095		Yes
Yes		Hb_adm<110		17.9743		5.477446		No
Yes		Hb_adm<110		63.67495	63.67495	11.96162	11.96162	No
Yes	No	Hb_adm<1	Hb_exit<1:	5.656906	5.656906	16.77161	16.77161	Yes
Yes	Yes	Hb_adm<1	Hb_exit<1:	13.13815	13.13815	5.597199	5.597199	No
Yes	Yes	Hb_adm<1	Hb_exit<1:	25.675	25.675	1.145	1.145	No
Yes		Hb_adm<110		129.3426	129.3426	6.447489	6.447489	No
Yes	Yes	Hb_adm<1	Hb_exit<1:	0.554383	0.554383	29.63294	29.63294	Yes
Yes	Yes	Hb_adm<1	Hb_exit<1:	13.95672	30.97527	10.49624	15.73289	No
Yes	No	Hb_adm<1	Hb_exit<1:	3.94552	12.41	34.69958	14.605	Yes
Yes	No	Hb_adm<1	Hb_exit<1:	6.170087	17.48974	7.082283	4.423523	Yes
Yes	Yes	Hb_adm<1	Hb_exit<1:	31.741	2.396762	13.3794	31.14529	No
Yes	Yes	Hb_adm<1	Hb_exit<1:	1.319366	1.319366	30.4681	30.4681	Yes
Yes	No	Hb_adm<1	Hb_exit<1:	7.991458	7.991458	4.295696	4.295696	Yes
Yes		Hb_adm<110		15.18962	15.18962	10.21596	10.21596	No
Yes	No	Hb_adm<1	Hb_exit<1:	44.93239	19.99333	6.612932	5.54226	No
Yes	No	Hb_adm<1	Hb_exit<1:	65.41692	65.41692	4.42119	4.42119	No
Yes	Yes	Hb_adm<1	Hb_exit<1:	5.974348	5.974348	12.68408	12.68408	Yes
Yes		Hb_adm<110		5.719196	5.719196	18.08063	18.08063	Yes
Yes		Hb_adm<110						
Yes		Hb_adm<110		2.278963	2.278963	25.82495	25.82495	Yes
Yes	Yes	Hb_adm<1	Hb_exit<1:	11.16102	11.16102	0.951734	0.951734	Yes
Yes	Yes	Hb_adm<1	Hb_exit<1:	2.379137	2.379137	21.43763	21.43763	Yes
Yes	Yes	Hb_adm<1	Hb_exit<1:	6.499218	6.499218	11.14366	11.14366	Yes
Yes	No	Hb_adm<1	Hb_exit<1:	9.6066	9.6066	12.42256	12.42256	Yes
Yes		Hb_adm<110		64.52378	64.52378	8.332484	8.332484	No
No	Yes	Hb_adm>=	Hb_exit<1:	23.90939	23.90939	5.624327	5.624327	No
Yes	No	Hb_adm<1	Hb_exit<1:	7.840074	27.66265	14.02249	12.6454	Yes
Yes	No	Hb_adm<1	Hb_exit<1:	12.68224	1.987586	10.338	31.41145	No
Yes		Hb_adm<110						
Yes		Hb_adm<110		4.784619	4.784619	14.09891	14.09891	Yes
Yes		Hb_adm<110		6.308842	6.308842	20.93789	20.93789	Yes
Yes		Hb_adm<110		10.94471	10.94471	16.10609	16.10609	Yes

No		Hb_adm>=110						
Yes		Hb_adm<110		56.07439	56.07439	5.071014	5.071014	No
No		Hb_adm>=110		9.960198	9.960198	8.626915	8.626915	Yes
Yes	No	Hb_adm<1	Hb_exit<1:	4.946059	4.946059	19.42273	19.42273	Yes
No	No	Hb_adm>=	Hb_exit<1:	5.797295	5.797295	11.15701	11.15701	Yes
Yes	Yes	Hb_adm<1	Hb_exit<1:	33.99574	33.99574	4.645562	4.645562	No
Yes		Hb_adm<110		11.77911	11.77911	6.872361	6.872361	Yes
Yes	Yes	Hb_adm<1	Hb_exit<1:	20.70594	20.70594	18.56123	18.56123	No
No	No	Hb_adm>=	Hb_exit<1:	19.92119	19.92119	1.134342	1.134342	No
No		Hb_adm>=110		19.0053	19.0053	5.069802	5.069802	No
Yes	Yes	Hb_adm<1	Hb_exit<1:	3.64963	3.64963	28.41727	28.41727	Yes
Yes		Hb_adm<110		21.2935	21.2935	5.446564	5.446564	No
No	No	Hb_adm>=	Hb_exit<1:	18.68476	18.68476	9.753433	9.753433	No
No	Yes	Hb_adm>=	Hb_exit<1:	3.700166	3.700166	8.104896	8.104896	Yes
Yes	Yes	Hb_adm<1	Hb_exit<1:	2.67089	2.67089	17.60479	17.60479	Yes
Yes	Yes	Hb_adm<1	Hb_exit<1:	19.48472	19.48472	14.30211	14.30211	No
Yes		Hb_adm<110		2.342537	2.342537	10.859	10.859	Yes
Yes	Yes	Hb_adm<1	Hb_exit<1:	2.423512	0.794288	27.5874	36.16184	Yes
Yes		Hb_adm<110		6.568378	6.568378	7.332437	7.332437	Yes
Yes	Yes	Hb_adm<1	Hb_exit<1:	5.662785	5.662785	13.13859	13.13859	Yes
Yes	Yes	Hb_adm<1	Hb_exit<1:	13.31221	13.31221	10.88723	10.88723	No
Yes	No	Hb_adm<1	Hb_exit<1:	47.45053	47.45053	7.963035	7.963035	No
Yes	Yes	Hb_adm<1	Hb_exit<1:	29.23622	29.23622	6.834936	6.834936	No
Yes	Yes	Hb_adm<1	Hb_exit<1:	2.598578	2.598578	9.525991	9.525991	Yes
No	Yes	Hb_adm>=	Hb_exit<1:	96.40399	96.40399	13.87903	13.87903	No
Yes	No	Hb_adm<1	Hb_exit<1:	10.26396	10.26396	5.322774	5.322774	Yes
Yes	No	Hb_adm<1	Hb_exit<1:	6.440939	6.440939	6.210708	6.210708	Yes
Yes	Yes	Hb_adm<1	Hb_exit<1:	31.8195	3.260259	12.63928	15.71599	No
Yes	Yes	Hb_adm<1	Hb_exit<1:	19.74222	19.74222	5.417159	5.417159	No
Yes	Yes	Hb_adm<1	Hb_exit<1:	37.44465	3.557697	22.81539	13.48042	No
Yes	No	Hb_adm<1	Hb_exit<1:	5.076495	5.076495	6.0831	6.0831	Yes
Yes	Yes	Hb_adm<1	Hb_exit<1:	47.76075	47.76075	6.419081	6.419081	No
Yes		Hb_adm<110		35.40409	35.40409	9.296657	9.296657	No
No	No	Hb_adm>=	Hb_exit<1:	40.38095	33.85693	6.512147	4.286148	No
No		Hb_adm>=110		55.49608	55.49608	3.487791	3.487791	No
No	No	Hb_adm>=	Hb_exit<1:	85.63715	85.63715	5.578312	5.578312	No
Yes	Yes	Hb_adm<1	Hb_exit<1:	4.30565	4.30565	4.161594	4.161594	Yes
Yes	Yes	Hb_adm<1	Hb_exit<1:	50.99384	50.99384	8.538251	8.538251	No
No	No	Hb_adm>=	Hb_exit<1:	7.72227	7.72227	13.63784	13.63784	Yes
Yes	Yes	Hb_adm<1	Hb_exit<1:	7.37391	5.991141	11.21658	12.14137	Yes
Yes		Hb_adm<110		5.474959	5.474959	8.031912	8.031912	Yes
Yes	No	Hb_adm<1	Hb_exit<1:	30.36023	46.34541	6.600292	0.671223	No
Yes	No	Hb_adm<1	Hb_exit<1:	1.175951	3.624185	20.67947	13.82293	Yes
Yes	No	Hb_adm<1	Hb_exit<1:	16.45395	16.45395	13.06965	13.06965	No
Yes	Yes	Hb_adm<1	Hb_exit<1:	32.11377	32.11377	6.459455	6.459455	No
Yes	No	Hb_adm<1	Hb_exit<1:	22.99525	22.99525	7.498701	7.498701	No
Yes	No	Hb_adm<1	Hb_exit<1:	6.586069	6.586069	8.704843	8.704843	Yes
Yes	No	Hb_adm<1	Hb_exit<1:	12.58781	12.58781	8.039776	8.039776	No
Yes	Yes	Hb_adm<1	Hb_exit<1:	94.53	94.53	8.1	8.1	No
Yes	Yes	Hb_adm<1	Hb_exit<1:	5.113863	11.24546	7.020412	6.662572	Yes
No	No	Hb_adm>=	Hb_exit<1:	20.85833	20.85833	7.022944	7.022944	No

Yes		Hb_adm<110		4.710939	4.710939	13.46258	13.46258	Yes
Yes	Yes	Hb_adm<1	Hb_exit<1:	18.41112	18.41112	9.683381	9.683381	No
Yes	Yes	Hb_adm<1	Hb_exit<1:	28.83847	28.83847	6.178175	6.178175	No
Yes	Yes	Hb_adm<1	Hb_exit<1:	11.18167	11.18167	16.42933	16.42933	Yes
Yes	No	Hb_adm<1	Hb_exit<1:	12.86011	12.86011	8.841555	8.841555	No
Yes	Yes	Hb_adm<1	Hb_exit<1:	6.098464	4.055957	3.008006	16.13499	Yes
Yes	No	Hb_adm<1	Hb_exit<1:	10.9808	10.9808	10.21046	10.21046	Yes
Yes	Yes	Hb_adm<1	Hb_exit<1:	12.64497	8.162857	6.782049	9.396494	No
Yes	Yes	Hb_adm<1	Hb_exit<1:	14.02513	14.02513	5.716422	5.716422	No
Yes	Yes	Hb_adm<1	Hb_exit<1:	54.82322	54.82322	18.28047	18.28047	No
Yes	Yes	Hb_adm<1	Hb_exit<1:	22.30696	22.30696	12.70537	12.70537	No
Yes	Yes	Hb_adm<1	Hb_exit<1:	5.91157	6.328538	9.723664	9.725682	Yes
Yes	Yes	Hb_adm<1	Hb_exit<1:	74.73368	74.73368	36.06147	36.06147	No
Yes	Yes	Hb_adm<1	Hb_exit<1:	1.702596	1.702596	28.02033	28.02033	Yes
No	No	Hb_adm>=	Hb_exit<1:	19.05095	19.05095	5.092589	5.092589	No
Yes		Hb_adm<110		31.60944	31.60944	9.98741	9.98741	No
Yes	Yes	Hb_adm<1	Hb_exit<1:	9.122002	24.77457	23.54711	13.85647	Yes
Yes	No	Hb_adm<1	Hb_exit<1:	28.60227	28.60227	6.161796	6.161796	No
Yes	No	Hb_adm<1	Hb_exit<1:	32.26208	32.26208	7.211872	7.211872	No
Yes	Yes	Hb_adm<1	Hb_exit<1:	11.69963	11.69963	8.98456	8.98456	Yes
Yes	No	Hb_adm<1	Hb_exit<1:	3.857793	3.857793	12.72705	12.72705	Yes
Yes	Yes	Hb_adm<1	Hb_exit<1:	11.90784	11.90784	9.105052	9.105052	Yes
Yes		Hb_adm<110		3.392814	3.392814	10.60914	10.60914	Yes
Yes	No	Hb_adm<1	Hb_exit<1:	18.95953	18.95953	8.022494	8.022494	No
No		Hb_adm>=110		39.60195	39.60195	7.644331	7.644331	No
Yes	Yes	Hb_adm<1	Hb_exit<1:	4.107825	4.107825	9.579388	9.579388	Yes
Yes		Hb_adm<110		1.668353	1.668353	12.77405	12.77405	Yes
Yes		Hb_adm<110		3.44	3.44	40	40	Yes
Yes	Yes	Hb_adm<1	Hb_exit<1:	8.101207	8.101207	16.84765	16.84765	Yes
Yes		Hb_adm<110		1.188881	1.188881	28.29528	28.29528	Yes
Yes		Hb_adm<110		4.143487	4.143487	6.941167	6.941167	Yes
Yes		Hb_adm<110		21.48672	21.48672	6.923322	6.923322	No
Yes	Yes	Hb_adm<1	Hb_exit<1:	7.509378	7.509378	16.48299	16.48299	Yes
Yes	Yes	Hb_adm<1	Hb_exit<1:	6.560283	6.560283	6.136386	6.136386	Yes
Yes	Yes	Hb_adm<1	Hb_exit<1:	1.32862	1.32862	39.91707	39.91707	Yes
Yes	No	Hb_adm<1	Hb_exit<1:	5.62615	5.62615	8.170511	8.170511	Yes
Yes	Yes	Hb_adm<1	Hb_exit<1:	5.531889	0.817594	12.6894	11.85578	Yes
Yes	Yes	Hb_adm<1	Hb_exit<1:	3.519642	3.519642	20.01603	20.01603	Yes
No		Hb_adm>=110		10.5556	10.5556	7.599537	7.599537	Yes
No	No	Hb_adm>=	Hb_exit<1:	3.056318	3.056318	3.960773	3.960773	Yes
Yes	Yes	Hb_adm<1	Hb_exit<1:	4.399109	4.399109	13.59006	13.59006	Yes
Yes		Hb_adm<110		2.1887	2.1887	16.8931	16.8931	Yes
Yes	No	Hb_adm<1	Hb_exit<1:	9.331387	9.331387	9.059351	9.059351	Yes
Yes		Hb_adm<110		4.295962	4.295962	33.02292	33.02292	Yes
Yes	Yes	Hb_adm<1	Hb_exit<1:	28.32574	81.64174	29.9943	31.69237	No
No	No	Hb_adm>=	Hb_exit<1:	27.2062	27.2062	6.612007	6.612007	No
Yes		Hb_adm<110		20.85756	6.078996	11.79765	9.704469	No
Yes	Yes	Hb_adm<1	Hb_exit<1:	7.288766	7.288766	8.056838	8.056838	Yes
Yes		Hb_adm<110		11.33847	11.33847	12.02864	12.02864	Yes
No		Hb_adm>=110		5.453103	5.453103	14.00529	14.00529	Yes
Yes	Yes	Hb_adm<1	Hb_exit<1:	2.534027	2.534027	10.56823	10.56823	Yes

Yes	Yes	Hb_adm<1	Hb_exit<1:	3.92	3.92	9.21	9.21	Yes
No	No	Hb_adm>=	Hb_exit<1:	3.978064	3.978064	7.835589	7.835589	Yes
Yes		Hb_adm<110		8.130001	8.130001	11.9415	11.9415	Yes
Yes		Hb_adm<110		8.754049		12.81321		Yes
Yes	Yes	Hb_adm<1	Hb_exit<1:	1.139833	1.139833	25.6701	25.6701	Yes
Yes		Hb_adm<110		6.731351		32.48993		Yes
No		Hb_adm>=110		5.50432	5.50432	7.913425	7.913425	Yes
Yes		Hb_adm<110						
Yes		Hb_adm<110		47.27824	47.27824	9.088794	9.088794	No
No		Hb_adm>=110		11.22597	11.22597	8.110158	8.110158	Yes
Yes		Hb_adm<110		6.619589	6.619589	2.672576	2.672576	Yes
No	No	Hb_adm>=	Hb_exit<1:	13.43221	13.43221	7.413728	7.413728	No
No	No	Hb_adm>=	Hb_exit<1:	28.76402	28.76402	6.097639	6.097639	No
Yes	No	Hb_adm<1	Hb_exit<1:	6.146217	6.146217	12.95628	12.95628	Yes
Yes		Hb_adm<110						
No	No	Hb_adm>=	Hb_exit<1:	13.34976	17.96	12.18745	7.16	No
Yes		Hb_adm<110		11.40033	11.40033	7.54344	7.54344	Yes
Yes		Hb_adm<110		11.09052	11.09052	4.759701	4.759701	Yes
No		Hb_adm>=110		6.253171	6.253171	9.84892	9.84892	Yes
No	No	Hb_adm>=	Hb_exit<1:	30.39333	30.39333	5.992856	5.992856	No
Yes		Hb_adm<110		11.43038	11.43038	13.18856	13.18856	Yes
Yes	No	Hb_adm<1	Hb_exit<1:	12.55	12.55	13.11	13.11	No
No	No	Hb_adm>=	Hb_exit<1:	15.37252	15.37252	8.406827	8.406827	No
No		Hb_adm>=110		43.59892	43.59892	5.259551	5.259551	No
Yes	Yes	Hb_adm<1	Hb_exit<1:	3.440762	3.440762	17.23298	17.23298	Yes
Yes	Yes	Hb_adm<1	Hb_exit<1:	13.70297	13.70297	9.28206	9.28206	No
Yes	Yes	Hb_adm<1	Hb_exit<1:	106.0967	106.0967	14.64091	14.64091	No
No	No	Hb_adm>=	Hb_exit<1:	7.991663	7.991663	6.467957	6.467957	Yes
Yes		Hb_adm<110		45.30626	45.30626	10.51584	10.51584	No
No	No	Hb_adm>=	Hb_exit<1:	31.75553	31.75553	4.930264	4.930264	No
Yes	Yes	Hb_adm<1	Hb_exit<1:	1.817455	1.817455	7.636588	7.636588	Yes
Yes	Yes	Hb_adm<1	Hb_exit<1:	15.30782	44.46997	12.62787	14.46602	No
Yes	Yes	Hb_adm<1	Hb_exit<1:	10.28423	10.28423	12.72677	12.72677	Yes
Yes	No	Hb_adm<1	Hb_exit<1:	38.45992	38.45992	10.8621	10.8621	No
No	No	Hb_adm>=	Hb_exit<1:	20.85737	22.93575	6.827931	4.793709	No
No	Yes	Hb_adm>=	Hb_exit<1:	4.623087	4.623087	14.85147	14.85147	Yes
Yes	Yes	Hb_adm<1	Hb_exit<1:	136.5287	136.5287	9.533844	9.533844	No
No	Yes	Hb_adm>=	Hb_exit<1:	45.79483	45.79483	5.869466	5.869466	No
Yes	Yes	Hb_adm<1	Hb_exit<1:	11.01866	11.01866	5.063785	5.063785	Yes
Yes	No	Hb_adm<1	Hb_exit<1:	50.89204	50.89204	1.970697	1.970697	No
Yes		Hb_adm<110		17.4535	17.4535	10.33647	10.33647	No
Yes	No	Hb_adm<1	Hb_exit<1:	6.752121	28.53586	28.50279	14.18274	Yes
Yes		Hb_adm<110		25.55734	25.55734	6.128924	6.128924	No
Yes	No	Hb_adm<1	Hb_exit<1:	4.005796	4.005796	9.875227	9.875227	Yes
Yes	Yes	Hb_adm<1	Hb_exit<1:	16.10465	16.10465	15.41261	15.41261	No
Yes		Hb_adm<110		7.260965		19.28065		Yes
Yes	Yes	Hb_adm<1	Hb_exit<1:	76.59867	76.59867	14.88825	14.88825	No
Yes	Yes	Hb_adm<1	Hb_exit<1:	16.08238	16.08238	12.21647	12.21647	No
Yes		Hb_adm<110		57.49943	57.49943	5.407555	5.407555	No
Yes	Yes	Hb_adm<1	Hb_exit<1:	16.13453	16.13453	21.02381	21.02381	No
Yes	Yes	Hb_adm<1	Hb_exit<1:	37.6323	37.6323	4.419157	4.419157	No

Yes	Yes	Hb_adm<1	Hb_exit<110						
No		Hb_adm>=110		25.34	25.34	5	5	No	
Yes	Yes	Hb_adm<1	Hb_exit<1:	39.37029	39.37029	7.497499	7.497499	No	
No		Hb_adm>=110		31.05774	31.05774	3.987114	3.987114	No	
Yes		Hb_adm<110		59.56452	59.56452	20.39219	20.39219	No	
Yes		Hb_adm<110		9.981011	9.981011	18.10867	18.10867	Yes	
Yes	Yes	Hb_adm<1	Hb_exit<1:	4.019977	4.019977	10.4783	10.4783	Yes	
Yes		Hb_adm<110		3.276144	3.276144	14.55723	14.55723	Yes	
Yes	Yes	Hb_adm<1	Hb_exit<1:	3.953929	3.953929	25.28351	25.28351	Yes	
No	No	Hb_adm>=	Hb_exit<1:	10.27361	10.27361	5.61306	5.61306	Yes	
Yes		Hb_adm<110		18.58294	18.58294	12.83406	12.83406	No	
Yes	Yes	Hb_adm<1	Hb_exit<1:	5.75917	5.75917	8.201047	8.201047	Yes	
Yes	No	Hb_adm<1	Hb_exit<1:	128.5828	128.5828	6.088064	6.088064	No	
No		Hb_adm>=110		3.305765	3.305765	12.18509	12.18509	Yes	
No		Hb_adm>=110		24.56507	24.56507	7.977197	7.977197	No	
No		Hb_adm>=110		26.47867	26.47867	6.01683	6.01683	No	
No	No	Hb_adm>=	Hb_exit<1:	111.7539	111.7539	7.760819	7.760819	No	
No	No	Hb_adm>=	Hb_exit<1:	33.64718	12.30827	5.237401	6.682097	No	
Yes	No	Hb_adm<1	Hb_exit<1:	38.56884	38.56884	6.892779	6.892779	No	
Yes	No	Hb_adm<1	Hb_exit<1:	30.22405	30.22405	7.0993	7.0993	No	
Yes		Hb_adm<110		86.03718	86.03718	8.452925	8.452925	No	
Yes	Yes	Hb_adm<1	Hb_exit<1:	76.1812	76.1812	7.572659	7.572659	No	
Yes	Yes	Hb_adm<1	Hb_exit<1:	46.46756	46.46756	11.21704	11.21704	No	
No	No	Hb_adm>=	Hb_exit<1:	10.60629	10.60629	8.140059	8.140059	Yes	
No	No	Hb_adm>=	Hb_exit<1:	10.92	10.92	8.1	8.1	Yes	
Yes	No	Hb_adm<1	Hb_exit<1:	23.69882	23.69882	7.060594	7.060594	No	
Yes	Yes	Hb_adm<1	Hb_exit<1:	17.35667	17.35667	7.888634	7.888634	No	
Yes	No	Hb_adm<1	Hb_exit<1:	6.013858	6.013858	18.06025	18.06025	Yes	
Yes	Yes	Hb_adm<1	Hb_exit<1:	5.527539	5.527539	19.60384	19.60384	Yes	
Yes	No	Hb_adm<1	Hb_exit<1:	2.890779	2.890779	5.581626	5.581626	Yes	
Yes		Hb_adm<110		31.64871	31.64871	5.115265	5.115265	No	
Yes	Yes	Hb_adm<1	Hb_exit<1:	16.1667	16.1667	4.565107	4.565107	No	
No	No	Hb_adm>=	Hb_exit<1:	38.99985	38.99985	6.784263	6.784263	No	
Yes		Hb_adm<110		12.26427	12.26427	11.10608	11.10608	No	
Yes		Hb_adm<110		6.069313	6.069313	7.078817	7.078817	Yes	
No	Yes	Hb_adm>=	Hb_exit<1:	38.47629	38.47629	4.262064	4.262064	No	
Yes	Yes	Hb_adm<1	Hb_exit<1:	8.836188	24.91599	9.325016	6.932406	Yes	

ID_exit	IDAAI_adm	IDA_exit	IDAAI_exit	sTfRhigh_a	sTfRhigh_e	SF_cat_exi	SF_cat_ad	B12_statu
No	No	No	No	No	No	SF_exit<12	SF_adm>=12	
No	No	No	No	Yes	Yes	SF_exit<12	SF_adm>=12	
No	No			Yes	Yes	SF_exit<12	SF_adm>=12	
No	No	No	No	Yes	Yes	SF_exit<12	SF_adm>=	moderate
Yes	Yes	No	Yes	Yes	Yes	SF_exit<12	SF_adm>=12	
							SF_adm>=12	
No	No	No	No	No	No	SF_exit<12	SF_adm>=12	
No	No	No	No	Yes	Yes	SF_exit<12	SF_adm>=12	
	No			No			SF_adm>=12	
Yes	Yes			Yes	Yes	SF_exit<12	SF_adm<12	
No	No	No	No	No	No	SF_exit<12	SF_adm>=12	
Yes	Yes			Yes	Yes	SF_exit<12	SF_adm<12	
Yes	Yes	No	No	No	Yes	SF_exit<12	SF_adm<1	moderate
No	No	No	No	Yes	No	SF_exit<12	SF_adm>=12	
No	No	No	No	No	No	SF_exit<12	SF_adm>=12	
Yes	Yes	Yes	Yes	Yes	Yes	SF_exit<12	SF_adm<12	
No	No			No	No	SF_exit<12	SF_adm>=12	
No	No	No	No	No	No	SF_exit<12	SF_adm>=12	
No	No			No	No	SF_exit<12	SF_adm>=12	
Yes	Yes	Yes	Yes	No	No	SF_exit<12	SF_adm<12	
No	No	No	No	Yes	Yes	SF_exit<12	SF_adm>=12	
No	No	No	No	No	No	SF_exit<12	SF_adm>=	marked de
Yes	Yes	No	No	No	No	SF_exit<12	SF_adm<1	moderate
							SF_adm>=12	
No	No			No	No	SF_exit<12	SF_adm>=12	
No	No	No	No	Yes	Yes	SF_exit<12	SF_adm>=	marked de
No	No	No	No	Yes	Yes	SF_exit<12	SF_adm>=	moderate
Yes	Yes	No	Yes	Yes	Yes	SF_exit<12	SF_adm>=	adequate
Yes	Yes	Yes	Yes	Yes	No	SF_exit<12	SF_adm<1	adequate
No	No	No	No	No	No	SF_exit<12	SF_adm>=	marked de
Yes	Yes	No	No	Yes	Yes	SF_exit<12	SF_adm<1	marked de
Yes	Yes			Yes	Yes	SF_exit<12	SF_adm>=12	
No	No	No	No	No	No	SF_exit<12	SF_adm>=	adequate
Yes	No	No	No	No	No	SF_exit<12	SF_adm<1	moderate
No	No	No	No	Yes	Yes	SF_exit<12	SF_adm>=	marked de
No	No	No	No	No	No	SF_exit<12	SF_adm>=	adequate
Yes	No	No	No	Yes	Yes	SF_exit<12	SF_adm<1	moderate
No	No	No	No	No	No	SF_exit<12	SF_adm>=	marked de
No	No	No	No	Yes	No	SF_exit<12	SF_adm>=	adequate
Yes	Yes	No	No	No	No	SF_exit<12	SF_adm<1	marked de
Yes	Yes	Yes	Yes	Yes	Yes	SF_exit<12	SF_adm<1	adequate
Yes	Yes	Yes	Yes	Yes	Yes	SF_exit<12	SF_adm<1	moderate
Yes	Yes			Yes	Yes	SF_exit<12	SF_adm>=12	
Yes	Yes			No	No	SF_exit<12	SF_adm<12	
No	No	No	No	No	No	SF_exit<12	SF_adm>=12	
No	No	No	No	No	Yes	SF_exit<12	SF_adm>=	moderate
No	No			No	No	SF_exit<12	SF_adm>=	moderate
No	No	No	No	No	Yes	SF_exit<12	SF_adm>=	moderate
No	No	No	No	No	No	SF_exit<12	SF_adm>=	moderate
No	No	No	No	No	No	SF_exit<12	SF_adm>=	moderate

No	No	No	No	No	No	SF_exit<12	SF_adm>=	marked de
Yes	Yes	No	Yes	Yes	Yes	SF_exit<12	SF_adm>=	marked de
Yes	Yes	No	Yes	Yes	Yes	SF_exit<12	SF_adm>=	moderate
No	No	No	No	No	No	SF_exit<12	SF_adm>=12	
Yes	Yes	No	No	No	No	SF_exit<12	SF_adm>=	marked de
No	Yes	No	No	Yes	No	SF_exit<12	SF_adm<1	moderate
No	No	No	No	Yes	Yes	SF_exit<12	SF_adm>=12	
No	No			No	No	SF_exit<12	SF_adm>=	adequate
Yes	Yes	No	Yes	Yes	Yes	SF_exit<12	SF_adm>=12	
							SF_adm>=12	
Yes	Yes	Yes	Yes	Yes	Yes	SF_exit<12	SF_adm<1	marked de
No	No	No	No	No	No	SF_exit<12	SF_adm>=	moderate
No	No	No	No	Yes	Yes	SF_exit<12	SF_adm>=12	
Yes	No	No	No	Yes	Yes	SF_exit<12	SF_adm>=	moderate
Yes	Yes			Yes	Yes	SF_exit<12	SF_adm<12	
Yes	Yes	No	No	Yes	Yes	SF_exit<12	SF_adm<12	
Yes	Yes	Yes	Yes	Yes	Yes	SF_exit<12	SF_adm<1	moderate
Yes	Yes			No	No	SF_exit<12	SF_adm>=12	
Yes	No	No	No	No	No	SF_exit<12	SF_adm>=	moderate
No	Yes	No	No	Yes	No	SF_exit<12	SF_adm>=	moderate
No	No	No	No	No	No	SF_exit<12	SF_adm>=	adequate
No	No	No	No	No	No	SF_exit<12	SF_adm>=	marked de
Yes	Yes	Yes	Yes	Yes	Yes	SF_exit<12	SF_adm<1	adequate
No	No	No	No	No	No	SF_exit<12	SF_adm>=	moderate
No	No	No	No	No	No	SF_exit<12	SF_adm>=	moderate
Yes	No			Yes	Yes	SF_exit<12	SF_adm<12	
No	No	No	No	Yes	Yes	SF_exit<12	SF_adm>=	moderate
No	No			Yes	Yes	SF_exit<12	SF_adm>=12	
Yes	Yes	No	Yes	Yes	Yes	SF_exit<12	SF_adm>=	moderate
No	No	No	No	No	No	SF_exit<12	SF_adm>=	adequate
	Yes			Yes			SF_adm<12	
							SF_adm>=12	
Yes	Yes	Yes	Yes	Yes	Yes	SF_exit<12	SF_adm<12	
							SF_adm>=12	
No	No			Yes	Yes	SF_exit<12	SF_adm>=12	
Yes	Yes			Yes	Yes	SF_exit<12	SF_adm<12	
Yes	No			No	No	SF_exit<12	SF_adm>=12	
No	No			No	No	SF_exit<12	SF_adm>=12	
No	No	No	No	Yes	Yes	SF_exit<12	SF_adm>=12	
No	No			No	No	SF_exit<12	SF_adm>=12	
Yes	Yes			Yes	Yes	SF_exit<12	SF_adm<12	
Yes	Yes	Yes	Yes	Yes	Yes	SF_exit<12	SF_adm<1	marked de
Yes	No	No	No	No	Yes	SF_exit<12	SF_adm>=	marked de
	Yes			Yes			SF_adm>=12	
Yes	Yes	No	Yes	No	Yes	SF_exit<12	SF_adm>=	moderate
No	No	No	No	No	No	SF_exit<12	SF_adm>=12	
Yes	Yes			Yes	Yes	SF_exit<12	SF_adm<12	
Yes	Yes	No	No	Yes	Yes	SF_exit<12	SF_adm<1	marked de
Yes	Yes			Yes	Yes	SF_exit<12	SF_adm>=12	
No	No			No	No	SF_exit<12	SF_adm>=12	
No	No	No	No	No	No	SF_exit<12	SF_adm>=	moderate

							SF_adm>=12	
Yes	Yes	No	No	No	No	SF_exit<12	SF_adm>=	moderate
Yes	Yes	Yes	Yes	Yes	Yes	SF_exit<12	SF_adm<1	moderate
							SF_adm>=12	
No	No	No	No	Yes	Yes	SF_exit<12	SF_adm>=	marked de
No	No			Yes	Yes	SF_exit<12	SF_adm>=12	
Yes	Yes	Yes	Yes	Yes	Yes	SF_exit<12	SF_adm<1	moderate
Yes	No	No	No	No	No	SF_exit<12	SF_adm>=	moderate
Yes	Yes	No	No	No	No	SF_exit<12	SF_adm<1	moderate
Yes	No			Yes	Yes	SF_exit<12	SF_adm>=	moderate
No	No	No	No	No	No	SF_exit<12	SF_adm>=12	
Yes	Yes	No	Yes	No	No	SF_exit<12	SF_adm>=	moderate
							SF_adm>=12	
No	No			Yes	Yes	SF_exit<12	SF_adm>=	moderate
Yes	Yes			Yes	Yes	SF_exit<12	SF_adm<1	moderate
Yes	Yes			No	No	SF_exit<12	SF_adm<12	
Yes	No	No	Yes	No	No	SF_exit<12	SF_adm>=12	
No	No	No	No	No	No	SF_exit<12	SF_adm>=	adequate
No	No			No	No	SF_exit<12	SF_adm>=	moderate
No	No			No	No	SF_exit<12	SF_adm>=12	
No	No	No	No	No	No	SF_exit<12	SF_adm>=	marked de
Yes	Yes	No	No	Yes	Yes	SF_exit<12	SF_adm<1	moderate
No	No			No	No	SF_exit<12	SF_adm>=	moderate
							SF_adm>=12	
No	No	No	No	No	No	SF_exit<12	SF_adm>=12	
No	No	No	No	Yes	Yes	SF_exit<12	SF_adm>=12	
Yes	Yes			Yes	Yes	SF_exit<12	SF_adm<1	adequate
No	No			No	No	SF_exit<12	SF_adm>=12	
No	No	No	No	No	No	SF_exit<12	SF_adm>=	marked de
No	No	No	No	No	No	SF_exit<12	SF_adm>=	moderate
No	No	No	No	No	No	SF_exit<12	SF_adm>=12	
Yes	Yes	No	Yes	Yes	Yes	SF_exit<12	SF_adm>=12	
No	No	No	No	No	No	SF_exit<12	SF_adm>=	adequate
Yes	Yes	No	No	Yes	Yes	SF_exit<12	SF_adm<12	
Yes	Yes	No	Yes	No	No	SF_exit<12	SF_adm>=	severe def
No	No	No	No	Yes	Yes	SF_exit<12	SF_adm>=	moderate
No	No	No	No	No	No	SF_exit<12	SF_adm>=	moderate
No	No			No	No	SF_exit<12	SF_adm>=12	
Yes	No	No	No	No	No	SF_exit<12	SF_adm<12	
Yes	Yes			Yes	Yes	SF_exit<12	SF_adm>=12	
No	No	No	No	No	No	SF_exit<12	SF_adm>=	marked de
No	No	No	No	Yes	Yes	SF_exit<12	SF_adm>=	marked de
No	No	No	No	No	No	SF_exit<12	SF_adm>=	adequate
Yes	Yes			Yes	Yes	SF_exit<12	SF_adm<12	
No	No			No	No	SF_exit<12	SF_adm>=12	
No	No	No	No	Yes	Yes	SF_exit<12	SF_adm>=	marked de
No	No	No	No	Yes	Yes	SF_exit<12	SF_adm>=	adequate
No	No	No	No	No	No	SF_exit<12	SF_adm>=12	
Yes	Yes	Yes	Yes	Yes	Yes	SF_exit<12	SF_adm<12	
No	No			No	No	SF_exit<12	SF_adm>=12	
							SF_adm>=12	

No	No	No	No	Yes	Yes	SF_exit<12	SF_adm>=12	
							SF_adm>=12	
							SF_adm>=12	
No	No			Yes	Yes	SF_exit<12	SF_adm>=12	
							SF_adm>=12	
							SF_adm>=12	
Yes	Yes	No	Yes	Yes	Yes	SF_exit<12	SF_adm>=12	
Yes	Yes	Yes	Yes	No	No	SF_exit<12	SF_adm<12	
No	No	No	No	No	No	SF_exit<12	SF_adm>=12	
							SF_adm>=12	
No	No	No	No	Yes	Yes	SF_exit<12	SF_adm>=12	
Yes	Yes			Yes	Yes	SF_exit<12	SF_adm<12	
No	No			No	No	SF_exit<12	SF_adm>=12	
Yes	Yes			Yes	Yes	SF_exit<12	SF_adm<12	
Yes	Yes			Yes	Yes	SF_exit<12	SF_adm>=12	
Yes	Yes			Yes	Yes	SF_exit<12	SF_adm<12	
Yes	Yes	No	No	No	No	SF_exit<12	SF_adm<12	
No	No	No	No	No	No	SF_exit<12	SF_adm>=12	
No	No	No	No	Yes	Yes	SF_exit<12	SF_adm>=12	
No	No	No	No	No	No	SF_exit<12	SF_adm>=12	
No	No	No	No	No	No	SF_exit<12	SF_adm>=12	
No	No	No	No	No	No	SF_exit<12	SF_adm>=12	
Yes	Yes	No	No	No	No	SF_exit<12	SF_adm>=12	
No	No	No	No	Yes	Yes	SF_exit<12	SF_adm>=12	
No	No	No	No	Yes	Yes	SF_exit<12	SF_adm>=12	
Yes	Yes	Yes	Yes	Yes	Yes	SF_exit<12	SF_adm<1	marked de
No	No	No	No	No	No	SF_exit<12	SF_adm>=12	
Yes	Yes	Yes	Yes	Yes	Yes	SF_exit<12	SF_adm<12	
No	No			No	No	SF_exit<12	SF_adm>=12	
							SF_adm>=12	
Yes	Yes	No	No	No	No	SF_exit<12	SF_adm>=12	
Yes	Yes			Yes	Yes	SF_exit<12	SF_adm>=12	
No	No	No	No	No	No	SF_exit<12	SF_adm>=	marked de
							SF_adm>=12	
No	No			Yes	Yes	SF_exit<12	SF_adm>=12	
Yes	Yes	No	Yes	Yes	Yes	SF_exit<12	SF_adm>=	marked de
No	No			Yes	Yes	SF_exit<12	SF_adm>=12	
No	No	No	No	Yes	Yes	SF_exit<12	SF_adm>=12	
No	No	No	No	Yes	Yes	SF_exit<12	SF_adm>=	moderate
No	No	No	No	Yes	Yes	SF_exit<12	SF_adm>=	moderate
No	No			Yes	Yes	SF_exit<12	SF_adm>=12	
No	No	No	No	Yes	Yes	SF_exit<12	SF_adm>=	marked de
No	No	No	No	Yes	Yes	SF_exit<12	SF_adm>=	marked de
No	No			No	No	SF_exit<12	SF_adm>=12	
Yes	Yes	No	Yes	Yes	Yes	SF_exit<12	SF_adm>=	moderate
Yes	Yes			Yes	Yes	SF_exit<12	SF_adm<12	
No	No	No	No	Yes	Yes	SF_exit<12	SF_adm>=	moderate
No	No			Yes	Yes	SF_exit<12	SF_adm>=12	
No	No			Yes	Yes	SF_exit<12	SF_adm>=12	
No	No	No	No	Yes	Yes	SF_exit<12	SF_adm>=	moderate
No	No			No	No	SF_exit<12	SF_adm>=12	

Yes	Yes			Yes	Yes	SF_exit<12	SF_adm<1	moderate
No	No	No	No	Yes	Yes	SF_exit<12	SF_adm>=	moderate
No	No			No	No	SF_exit<12	SF_adm>=12	
No	No			Yes	Yes	SF_exit<12	SF_adm>=	moderate
Yes	No			No	Yes	SF_exit<12	SF_adm>=	marked de
Yes	Yes			No	No	SF_exit<12	SF_adm>=	moderate
No	No	No	No	No	No	SF_exit<12	SF_adm>=	severe def
							SF_adm>=12	
Yes	Yes	No	Yes	No	No	SF_exit<12	SF_adm>=12	
No	No	No	No	No	No	SF_exit<12	SF_adm>=	moderate
No	No	No	No	Yes	Yes	SF_exit<12	SF_adm>=12	
							SF_adm>=12	
No	No	No	No	No	No	SF_exit<12	SF_adm>=12	
							SF_adm>=12	
No	No	No	No	Yes	Yes	SF_exit<12	SF_adm>=12	
Yes	Yes	Yes	Yes	No	No	SF_exit<12	SF_adm<12	
No	No	No	No	Yes	No	SF_exit<12	SF_adm>=	moderate
No	No	No	No	No	No	SF_exit<12	SF_adm>=12	
							SF_adm>=12	
Yes	Yes	No	No	Yes	Yes	SF_exit<12	SF_adm<12	
No	No			No	No	SF_exit<12	SF_adm>=12	
							SF_adm>=12	
Yes	Yes	Yes	Yes	Yes	Yes	SF_exit<12	SF_adm<12	
							SF_adm>=12	
No	No	No	No	Yes	Yes	SF_exit<12	SF_adm>=12	
Yes	Yes			Yes	Yes	SF_exit<12	SF_adm>=12	
No	No	No	No	No	No	SF_exit<12	SF_adm>=12	
No	No			No	No	SF_exit<12	SF_adm>=12	
No	No	No	No	No	No	SF_exit<12	SF_adm>=12	
No	Yes	No	No	Yes	No	SF_exit<12	SF_adm<12	
No	No			Yes	Yes	SF_exit<12	SF_adm>=12	
No	No	No	No	No	No	SF_exit<12	SF_adm>=12	
Yes	Yes	No	No	No	No	SF_exit<12	SF_adm>=12	
No	No	No	No	No	No	SF_exit<12	SF_adm>=12	
No	No	No	No	Yes	Yes	SF_exit<12	SF_adm>=12	
							SF_adm>=12	
Yes	Yes	No	No	Yes	Yes	SF_exit<12	SF_adm<12	
No	No	No	No	No	No	SF_exit<12	SF_adm>=12	
No	No	No	No	No	No	SF_exit<12	SF_adm>=12	
Yes	No			Yes	Yes	SF_exit<12	SF_adm>=12	
No	No	No	No	No	No	SF_exit<12	SF_adm>=12	
Yes	Yes	No	Yes	No	No	SF_exit<12	SF_adm>=12	
Yes	Yes	No	No	Yes	Yes	SF_exit<12	SF_adm<12	
No	No			No	No	SF_exit<12	SF_adm>=12	
No	No			No	No	SF_exit<12	SF_adm>=12	
	Yes			Yes			SF_adm<12	
No	No	No	No	No	No	SF_exit<12	SF_adm>=	moderate
Yes	No	No	No	Yes	Yes	SF_exit<12	SF_adm<12	
No	No	No	No	Yes	Yes	SF_exit<12	SF_adm>=	severe def
Yes	Yes	Yes	Yes	Yes	Yes	SF_exit<12	SF_adm<12	
No	No	No	No	No	No	SF_exit<12	SF_adm>=12	

							SF_adm>=12	
No	No	No	No	No	No	SF_exit<12	SF_adm>=	moderate
No	No			No	No	SF_exit<12	SF_adm>=12	
Yes	Yes	Yes	Yes	Yes	Yes	SF_exit<12	SF_adm<1	marked de
Yes	Yes			Yes	Yes	SF_exit<12	SF_adm>=12	
Yes	No			No	No	SF_exit<12	SF_adm>=12	
Yes	Yes	Yes	Yes	No	No	SF_exit<12	SF_adm<12	
No	No	No	No	Yes	No	SF_exit<12	SF_adm>=	moderate
Yes	Yes			Yes	Yes	SF_exit<12	SF_adm<1	adequate
Yes	Yes	No	Yes	Yes	Yes	SF_exit<12	SF_adm>=12	
Yes	Yes	Yes	Yes	No	No	SF_exit<12	SF_adm<1	marked de
Yes	Yes	No	No	Yes	Yes	SF_exit<12	SF_adm<1	adequate
Yes	Yes	Yes	Yes	Yes	Yes	SF_exit<12	SF_adm<1	marked de
No	No	No	No	No	No	SF_exit<12	SF_adm>=	moderate
No	No	No	No	No	No	SF_exit<12	SF_adm>=	moderate
Yes	Yes	No	Yes	No	No	SF_exit<12	SF_adm>=12	
No	No	No	No	No	No	SF_exit<12	SF_adm>=	marked de
No	No			Yes	Yes	SF_exit<12	SF_adm>=	moderate
Yes	Yes	Yes	Yes	No	No	SF_exit<12	SF_adm<1	moderate
No	No	No	No	Yes	Yes	SF_exit<12	SF_adm>=	moderate
No	No	No	No	No	No	SF_exit<12	SF_adm>=	moderate
No	No	No	No	No	No	SF_exit<12	SF_adm>=12	
No	No	No	No	No	No	SF_exit<12	SF_adm>=	moderate
Yes	Yes	No	No	Yes	Yes	SF_exit<12	SF_adm<1	marked de
Yes	Yes			Yes	Yes	SF_exit<12	SF_adm>=	moderate
No	No	No	No	No	No	SF_exit<12	SF_adm>=	moderate
No	No	No	No	No	No	SF_exit<12	SF_adm>=	marked de
No	No	No	No	Yes	Yes	SF_exit<12	SF_adm>=12	
No	No	No	No	Yes	Yes	SF_exit<12	SF_adm>=12	
No	No			No	No	SF_exit<12	SF_adm>=	moderate
Yes	Yes	No	No	No	No	SF_exit<12	SF_adm>=	moderate
No	No	No	No	Yes	Yes	SF_exit<12	SF_adm>=	marked de
No	No	No	No	Yes	Yes	SF_exit<12	SF_adm>=	marked de
No	No			Yes	Yes	SF_exit<12	SF_adm>=	moderate
No	No	No	No	Yes	Yes	SF_exit<12	SF_adm>=	moderate
No	No	No	No	Yes	Yes	SF_exit<12	SF_adm>=	marked de
No	No	No	No	Yes	Yes	SF_exit<12	SF_adm>=	moderate
No	No	No	No	No	No	SF_exit<12	SF_adm>=	moderate
							SF_adm>=12	
No	No	No	No	No	No	SF_exit<12	SF_adm>=	adequate
No	No	No	No	No	Yes	SF_exit<12	SF_adm>=	adequate
Yes	Yes	No	Yes	Yes	Yes	SF_exit<12	SF_adm>=	moderate
Yes	Yes	No	No	Yes	Yes	SF_exit<12	SF_adm<1	moderate
No	No			Yes	Yes	SF_exit<12	SF_adm>=12	
No	No	No	No	Yes	Yes	SF_exit<12	SF_adm>=	marked de
No	No	No	No	Yes	Yes	SF_exit<12	SF_adm>=	moderate
Yes	Yes	No	No	Yes	Yes	SF_exit<12	SF_adm<1	moderate
No	No	No	No	No	No	SF_exit<12	SF_adm>=	moderate
No	No	No	No	Yes	Yes	SF_exit<12	SF_adm>=12	
Yes	Yes	No	Yes	Yes	Yes	SF_exit<12	SF_adm>=	moderate
Yes	Yes	No	Yes	Yes	Yes	SF_exit<12	SF_adm>=	moderate

Yes	Yes	Yes	Yes	Yes	Yes	SF_exit<12	SF_adm<1	marked de
							SF_adm>=12	
Yes	Yes	Yes	Yes	Yes	Yes	SF_exit<12	SF_adm<12	
No	No			No	No	SF_exit<12	SF_adm>=	moderate
Yes	No	No	No	No	No	SF_exit<12	SF_adm>=	marked de
Yes	Yes			Yes	Yes	SF_exit<12	SF_adm>=12	
Yes	Yes	No	No	No	No	SF_exit<12	SF_adm>=	marked de
No	No	No	No	No	No	SF_exit<12	SF_adm>=	moderate
Yes	Yes	No	Yes	Yes	Yes	SF_exit<12	SF_adm>=	severe def
No	No	No	No	No	No	SF_exit<12	SF_adm>=	moderate
No	No	No	No	No	No	SF_exit<12	SF_adm>=	moderate
No	No	No	No	Yes	Yes	SF_exit<12	SF_adm>=	moderate
Yes	Yes			Yes	Yes	SF_exit<12	SF_adm<1	moderate
No	No			Yes	Yes	SF_exit<12	SF_adm>=	moderate
Yes	Yes	No	No	No	No	SF_exit<12	SF_adm>=	moderate
Yes	Yes	No	Yes	Yes	Yes	SF_exit<12	SF_adm>=	marked de
Yes	Yes	No	Yes	Yes	Yes	SF_exit<12	SF_adm>=12	
Yes	Yes	No	No	Yes	Yes	SF_exit<12	SF_adm<12	
Yes	Yes	No	No	Yes	Yes	SF_exit<12	SF_adm<1	severe def
	Yes			Yes			SF_adm<12	
							SF_adm>=12	
Yes	Yes	No	Yes	No	No	SF_exit<12	SF_adm>=	adequate
No	No	No	No	No	No	SF_exit<12	SF_adm>=	moderate
No	No			Yes	Yes	SF_exit<12	SF_adm>=12	
No	No	No	No	No	No	SF_exit<12	SF_adm>=	moderate
Yes	Yes	No	Yes	Yes	Yes	SF_exit<12	SF_adm>=	marked de
Yes	Yes	No	Yes	No	No	SF_exit<12	SF_adm>=12	
No	No	No	No	No	No	SF_exit<12	SF_adm>=12	
No	No	No	No	No	No	SF_exit<12	SF_adm>=12	
No	No			No	No	SF_exit<12	SF_adm>=12	
Yes	Yes	No	No	Yes	Yes	SF_exit<12	SF_adm<12	
No	No			No	No	SF_exit<12	SF_adm>=12	
No	No	No	No	Yes	Yes	SF_exit<12	SF_adm>=12	
Yes	Yes	No	Yes	Yes	Yes	SF_exit<12	SF_adm>=12	
Yes	Yes	Yes	Yes	Yes	Yes	SF_exit<12	SF_adm<12	
No	No	No	No	No	No	SF_exit<12	SF_adm>=12	
No	No	No	No	Yes	Yes	SF_exit<12	SF_adm>=12	
No	No	No	No	Yes	Yes	SF_exit<12	SF_adm>=12	
No	No	No	No	No	No	SF_exit<12	SF_adm>=12	
Yes	No			No	No	SF_exit<12	SF_adm>=12	
Yes	Yes			Yes	Yes	SF_exit<12	SF_adm>=12	
Yes	No	Yes	Yes	Yes	Yes	SF_exit<12	SF_adm<1	marked de
Yes	Yes	Yes	Yes	Yes	Yes	SF_exit<12	SF_adm<1	moderate
No	No			No	No	SF_exit<12	SF_adm>=12	
No	No	No	No	No	No	SF_exit<12	SF_adm>=	severe def
No	No			No	No	SF_exit<12	SF_adm>=12	
No	No	No	No	Yes	Yes	SF_exit<12	SF_adm>=	adequate
Yes	Yes	No	Yes	Yes	Yes	SF_exit<12	SF_adm>=12	
Yes	Yes	Yes	Yes	Yes	Yes	SF_exit<12	SF_adm<1	marked de
Yes	Yes	No	No	Yes	Yes	SF_exit<12	SF_adm<1	moderate
Yes	Yes	Yes	Yes	Yes	Yes	SF_exit<12	SF_adm<1	adequate

Yes	Yes			Yes	Yes	SF_exit<12	SF_adm>=12	
No	No	No	No	Yes	Yes	SF_exit<12	SF_adm>=	moderate
No	No	No	No	Yes	Yes	SF_exit<12	SF_adm>=12	
No	Yes	No	No	No	No	SF_exit<12	SF_adm>=	moderate
							SF_adm>=12	
Yes	Yes	Yes	Yes	Yes	Yes	SF_exit<12	SF_adm<1	moderate
No	No			Yes	Yes	SF_exit<12	SF_adm>=12	
No	No			Yes	Yes	SF_exit<12	SF_adm>=12	
Yes	Yes	Yes	Yes	Yes	Yes	SF_exit<12	SF_adm<1	severe def
No	No			Yes	Yes	SF_exit<12	SF_adm>=	severe def
No	No	No	No	Yes	Yes	SF_exit<12	SF_adm>=	moderate
No	No	No	No	No	No	SF_exit<12	SF_adm>=	moderate
No	No	No	No	Yes	Yes	SF_exit<12	SF_adm>=	marked de
No	No	No	No	No	No	SF_exit<12	SF_adm>=	marked de
Yes	Yes	No	No	No	No	SF_exit<12	SF_adm>=	moderate
Yes	No			No	No	SF_exit<12	SF_adm<12	
Yes	Yes	No	Yes	Yes	Yes	SF_exit<12	SF_adm>=12	
Yes	Yes	Yes	Yes	Yes	Yes	SF_exit<12	SF_adm<1	moderate
No	No	No	No	No	No	SF_exit<12	SF_adm>=	moderate
Yes	Yes	No	No	Yes	Yes	SF_exit<12	SF_adm<1	moderate
No	No	No	No	Yes	Yes	SF_exit<12	SF_adm>=12	
No	No			Yes	Yes	SF_exit<12	SF_adm>=12	
Yes	Yes	No	No	No	No	SF_exit<12	SF_adm<12	
No	No	No	No	No	No	SF_exit<12	SF_adm>=12	
Yes	Yes	No	No	Yes	Yes	SF_exit<12	SF_adm<12	
Yes	No	No	Yes	No	No	SF_exit<12	SF_adm>=	moderate
Yes	Yes	Yes	Yes	Yes	Yes	SF_exit<12	SF_adm<1	marked de
No	No	No	No	Yes	Yes	SF_exit<12	SF_adm>=12	
Yes	Yes	Yes	Yes	Yes	Yes	SF_exit<12	SF_adm<1	moderate
No	No	No	No	No	No	SF_exit<12	SF_adm>=12	
Yes	Yes	No	Yes	Yes	Yes	SF_exit<12	SF_adm>=	adequate
No	No			No	No	SF_exit<12	SF_adm>=	marked de
No	No			Yes	Yes	SF_exit<12	SF_adm>=12	
No	No	No	No	No	No	SF_exit<12	SF_adm>=12	
No	No	No	No	Yes	Yes	SF_exit<12	SF_adm>=12	
No	No	No	No	No	No	SF_exit<12	SF_adm>=12	
							SF_adm>=12	
No	No	No	No	Yes	Yes	SF_exit<12	SF_adm>=12	
No	No	No	No	No	No	SF_exit<12	SF_adm>=12	
No	No	No	No	No	No	SF_exit<12	SF_adm>=12	
No	No	No	No	Yes	Yes	SF_exit<12	SF_adm>=12	
No	No			No	No	SF_exit<12	SF_adm>=12	
							SF_adm>=12	
No	No			Yes	Yes	SF_exit<12	SF_adm>=12	
No	No			No	No	SF_exit<12	SF_adm>=12	
Yes	Yes	Yes	Yes	Yes	Yes	SF_exit<12	SF_adm<12	
No	No			No	No	SF_exit<12	SF_adm>=12	
No	No	No	No	No	No	SF_exit<12	SF_adm>=12	
No	No	No	No	Yes	Yes	SF_exit<12	SF_adm>=	moderate
Yes	Yes			Yes	Yes	SF_exit<12	SF_adm>=12	
							SF_adm>=12	

No	No	No	No	No	No	SF_exit<12	SF_adm>=12	
No	No	No	No	No	No	SF_exit<12	SF_adm>=	moderate
No	No	No	No	No	No	SF_exit<12	SF_adm>=	moderate
Yes	Yes	Yes	Yes	Yes	Yes	SF_exit<12	SF_adm<1	moderate
No	No	No	No	No	No	SF_exit<12	SF_adm>=12	
Yes	No	No	No	No	No	SF_exit<12	SF_adm<12	
No	No	No	No	No	No	SF_exit<12	SF_adm>=12	
Yes	No	No	No	Yes	Yes	SF_exit<12	SF_adm>=12	
No	No	No	No	No	No	SF_exit<12	SF_adm>=	marked de
No	No	No	No	Yes	Yes	SF_exit<12	SF_adm>=	marked de
Yes	No	No	Yes	Yes	Yes	SF_exit<12	SF_adm>=	moderate
No	No	No	No	No	No	SF_exit<12	SF_adm>=	moderate
No	No	No	No	Yes	Yes	SF_exit<12	SF_adm>=	marked de
	No			Yes			SF_adm>=12	
No	No	No	No	Yes	Yes	SF_exit<12	SF_adm>=	moderate
No	No			Yes	Yes	SF_exit<12	SF_adm>=	moderate
No	No	No	No	Yes	Yes	SF_exit<12	SF_adm>=	moderate
No	No			No	No	SF_exit<12	SF_adm>=12	
Yes	Yes	No	No	No	No	SF_exit<12	SF_adm<1	adequate
No	No			Yes	Yes	SF_exit<12	SF_adm>=12	
Yes	No			No	No	SF_exit<12	SF_adm>=	moderate
Yes	Yes	No	No	Yes	Yes	SF_exit<12	SF_adm<1	severe def
No	No	No	No	No	No	SF_exit<12	SF_adm>=	marked de
No	No	No	No	No	No	SF_exit<12	SF_adm>=	moderate
No	No	No	No	No	No	SF_exit<12	SF_adm>=	moderate
No	No			No	No	SF_exit<12	SF_adm>=12	
No	No	No	No	No	No	SF_exit<12	SF_adm>=	moderate
Yes	Yes	No	No	No	No	SF_exit<12	SF_adm<12	
No	No	No	No	Yes	Yes	SF_exit<12	SF_adm>=12	
Yes	No	No	Yes	Yes	Yes	SF_exit<12	SF_adm>=12	
Yes	Yes	No	No	Yes	Yes	SF_exit<12	SF_adm<12	
Yes	Yes	No	No	No	No	SF_exit<12	SF_adm<1	adequate
Yes	Yes	No	No	No	No	SF_exit<12	SF_adm<1	severe def
No	No	No	No	No	No	SF_exit<12	SF_adm>=	marked de
No	No	No	No	No	No	SF_exit<12	SF_adm>=12	
No	No	No	No	No	No	SF_exit<12	SF_adm>=	moderate
Yes	Yes			Yes	Yes	SF_exit<12	SF_adm<12	
							SF_adm>=12	
No	No			No	No	SF_exit<12	SF_adm>=12	
No	No	No	No	No	No	SF_exit<12	SF_adm>=12	
No	No	No	No	No	No	SF_exit<12	SF_adm>=	marked de
No	No	No	No	No	No	SF_exit<12	SF_adm>=12	
No	No			No	No	SF_exit<12	SF_adm>=12	
No	No	No	No	No	No	SF_exit<12	SF_adm>=12	
Yes	Yes	No	No	Yes	Yes	SF_exit<12	SF_adm>=	moderate
Yes	Yes			Yes	Yes	SF_exit<12	SF_adm<12	
No	No			No	No	SF_exit<12	SF_adm>=12	
							SF_adm>=12	
Yes	Yes			No	No	SF_exit<12	SF_adm<12	
No	No	No	No	No	No	SF_exit<12	SF_adm>=12	
Yes	No			No	No	SF_exit<12	SF_adm<12	

No	No	No	No	No	No	SF_exit<12	SF_adm>=12	
Yes	Yes			No	No	SF_exit<12	SF_adm>=12	
Yes	No			No	No	SF_exit<12	SF_adm>=12	
Yes	Yes			Yes	Yes	SF_exit<12	SF_adm<12	
No	No	No	No	No	No	SF_exit<12	SF_adm>=	adequate
							SF_adm>=12	
No	No	No	No	No	No	SF_exit<12	SF_adm>=12	
Yes	No			No	No	SF_exit<12	SF_adm>=12	
Yes	Yes	Yes	Yes	No	No	SF_exit<12	SF_adm<1	moderate
Yes	Yes	Yes	Yes	Yes	Yes	SF_exit<12	SF_adm<1	moderate
No	No	No	No	Yes	Yes	SF_exit<12	SF_adm>=	moderate
Yes	Yes			Yes	Yes	SF_exit<12	SF_adm>=12	
No	No			No	No	SF_exit<12	SF_adm>=12	
Yes	Yes	No	No	No	No	SF_exit<12	SF_adm>=	marked de
No	No	No	No	No	No	SF_exit<12	SF_adm>=12	
No	No	No	No	No	No	SF_exit<12	SF_adm>=12	
No	No			Yes	Yes	SF_exit<12	SF_adm>=12	
No	No	No	No	No	No	SF_exit<12	SF_adm>=	marked de
Yes	Yes	No	No	Yes	Yes	SF_exit<12	SF_adm>=	severe def
No	No	No	No	Yes	Yes	SF_exit<12	SF_adm>=	moderate
Yes	No	No	No	Yes	Yes	SF_exit<12	SF_adm<12	
No	No			Yes	Yes	SF_exit<12	SF_adm>=12	
Yes	No			Yes	Yes	SF_exit<12	SF_adm<1	moderate
No	No	No	No	No	No	SF_exit<12	SF_adm>=	moderate
Yes	Yes			No	No	SF_exit<12	SF_adm>=12	
No	No			No	No	SF_exit<12	SF_adm>=12	
Yes	No	No	No	Yes	Yes	SF_exit<12	SF_adm>=12	
Yes	Yes	Yes	Yes	No	No	SF_exit<12	SF_adm<1	severe def
No	No	No	No	Yes	Yes	SF_exit<12	SF_adm>=	moderate
No	No	No	No	Yes	Yes	SF_exit<12	SF_adm>=	adequate
Yes	No	No	No	Yes	Yes	SF_exit<12	SF_adm>=	adequate
Yes	No			No	No	SF_exit<12	SF_adm>=	adequate
No	No	No	No	No	No	SF_exit<12	SF_adm>=12	
	No			Yes			SF_adm>=12	
Yes	Yes			Yes	Yes	SF_exit<12	SF_adm<12	
No	No			Yes	Yes	SF_exit<12	SF_adm>=12	
No	No	No	No	No	No	SF_exit<12	SF_adm>=	moderate
No	No	No	No	No	No	SF_exit<12	SF_adm>=	moderate
No	No			Yes	Yes	SF_exit<12	SF_adm>=12	
No	No			No	No	SF_exit<12	SF_adm>=	adequate
No	No			No	No	SF_exit<12	SF_adm>=12	
No	Yes	No	No	Yes	No	SF_exit<12	SF_adm>=	moderate
No	No			No	No	SF_exit<12	SF_adm>=12	
Yes	No			No	No	SF_exit<12	SF_adm>=12	
Yes	No			No	No	SF_exit<12	SF_adm<12	
Yes	Yes			No	No	SF_exit<12	SF_adm<12	
Yes	No	No	No	Yes	Yes	SF_exit<12	SF_adm<1	moderate
No	No	No	No	No	No	SF_exit<12	SF_adm>=	moderate
Yes	Yes	No	Yes	No	No	SF_exit<12	SF_adm>=	moderate
Yes	Yes			No	No	SF_exit<12	SF_adm>=	moderate
No	No			No	No	SF_exit<12	SF_adm>=	moderate

Yes	Yes	No	Yes	No	No	SF_exit<12	SF_adm>=	moderate
Yes	Yes			Yes	Yes	SF_exit<12	SF_adm>=12	
No	No			No	No	SF_exit<12	SF_adm>=12	
							SF_adm>=12	
Yes	No	No	No	Yes	Yes	SF_exit<12	SF_adm<12	
No	No			No	No	SF_exit<12	SF_adm>=12	
Yes	Yes	No	No	Yes	Yes	SF_exit<12	SF_adm<12	
Yes	Yes			No	No	SF_exit<12	SF_adm>=12	
							SF_adm>=12	
Yes	Yes			No	No	SF_exit<12	SF_adm>=12	
No	No	No	No	No	No	SF_exit<12	SF_adm>=12	
Yes	No			No	No	SF_exit<12	SF_adm<12	
No	No			No	No	SF_exit<12	SF_adm>=12	
Yes	Yes			Yes	Yes	SF_exit<12	SF_adm<12	
No	No	No	No	No	No	SF_exit<12	SF_adm>=12	
Yes	Yes			No	No	SF_exit<12	SF_adm>=12	
Yes	Yes	Yes	Yes	No	No	SF_exit<12	SF_adm<12	
Yes	Yes			No	No	SF_exit<12	SF_adm<12	
No	No	No	No	Yes	Yes	SF_exit<12	SF_adm>=	moderate
Yes	Yes			Yes	Yes	SF_exit<12	SF_adm<12	
No	No			No	No	SF_exit<12	SF_adm>=12	
Yes	Yes			Yes	Yes	SF_exit<12	SF_adm<12	
							SF_adm>=12	
No	No	No	No	No	No	SF_exit<12	SF_adm>=	moderate
No	No	No	No	Yes	Yes	SF_exit<12	SF_adm>=	marked de
							SF_adm>=12	
Yes	Yes	No	No	Yes	Yes	SF_exit<12	SF_adm<1	adequate
Yes	No	No	Yes	Yes	Yes	SF_exit<12	SF_adm>=	moderate
No	No	No	No	No	No	SF_exit<12	SF_adm>=12	
No	No	No	No	No	No	SF_exit<12	SF_adm>=	moderate
No	No	No	No	No	No	SF_exit<12	SF_adm>=	marked de
No	No	No	No	Yes	Yes	SF_exit<12	SF_adm>=12	
							SF_adm>=12	
Yes	No	No	No	No	No	SF_exit<12	SF_adm>=	moderate
No	No	No	No	No	No	SF_exit<12	SF_adm>=	marked de
Yes	Yes	Yes	Yes	Yes	Yes	SF_exit<12	SF_adm<1	marked de
Yes	Yes	No	No	No	No	SF_exit<12	SF_adm>=	marked de
Yes	Yes			Yes	Yes	SF_exit<12	SF_adm>=	moderate
No	No	No	No	No	No	SF_exit<12	SF_adm>=	adequate
No	No	No	No	No	No	SF_exit<12	SF_adm>=	moderate
No	No			No	No	SF_exit<12	SF_adm>=	severe def
No	No	No	No	Yes	Yes	SF_exit<12	SF_adm>=	marked de
Yes	Yes	No	No	Yes	Yes	SF_exit<12	SF_adm<1	marked de
Yes	No	No	No	No	No	SF_exit<12	SF_adm<1	moderate
No	No	No	No	Yes	Yes	SF_exit<12	SF_adm>=	moderate
Yes	Yes	No	No	No	Yes	SF_exit<12	SF_adm<1	marked de
No	No	No	No	Yes	Yes	SF_exit<12	SF_adm>=	moderate
No	No			No	No	SF_exit<12	SF_adm>=12	
Yes	Yes			Yes	Yes	SF_exit<12	SF_adm<12	
No	No			No	No	SF_exit<12	SF_adm>=12	
No	No	No	No	Yes	Yes	SF_exit<12	SF_adm>=	adequate

No	No	No	No	Yes	Yes	SF_exit<12	SF_adm>=12
No	No			Yes	Yes	SF_exit<12	SF_adm>= severe def
Yes	Yes			Yes	Yes	SF_exit<12	SF_adm<12
No	No	No	No	No	No	SF_exit<12	SF_adm>=12
Yes	Yes			Yes	Yes	SF_exit<12	SF_adm<12
No	No			Yes	Yes	SF_exit<12	SF_adm>=12
No	No	No	No	No	No	SF_exit<12	SF_adm>=12
No	No			No	No	SF_exit<12	SF_adm>=12
No	No	No	No	Yes	No	SF_exit<12	SF_adm>=12
No	No			Yes	Yes	SF_exit<12	SF_adm>=12
No	No	No	No	Yes	Yes	SF_exit<12	SF_adm>= adequate
No	No	No	No	Yes	Yes	SF_exit<12	SF_adm>=12
							SF_adm>=12
No	No	No	No	Yes	Yes	SF_exit<12	SF_adm>=12
Yes	Yes			Yes	Yes	SF_exit<12	SF_adm>=12
Yes	Yes	No	Yes	Yes	Yes	SF_exit<12	SF_adm>=12
Yes	Yes			Yes	Yes	SF_exit<12	SF_adm<12
No	No			No	No	SF_exit<12	SF_adm>=12
	Yes			Yes			SF_adm<12
	No			No			SF_adm>=12
No	No			Yes	Yes	SF_exit<12	SF_adm>=12
Yes	Yes	No	No	Yes	Yes	SF_exit<12	SF_adm>=12
No	No	No	No	No	No	SF_exit<12	SF_adm>=12
No	No	No	No	No	No	SF_exit<12	SF_adm>=12
No	No			No	No	SF_exit<12	SF_adm>=12
Yes	Yes	Yes	Yes	Yes	Yes	SF_exit<12	SF_adm<12
No	No	No	No	Yes	Yes	SF_exit<12	SF_adm>= moderate
No	Yes	No	No	Yes	Yes	SF_exit<12	SF_adm<12
No	Yes	No	No	No	No	SF_exit<12	SF_adm>= moderate
Yes	No	Yes	Yes	Yes	Yes	SF_exit<12	SF_adm>= moderate
Yes	Yes	Yes	Yes	Yes	Yes	SF_exit<12	SF_adm<12
Yes	Yes	No	No	No	No	SF_exit<12	SF_adm>=12
No	No			Yes	Yes	SF_exit<12	SF_adm>=12
No	No	No	No	No	No	SF_exit<12	SF_adm>=12
No	No	No	No	No	No	SF_exit<12	SF_adm>= marked de
Yes	Yes	Yes	Yes	Yes	Yes	SF_exit<12	SF_adm<12
Yes	Yes			Yes	Yes	SF_exit<12	SF_adm>=12
							SF_adm>=12
Yes	Yes			Yes	Yes	SF_exit<12	SF_adm<12
Yes	Yes	No	Yes	No	No	SF_exit<12	SF_adm>=12
Yes	Yes	Yes	Yes	Yes	Yes	SF_exit<12	SF_adm<12
Yes	Yes	Yes	Yes	Yes	Yes	SF_exit<12	SF_adm<12
Yes	Yes	No	No	Yes	Yes	SF_exit<12	SF_adm<12
No	No			Yes	Yes	SF_exit<12	SF_adm>=12
No	No	No	No	No	No	SF_exit<12	SF_adm>= moderate
No	Yes	No	No	Yes	Yes	SF_exit<12	SF_adm>=12
Yes	No	No	No	Yes	Yes	SF_exit<12	SF_adm>= severe def
							SF_adm>=12
Yes	Yes			Yes	Yes	SF_exit<12	SF_adm<12
Yes	Yes			Yes	Yes	SF_exit<12	SF_adm<12
Yes	Yes			Yes	Yes	SF_exit<12	SF_adm>=12

							SF_adm>=12	
No	No			No	No	SF_exit<12	SF_adm>=12	
Yes	No			Yes	Yes	SF_exit<12	SF_adm>=12	
Yes	Yes	No	No	Yes	Yes	SF_exit<12	SF_adm>=	moderate
Yes	No	No	No	Yes	Yes	SF_exit<12	SF_adm<1	marked de
No	No	No	No	No	No	SF_exit<12	SF_adm>=	moderate
Yes	Yes			No	No	SF_exit<12	SF_adm>=	adequate
No	No	No	No	Yes	Yes	SF_exit<12	SF_adm>=	adequate
No	No	No	No	No	No	SF_exit<12	SF_adm>=12	
No	No			No	No	SF_exit<12	SF_adm>=	marked de
Yes	Yes	Yes	Yes	Yes	Yes	SF_exit<12	SF_adm<1	severe defi
No	No			No	No	SF_exit<12	SF_adm>=12	
No	No	No	No	Yes	Yes	SF_exit<12	SF_adm>=12	
Yes	No	Yes	Yes	No	No	SF_exit<12	SF_adm<1	marked de
Yes	Yes	Yes	Yes	Yes	Yes	SF_exit<12	SF_adm<12	
No	No	No	No	Yes	Yes	SF_exit<12	SF_adm>=12	
Yes	Yes			Yes	Yes	SF_exit<12	SF_adm<12	
Yes	Yes	Yes	Yes	Yes	Yes	SF_exit<12	SF_adm<1	severe def
Yes	Yes			No	No	SF_exit<12	SF_adm<1	moderate
Yes	Yes	No	Yes	Yes	Yes	SF_exit<12	SF_adm>=	adequate
No	No	No	No	Yes	Yes	SF_exit<12	SF_adm>=	marked de
No	No	No	No	No	No	SF_exit<12	SF_adm>=	severe def
No	No	No	No	No	No	SF_exit<12	SF_adm>=	severe def
Yes	Yes	Yes	Yes	Yes	Yes	SF_exit<12	SF_adm<1	marked de
No	No	No	No	Yes	Yes	SF_exit<12	SF_adm>=12	
Yes	Yes	No	No	No	No	SF_exit<12	SF_adm>=12	
Yes	Yes	No	No	No	No	SF_exit<12	SF_adm<1	severe def
Yes	No	Yes	Yes	Yes	Yes	SF_exit<12	SF_adm>=	adequate
No	No	No	No	No	No	SF_exit<12	SF_adm>=	moderate
Yes	No	Yes	Yes	Yes	Yes	SF_exit<12	SF_adm>=	severe def
Yes	Yes	No	No	No	No	SF_exit<12	SF_adm<12	
No	No	No	No	No	No	SF_exit<12	SF_adm>=12	
No	No			Yes	Yes	SF_exit<12	SF_adm>=12	
No	No	No	No	No	No	SF_exit<12	SF_adm>=	moderate
No	No			No	No	SF_exit<12	SF_adm>=	moderate
No	No	No	No	No	No	SF_exit<12	SF_adm>=	moderate
Yes	Yes	Yes	Yes	No	No	SF_exit<12	SF_adm<1	adequate
No	No	No	No	Yes	Yes	SF_exit<12	SF_adm>=12	
Yes	No	No	No	Yes	Yes	SF_exit<12	SF_adm<1	moderate
Yes	Yes	Yes	Yes	Yes	Yes	SF_exit<12	SF_adm<1	moderate
Yes	Yes			No	No	SF_exit<12	SF_adm>=	moderate
No	No	No	No	No	No	SF_exit<12	SF_adm>=	moderate
Yes	Yes	No	No	Yes	Yes	SF_exit<12	SF_adm<1	moderate
No	No	No	No	Yes	Yes	SF_exit<12	SF_adm>=12	
No	No	No	No	No	No	SF_exit<12	SF_adm>=	marked de
No	No	No	No	No	No	SF_exit<12	SF_adm>=	moderate
Yes	Yes	No	No	Yes	Yes	SF_exit<12	SF_adm<1	severe def
No	No	No	No	No	No	SF_exit<12	SF_adm>=	adequate
No	No	No	No	No	No	SF_exit<12	SF_adm>=12	
Yes	Yes	No	Yes	No	No	SF_exit<12	SF_adm<1	moderate
No	No	No	No	No	No	SF_exit<12	SF_adm>=	moderate

Yes	Yes			Yes	Yes	SF_exit<12	SF_adm<12	
No	No	No	No	Yes	Yes	SF_exit<12	SF_adm>=	marked de
No	No	No	No	No	No	SF_exit<12	SF_adm>=	marked de
Yes	Yes	No	Yes	Yes	Yes	SF_exit<12	SF_adm>=	marked de
No	No	No	No	Yes	Yes	SF_exit<12	SF_adm>=	adequate
Yes	Yes	Yes	Yes	No	Yes	SF_exit<12	SF_adm>=	moderate
Yes	Yes	No	No	Yes	Yes	SF_exit<12	SF_adm>=	marked de
Yes	No	No	Yes	No	Yes	SF_exit<12	SF_adm>=	moderate
No	No	No	No	No	No	SF_exit<12	SF_adm>=	moderate
No	No	No	No	Yes	Yes	SF_exit<12	SF_adm>=	severe def
No	No	No	No	Yes	Yes	SF_exit<12	SF_adm>=	marked de
Yes	Yes	Yes	Yes	Yes	Yes	SF_exit<12	SF_adm<1	severe def
No	No	No	No	Yes	Yes	SF_exit<12	SF_adm>=	moderate
Yes	Yes	Yes	Yes	Yes	Yes	SF_exit<12	SF_adm<1	adequate
No	No	No	No	No	No	SF_exit<12	SF_adm>=	moderate
No	No			Yes	Yes	SF_exit<12	SF_adm>=	moderate
No	Yes	No	No	Yes	Yes	SF_exit<12	SF_adm>=	moderate
No	No	No	No	No	No	SF_exit<12	SF_adm>=	marked de
No	No	No	No	No	No	SF_exit<12	SF_adm>=	adequate
Yes	Yes	No	Yes	Yes	Yes	SF_exit<12	SF_adm>=	moderate
Yes	Yes	No	No	Yes	Yes	SF_exit<12	SF_adm<1	moderate
Yes	Yes	No	Yes	Yes	Yes	SF_exit<12	SF_adm>=	marked de
Yes	Yes			Yes	Yes	SF_exit<12	SF_adm<12	
No	No	No	No	No	No	SF_exit<12	SF_adm>=	moderate
No	No			No	No	SF_exit<12	SF_adm>=12	
Yes	Yes	No	Yes	Yes	Yes	SF_exit<12	SF_adm>=	moderate
Yes	Yes			Yes	Yes	SF_exit<12	SF_adm<12	
Yes	Yes			Yes	Yes	SF_exit<12	SF_adm<12	
Yes	Yes	Yes	Yes	Yes	Yes	SF_exit<12	SF_adm<1	moderate
Yes	Yes			Yes	Yes	SF_exit<12	SF_adm<12	
Yes	Yes			No	No	SF_exit<12	SF_adm<1	severe defi
No	No			No	No	SF_exit<12	SF_adm>=	marked de
Yes	Yes	No	Yes	Yes	Yes	SF_exit<12	SF_adm>=	moderate
Yes	Yes	Yes	Yes	No	No	SF_exit<12	SF_adm<1	moderate
Yes	Yes	Yes	Yes	Yes	Yes	SF_exit<12	SF_adm<12	
Yes	Yes	No	No	No	No	SF_exit<12	SF_adm<1	moderate
Yes	Yes	Yes	Yes	Yes	Yes	SF_exit<12	SF_adm>=	severe defi
Yes	Yes	Yes	Yes	Yes	Yes	SF_exit<12	SF_adm<1	moderate
Yes	No			No	No	SF_exit<12	SF_adm>=12	
Yes	No	No	No	No	No	SF_exit<12	SF_adm<1	marked de
Yes	Yes	Yes	Yes	Yes	Yes	SF_exit<12	SF_adm<1	moderate
Yes	Yes			Yes	Yes	SF_exit<12	SF_adm<12	
Yes	Yes	No	No	Yes	Yes	SF_exit<12	SF_adm>=	moderate
Yes	Yes			Yes	Yes	SF_exit<12	SF_adm<12	
No	No	No	No	Yes	Yes	SF_exit<12	SF_adm>=	moderate
No	No	No	No	No	No	SF_exit<12	SF_adm>=	severe def
Yes	No			Yes	Yes	SF_exit<12	SF_adm>=	adequate
Yes	Yes	No	Yes	No	No	SF_exit<12	SF_adm>=	severe def
Yes	Yes			Yes	Yes	SF_exit<12	SF_adm<1	adequate
Yes	No			Yes	Yes	SF_exit<12	SF_adm>=12	
Yes	Yes	Yes	Yes	Yes	Yes	SF_exit<12	SF_adm<12	

Yes	Yes	Yes	Yes	Yes	Yes	SF_exit<12	SF_adm<1	marked de
Yes	No	No	No	No	No	SF_exit<12	SF_adm<12	
Yes	Yes			Yes	Yes	SF_exit<12	SF_adm>=12	
	Yes			Yes			SF_adm>=12	
Yes	Yes	Yes	Yes	Yes	Yes	SF_exit<12	SF_adm<12	
	Yes			Yes			SF_adm<12	
Yes	No			No	No	SF_exit<12	SF_adm<12	
							SF_adm>=12	
No	No			Yes	Yes	SF_exit<12	SF_adm>=12	
Yes	No			No	No	SF_exit<12	SF_adm>=12	
Yes	Yes			No	No	SF_exit<12	SF_adm>=12	
No	No	No	No	No	No	SF_exit<12	SF_adm>=12	
No	No	No	No	No	No	SF_exit<12	SF_adm>=12	
Yes	Yes	No	No	Yes	Yes	SF_exit<12	SF_adm<12	
							SF_adm>=12	
No	No	No	No	Yes	No	SF_exit<12	SF_adm>=	moderate
Yes	Yes			No	No	SF_exit<12	SF_adm>=12	
Yes	Yes			No	No	SF_exit<12	SF_adm>=12	
Yes	No			Yes	Yes	SF_exit<12	SF_adm<12	
No	No	No	No	No	No	SF_exit<12	SF_adm>=	moderate
Yes	Yes			Yes	Yes	SF_exit<12	SF_adm>=12	
No	No	No	No	Yes	Yes	SF_exit<12	SF_adm>=	moderate
No	No	No	No	Yes	Yes	SF_exit<12	SF_adm>=12	
No	No			No	No	SF_exit<12	SF_adm>=12	
Yes	Yes	Yes	Yes	Yes	Yes	SF_exit<12	SF_adm<1	marked de
No	No	No	No	Yes	Yes	SF_exit<12	SF_adm>=	adequate
No	No	No	No	Yes	Yes	SF_exit<12	SF_adm>=12	
Yes	No	No	No	No	No	SF_exit<12	SF_adm>=12	
No	No			Yes	Yes	SF_exit<12	SF_adm>=12	
No	No	No	No	No	No	SF_exit<12	SF_adm>=	marked de
Yes	Yes	Yes	Yes	No	No	SF_exit<12	SF_adm<1	adequate
No	No	No	No	Yes	Yes	SF_exit<12	SF_adm>=	moderate
Yes	Yes	No	Yes	Yes	Yes	SF_exit<12	SF_adm>=	moderate
No	No	No	No	Yes	Yes	SF_exit<12	SF_adm>=12	
No	No	No	No	No	No	SF_exit<12	SF_adm>=	marked de
Yes	No	Yes	Yes	Yes	Yes	SF_exit<12	SF_adm<12	
No	No	No	No	Yes	Yes	SF_exit<12	SF_adm>=	marked de
No	No	No	No	No	No	SF_exit<12	SF_adm>=	adequate
Yes	Yes	No	Yes	No	No	SF_exit<12	SF_adm>=	severe def
No	No	No	No	No	No	SF_exit<12	SF_adm>=12	
No	No			Yes	Yes	SF_exit<12	SF_adm>=	moderate
No	Yes	No	No	Yes	Yes	SF_exit<12	SF_adm>=	adequate
No	No			No	No	SF_exit<12	SF_adm>=12	
Yes	Yes	No	No	Yes	Yes	SF_exit<12	SF_adm>=	marked de
No	No	No	No	Yes	Yes	SF_exit<12	SF_adm>=	adequate
	Yes			Yes			SF_adm<12	
No	No	No	No	Yes	Yes	SF_exit<12	SF_adm>=	adequate
No	No	No	No	Yes	Yes	SF_exit<12	SF_adm>=	adequate
No	No			No	No	SF_exit<12	SF_adm>=	moderate
No	No	No	No	Yes	Yes	SF_exit<12	SF_adm>=	adequate
No	No	No	No	No	No	SF_exit<12	SF_adm>=	marked de

							SF_adm>=12	
No	No			No	No	SF_exit<12	SF_adm>=	marked de
No	No	No	No	No	No	SF_exit<12	SF_adm>=12	
No	No			No	No	SF_exit<12	SF_adm>=12	
No	No			Yes	Yes	SF_exit<12	SF_adm>=12	
Yes	Yes			Yes	Yes	SF_exit<12	SF_adm>=12	
Yes	Yes	Yes	Yes	Yes	Yes	SF_exit<12	SF_adm<12	
Yes	Yes			Yes	Yes	SF_exit<12	SF_adm<12	
Yes	Yes	Yes	Yes	Yes	Yes	SF_exit<12	SF_adm<1	marked de
Yes	No	No	No	No	No	SF_exit<12	SF_adm>=	moderate
No	No			Yes	Yes	SF_exit<12	SF_adm>=12	
Yes	Yes	Yes	Yes	No	No	SF_exit<12	SF_adm<12	
No	No	No	No	No	No	SF_exit<12	SF_adm>=12	
Yes	No			Yes	Yes	SF_exit<12	SF_adm<1	adequate
No	No			No	No	SF_exit<12	SF_adm>=12	
No	No			No	No	SF_exit<12	SF_adm>=12	
No	No	No	No	No	No	SF_exit<12	SF_adm>=12	
No	No	No	No	No	No	SF_exit<12	SF_adm>=	moderate
No	No	No	No	No	No	SF_exit<12	SF_adm>=	moderate
No	No	No	No	No	No	SF_exit<12	SF_adm>=	marked de
No	No			Yes	Yes	SF_exit<12	SF_adm>=12	
No	No	No	No	No	No	SF_exit<12	SF_adm>=	marked de
No	No	No	No	Yes	Yes	SF_exit<12	SF_adm>=	adequate
Yes	No	No	No	No	No	SF_exit<12	SF_adm>=	severe def
Yes	No	No	No	No	No	SF_exit<12	SF_adm<1	moderate
No	No	No	No	No	No	SF_exit<12	SF_adm>=	moderate
No	No	No	No	No	No	SF_exit<12	SF_adm>=	moderate
Yes	Yes	No	No	Yes	Yes	SF_exit<12	SF_adm<12	
Yes	Yes	Yes	Yes	Yes	Yes	SF_exit<12	SF_adm<1	moderate
Yes	Yes	No	No	No	No	SF_exit<12	SF_adm<1	adequate
No	No			No	No	SF_exit<12	SF_adm>=12	
No	No	No	No	No	No	SF_exit<12	SF_adm>=	adequate
No	No	No	No	No	No	SF_exit<12	SF_adm>=	adequate
No	No			Yes	Yes	SF_exit<12	SF_adm>=	moderate
Yes	Yes			No	No	SF_exit<12	SF_adm>=12	
No	No	No	No	No	No	SF_exit<12	SF_adm>=	moderate
No	Yes	No	No	Yes	No	SF_exit<12	SF_adm<1	adequate

B12_statu	B12_cat_a	B12_cat_e	malaria_ad	ari_adm	diar_adm	fever_adm	malaria_ex	ari_exit
			Yes	No	No	No	No	No
			Yes	No	No	No	No	No
			Yes	No	No	No	No	No
adequate	adequate (	adequate (	No	No	No	No	No	No
			No	Yes	No	No	No	No
			No	Yes	No	No	No	No
			No	No	No	Yes	No	No
			No	No	Yes	Yes	No	No
			No	No	Yes	No	No	No
			No	Yes	Yes	Yes	No	Yes
			No	No	No	No	No	No
			No	No	Yes	No	No	Yes
adequate	adequate (	adequate (	No	No	No	Yes	No	No
			No	No	No	No	No	No
			Yes	No	No	Yes	No	No
			Yes	Yes	No	Yes	No	No
			No	No	No	No	No	No
			No	No	No	No	No	No
			No	Yes	No	No	No	No
			No	No	No	No	No	No
			No	Yes	No	Yes	No	No
moderate	Marked or	adequate (	Yes	No	No	No	No	No
moderate	adequate (	adequate (	No	No	Yes	No	No	No
			No	No	No	No	No	No
			No	Yes	No	Yes	No	No
moderate	Marked or	adequate (	No	No	No	Yes	No	No
moderate	adequate (	adequate (	No	Yes	No	No	No	No
moderate	adequate (	adequate (	Yes	No	Yes	Yes	No	No
adequate	adequate (	adequate (	No	No	Yes	No	No	No
marked de	Marked or	Marked or	No	No	No	No	Yes	Yes
moderate	Marked or	adequate (	No	No	Yes	No	No	No
			No	No	Yes	No	No	No
adequate	adequate (	adequate (	No	Yes	Yes	No	No	No
adequate	adequate (	adequate (	No	No	Yes	No	No	No
moderate	Marked or	adequate (	No	No	No	No	No	No
adequate	adequate (	adequate (	No	No	No	No	No	No
moderate	adequate (	adequate (	No	No	No	No	No	No
moderate	Marked or	adequate (	No	No	No	No	No	No
moderate	adequate (	adequate (	Yes	No	No	No	Yes	No
adequate	Marked or	adequate (	No	No	No	No	No	No
adequate	adequate (	adequate (	No	No	No	Yes	No	No
moderate	adequate (	adequate (	No	No	Yes	Yes	No	No
			No	Yes	Yes	No	No	No
			No	No	No	No	No	No
			No	No	Yes	Yes	No	No
adequate	adequate (	adequate (	Yes	No	No	No	No	No
moderate	adequate (	adequate (	Yes	No	No	Yes	No	No
marked de	adequate (	Marked or	Yes	No	No	No	No	No
adequate	adequate (	adequate (	Yes	No	No	No	No	No
moderate	adequate (	adequate (	Yes	Yes	Yes	No	No	No

moderate	Marked or adequate (		No	No	No	No	No	No
moderate	Marked or adequate (		Yes	No	No	No	No	No
moderate	adequate (	adequate (	Yes	No	No	No	No	No
			Yes	Yes	No	Yes	No	No
moderate	Marked or adequate (		No	No	No	No	No	No
moderate	adequate (	adequate (	No	No	No	Yes	No	No
			No	No	No	No	No	No
adequate	adequate (	adequate (	No	No	No	No	No	No
			No	No	Yes	No	No	Yes
			No	No	No	No	No	No
moderate	Marked or adequate (		No	No	No	No	No	No
moderate	adequate (	adequate (	No	No	No	No	No	No
			No	Yes	No	No	No	No
adequate	adequate (	adequate (	Yes	No	No	Yes	No	No
			No	Yes	Yes	Yes	No	No
			No	Yes	No	No	No	No
adequate	adequate (	adequate (	No	No	Yes	No	No	No
			No	No	No	No	No	No
moderate	adequate (	adequate (	No	No	No	No	No	No
moderate	adequate (	adequate (	No	No	Yes	Yes	No	No
adequate	adequate (	adequate (	No	No	Yes	No	No	No
moderate	Marked or adequate (		No	No	No	No	No	No
adequate	adequate (	adequate (	No	No	Yes	No	No	No
moderate	adequate (	adequate (	No	No	No	No	No	No
moderate	adequate (	adequate (	No	No	No	No	No	No
			Yes	No	No	No	No	Yes
moderate	adequate (	adequate (	No	No	No	Yes	No	No
			No	No	No	No	No	No
moderate	adequate (	adequate (	No	No	Yes	No	No	No
adequate	adequate (	adequate (	No	No	No	No	No	No
			No	No	No	No	No	No
			No	No	No	No	No	No
			No	No	Yes	No	No	No
			No	No	No	Yes	No	No
			No	Yes	No	No	No	No
			No	Yes	No	No	No	No
			No	No	No	No	No	Yes
			No	No	No	Yes	No	No
			No	No	Yes	No	No	No
			No	Yes	No	No	No	No
			No	Yes	Yes	No	No	No
moderate	Marked or adequate (		No	No	Yes	No	No	No
adequate	Marked or adequate (		No	Yes	No	No	No	No
			Yes	No	Yes	No	Yes	No
marked de	adequate (	Marked or	Yes	Yes	Yes	No	No	No
			No	No	Yes	No	No	No
			Yes	No	No	No	No	No
adequate	Marked or adequate (		No	No	Yes	No	No	No
			No	Yes	Yes	No	No	No
			No	No	Yes	No	No	No
adequate	adequate (	adequate (	No	Yes	Yes	No	No	No

			No	No	Yes	No	No	No
moderate	adequate (	adequate (	Yes	Yes	No	No	No	No
moderate	adequate (	adequate (	No	No	No	No	No	No
			Yes	No	No	No	No	No
moderate	Marked or	adequate (	No	No	No	No	No	No
			No	No	No	No	No	No
moderate	adequate (	adequate (	No	No	Yes	No	No	No
adequate	adequate (	adequate (	No	Yes	No	No	No	No
adequate	adequate (	adequate (	No	Yes	No	No	No	No
moderate	adequate (	adequate (	Yes	No	Yes	No	No	No
			No	No	No	No	No	No
adequate	adequate (	adequate (	No	Yes	No	No	No	No
			No	No	Yes	No	No	No
moderate	adequate (	adequate (	No	Yes	Yes	No	No	No
adequate	adequate (	adequate (	No	Yes	Yes	No	No	No
			No	No	Yes	No	No	No
			No	No	No	No	No	No
moderate	adequate (	adequate (	No	Yes	No	No	No	No
moderate	adequate (	adequate (	No	No	No	No	No	No
			No	Yes	No	No	No	No
moderate	Marked or	adequate (	No	No	Yes	Yes	No	No
deficiency	adequate or moderat		Yes	No	No	No	No	No
moderate	adequate (	adequate (	No	No	No	Yes	No	No
			No	No	No	Yes	No	No
			Yes	No	No	Yes	No	No
			No	Yes	No	Yes	No	No
adequate	adequate (	adequate (	No	No	No	No	No	No
			No	No	No	No	No	No
moderate	Marked or	adequate (	No	No	No	No	No	No
moderate	adequate (	adequate (	No	No	Yes	Yes	No	No
			No	No	No	Yes	No	No
			No	No	No	No	No	No
adequate	adequate (	adequate (	No	Yes	No	Yes	No	No
			No	No	No	Yes	No	No
marked de	Marked or	Marked or	No	No	No	Yes	No	No
moderate	adequate (	adequate (	Yes	No	No	Yes	No	No
adequate	adequate (	adequate (	Yes	No	Yes	No	No	No
			No	No	No	No	No	No
			No	No	No	Yes	No	No
			No	No	No	No	No	No
marked de	Marked or	Marked or	No	No	No	No	No	No
moderate	Marked or	adequate (	Yes	No	No	No	No	No
adequate	adequate (	adequate (	Yes	Yes	No	Yes	Yes	Yes
			Yes	Yes	Yes	Yes	No	No
			Yes	No	No	Yes	No	No
moderate	Marked or	adequate (	No	No	Yes	No	No	No
adequate	adequate (	adequate (	Yes	No	Yes	No	No	No
			Yes	No	Yes	Yes	No	No
			Yes	Yes	Yes	No	No	No
			Yes	Yes	No	No	No	No
			No	Yes	No	No	No	No

			Yes	Yes	No	No	No	No
			No	No	No	No	No	No
			No	No	No	No	No	No
			No	Yes	Yes	No	No	Yes
			No	Yes	Yes	No	No	No
			No	Yes	No	No	No	No
			Yes	Yes	No	No	No	No
			No	No	No	No	No	No
			No	No	No	Yes	No	No
			No	No	No	No	No	Yes
			No	No	No	No	No	No
			Yes	No	No	No	No	No
			No	Yes	No	No	No	No
			No	Yes	No	No	No	No
			Yes	Yes	No	Yes	No	No
			No	No	Yes	Yes	No	No
			No	Yes	No	No	No	No
			No	No	No	No	No	No
			No	Yes	No	No	No	No
			No	Yes	No	No	No	No
			No	Yes	No	No	No	No
			No	Yes	No	Yes	No	No
			No	No	No	Yes	No	No
			No	No	Yes	No	No	No
			No	Yes	No	No	No	No
moderate	Marked or adequate		No	Yes	No	Yes	No	No
			No	No	No	Yes	No	No
			No	No	No	No	No	No
			No	Yes	No	No	No	No
			No	Yes	Yes	No	Yes	Yes
			No	No	No	No	No	No
			Yes	No	Yes	No	No	No
moderate	Marked or adequate		No	No	No	No	No	No
			No	No	No	No	No	No
			No	No	No	No	No	No
moderate	Marked or adequate		Yes	Yes	No	Yes	No	No
			Yes	No	No	No	No	No
			Yes	No	No	No	No	No
moderate	adequate	adequate	Yes	Yes	No	No	No	No
moderate	adequate	adequate	No	Yes	No	No	No	No
			No	Yes	No	No	No	No
moderate	Marked or adequate		Yes	No	No	No	No	No
moderate	Marked or adequate		Yes	No	No	No	No	No
			Yes	No	No	No	No	No
moderate	adequate	adequate	No	Yes	No	No	No	No
			Yes	Yes	No	No	No	No
moderate	adequate	adequate	Yes	No	No	Yes	No	No
			Yes	Yes	No	No	No	No
			No	Yes	No	No	No	No
moderate	adequate	adequate	Yes	Yes	No	No	No	Yes
			No	No	No	Yes	No	No

adequate	adequate (	adequate (	No	No	Yes	No	No	No
adequate	adequate (	adequate (	Yes	Yes	Yes	Yes	No	No
			No	Yes	No	No	No	No
moderate	adequate (	adequate (	Yes	Yes	Yes	No	No	No
marked de	Marked or	Marked or	No	Yes	Yes	No	No	No
moderate	adequate (	adequate (	Yes	No	No	Yes	No	No
moderate	Marked or	adequate (	No	No	Yes	No	No	No
			Yes	No	No	No	No	No
			No	Yes	No	No	Yes	No
adequate	adequate (	adequate (	No	Yes	No	No	Yes	No
			Yes	Yes	No	No	No	Yes
			Yes	Yes	No	Yes	No	No
			Yes	No	No	Yes	No	No
			Yes	No	Yes	Yes	Yes	Yes
			Yes	Yes	No	No	No	No
			Yes	Yes	Yes	No	No	No
moderate	adequate (	adequate (	No	No	Yes	Yes	No	No
			No	No	No	Yes	No	No
			Yes	No	No	No	No	No
			Yes	Yes	Yes	Yes	No	No
			No	Yes	Yes	No	No	No
			No	No	No	No	No	No
			No	Yes	Yes	No	No	No
			Yes	Yes	No	No	No	No
			No	Yes	No	No	No	No
			Yes	Yes	No	Yes	No	Yes
			No	Yes	Yes	Yes	No	No
			No	Yes	No	No	No	No
			No	Yes	Yes	Yes	No	No
			No	Yes	No	No	No	No
			No	No	No	Yes	No	No
			No	Yes	Yes	No	No	No
			No	Yes	No	No	No	No
			No	Yes	Yes	No	No	Yes
			No	Yes	No	Yes	No	No
			No	No	No	No	No	No
			No	No	No	Yes	No	No
			No	Yes	No	No	No	No
			No	Yes	No	Yes	No	No
			Yes	No	No	No	No	Yes
			No	Yes	No	No	No	No
			No	Yes	Yes	No	No	No
			No	Yes	Yes	No	No	No
			No	Yes	Yes	No	No	No
			No	No	No	Yes	No	No
			No	Yes	No	Yes	No	Yes
adequate	adequate (	adequate (	No	Yes	No	No	No	No
			No	Yes	No	Yes	No	No
marked de	Marked or	Marked or	No	Yes	No	No	No	No
			No	Yes	No	Yes	No	No
			No	Yes	No	Yes	No	No

			No	Yes	Yes	No	No	Yes
moderate	adequate (	adequate (	No	No	Yes	No	No	No
			No	Yes	No	No	No	No
moderate	Marked or	adequate (	No	No	No	No	No	No
			No	Yes	No	Yes	No	No
			No	Yes	No	No	No	No
			No	No	No	No	No	Yes
moderate	adequate (	adequate (	Yes	No	Yes	Yes	No	No
adequate	adequate (	adequate (	No	No	No	No	No	No
			Yes	No	No	No	No	No
adequate	Marked or	adequate (	No	Yes	No	No	No	No
adequate	adequate (	adequate (	No	No	Yes	No	No	No
marked de	Marked or	Marked or	No	No	No	No	No	No
moderate	adequate (	adequate (	Yes	Yes	Yes	No	No	No
adequate	adequate (	adequate (	Yes	Yes	No	No	No	No
			No	No	No	No	No	No
moderate	Marked or	adequate (	No	No	No	No	No	No
adequate	adequate (	adequate (	No	No	Yes	No	No	Yes
moderate	adequate (	adequate (	No	No	No	No	No	No
marked de	adequate (	Marked or	Yes	Yes	No	No	No	No
moderate	adequate (	adequate (	Yes	No	No	No	No	No
			No	Yes	No	Yes	No	No
moderate	adequate (	adequate (	No	No	No	No	No	No
marked de	Marked or	Marked or	No	Yes	No	No	No	No
adequate	adequate (	adequate (	No	No	Yes	No	No	No
moderate	adequate (	adequate (	No	Yes	No	No	No	No
moderate	Marked or	adequate (	Yes	No	No	Yes	No	No
			Yes	No	No	Yes	No	No
			Yes	No	No	No	No	No
adequate	adequate (	adequate (	Yes	No	No	No	No	No
moderate	adequate (	adequate (	No	No	No	No	No	No
moderate	Marked or	adequate (	Yes	No	No	No	No	No
moderate	Marked or	adequate (	Yes	Yes	No	No	No	No
moderate	adequate (	adequate (	Yes	Yes	No	No	No	No
adequate	adequate (	adequate (	No	No	No	No	No	No
moderate	Marked or	adequate (	Yes	Yes	No	No	No	Yes
moderate	adequate (	adequate (	Yes	Yes	No	Yes	No	No
moderate	adequate (	adequate (	Yes	No	No	Yes	No	No
			Yes	No	Yes	Yes	No	No
adequate	adequate (	adequate (	Yes	No	No	No	No	No
adequate	adequate (	adequate (	Yes	No	No	No	No	No
adequate	adequate (	adequate (	No	Yes	No	Yes	No	No
adequate	adequate (	adequate (	Yes	No	No	No	No	No
			Yes	No	No	No	No	No
moderate	Marked or	adequate (	Yes	Yes	No	No	No	No
moderate	adequate (	adequate (	Yes	Yes	No	Yes	No	No
moderate	adequate (	adequate (	No	Yes	No	No	No	No
adequate	adequate (	adequate (	No	No	No	Yes	No	No
			No	Yes	No	No	Yes	No
deficiency	adequate or moderat		Yes	Yes	No	No	No	No
moderate	adequate (	adequate (	No	Yes	No	No	No	No

adequate	Marked or adequate (		No	Yes	No	No	No	No
			No	Yes	No	No	No	No
			No	No	No	No	No	No
deficiency	adequate or moderat		Yes	No	No	Yes	No	No
moderate	Marked or adequate (		No	No	No	No	No	No
			Yes	No	Yes	No	No	No
moderate	Marked or adequate (		Yes	No	No	No	No	No
adequate	adequate (	adequate (	No	No	No	Yes	No	No
marked de	Marked or	Marked or	Yes	Yes	No	Yes	No	No
moderate	adequate (	adequate (	Yes	No	No	Yes	No	No
moderate	adequate (	adequate (	Yes	No	No	Yes	No	No
adequate	adequate (	adequate (	No	Yes	No	No	No	No
moderate	adequate (	adequate (	No	No	No	No	No	No
adequate	adequate (	adequate (	No	No	Yes	No	No	No
adequate	adequate (	adequate (	Yes	No	No	Yes	No	No
moderate	Marked or adequate (		No	No	No	No	No	No
			No	No	Yes	No	No	No
			Yes	No	No	No	No	No
severe def	Marked or	Marked or	No	No	No	No	No	No
			No	No	No	Yes	No	No
			Yes	No	No	Yes	No	No
adequate	adequate (	adequate (	No	No	No	No	No	No
moderate	adequate (	adequate (	No	Yes	No	No	No	No
			Yes	No	No	No	Yes	No
moderate	adequate (	adequate (	No	No	No	No	No	No
ficiency	Marked or severate		No	No	No	No	No	No
			Yes	Yes	No	Yes	No	No
			Yes	Yes	Yes	Yes	No	No
			No	Yes	No	No	No	No
			No	No	No	No	No	No
			Yes	Yes	No	No	No	No
			No	Yes	No	No	No	No
			No	Yes	No	No	No	No
			Yes	Yes	No	No	No	No
			Yes	Yes	No	No	No	No
			No	Yes	No	No	No	No
			No	Yes	No	Yes	No	No
			Yes	Yes	No	No	No	No
			No	Yes	Yes	No	No	No
			No	Yes	No	No	No	No
			No	Yes	Yes	Yes	Yes	No
moderate	Marked or adequate (		No	Yes	No	No	No	No
adequate	adequate (	adequate (	No	No	No	No	No	No
			No	No	No	No	No	No
marked de	Marked or	Marked or	Yes	Yes	No	No	No	No
			Yes	No	Yes	No	No	No
adequate	adequate (	adequate (	Yes	No	Yes	Yes	No	No
			Yes	Yes	No	No	No	No
moderate	Marked or adequate (		No	Yes	No	No	No	No
moderate	adequate (	adequate (	No	Yes	No	No	No	No
adequate	adequate (	adequate (	No	Yes	No	No	No	No

			Yes	Yes	No	No	No	No
moderate	adequate (	adequate (	Yes	No	No	Yes	No	No
			Yes	Yes	No	No	No	No
adequate	adequate (	adequate (	Yes	Yes	No	No	No	No
			Yes	No	No	No	No	No
moderate	adequate (	adequate (	Yes	Yes	No	Yes	No	No
			No	Yes	No	No	No	No
			No	Yes	No	No	No	No
marked de	Marked or	Marked or	No	No	No	No	No	No
moderate	Marked or	adequate (	No	Yes	No	No	No	No
adequate	adequate (	adequate (	No	Yes	No	No	No	No
moderate	adequate (	adequate (	No	No	Yes	No	No	No
ficiency	Marked or severate		No	Yes	No	No	No	Yes
moderate	Marked or	adequate (	No	No	No	No	No	No
moderate	adequate (	adequate (	Yes	No	No	Yes	No	No
			Yes	Yes	No	No	No	No
			Yes	No	No	Yes	No	No
adequate	adequate (	adequate (	No	No	No	No	No	No
moderate	adequate (	adequate (	Yes	No	Yes	No	No	No
adequate	adequate (	adequate (	Yes	No	No	Yes	No	No
			No	No	No	No	No	No
			No	No	No	No	No	No
			No	No	No	No	No	No
			No	No	No	No	No	No
			No	No	Yes	No	No	No
moderate	adequate (	adequate (	Yes	No	No	No	No	No
marked de	Marked or	Marked or	Yes	No	Yes	Yes	No	No
			Yes	No	No	No	No	No
marked de	adequate (	Marked or	No	No	No	No	Yes	Yes
			No	No	No	No	No	No
adequate	adequate (	adequate (	Yes	Yes	No	No	No	No
moderate	Marked or	adequate (	Yes	No	No	No	No	No
			No	Yes	No	Yes	No	No
			Yes	No	No	Yes	No	No
			Yes	No	Yes	Yes	No	No
			Yes	Yes	No	No	No	No
			No	Yes	No	No	No	No
			No	No	Yes	No	No	No
			Yes	Yes	No	Yes	No	No
			No	No	No	No	No	No
			Yes	Yes	Yes	Yes	No	No
			No	Yes	No	No	No	No
			No	Yes	No	No	No	No
			Yes	Yes	No	No	No	No
			No	Yes	Yes	No	No	No
			Yes	Yes	Yes	Yes	No	No
			No	Yes	No	No	No	No
			No	No	No	No	No	No
moderate	adequate (	adequate (	No	No	No	No	No	No
			No	No	No	No	No	No
			No	No	No	No	No	No

			No	No	No	No	No	No
moderate (	adequate (	adequate (	No	Yes	No	No	No	No
marked de	adequate (	Marked or	Yes	Yes	No	No	No	No
deficiency	adequate or moderat		No	No	Yes	No	No	No
			Yes	No	Yes	Yes	No	No
			No	No	Yes	No	No	No
			No	Yes	No	No	Yes	Yes
			No	No	No	No	No	No
moderate (	Marked or	adequate (	Yes	No	Yes	No	No	No
moderate (	Marked or	adequate (	No	No	No	No	Yes	No
moderate (	adequate (	adequate (	No	Yes	Yes	No	No	No
moderate (	adequate (	adequate (	No	No	No	No	No	No
moderate (	Marked or	adequate (	Yes	No	No	No	No	No
			Yes	Yes	No	No	No	No
moderate (	adequate (	adequate (	No	Yes	No	No	No	No
moderate (	adequate (	adequate (	No	Yes	No	No	No	No
adequate	adequate (	adequate (	Yes	No	No	Yes	No	No
			Yes	No	No	No	No	No
	adequate or moderat		No	No	No	No	No	No
			Yes	No	No	Yes	No	No
adequate	adequate (	adequate (	No	No	No	No	No	No
marked de	Marked or	Marked or	No	No	No	No	No	No
moderate (	Marked or	adequate (	No	No	No	No	No	No
moderate (	adequate (	adequate (	Yes	Yes	No	Yes	No	No
moderate (	adequate (	adequate (	Yes	No	Yes	No	No	No
			No	No	No	No	No	No
adequate	adequate (	adequate (	No	No	No	No	No	No
			No	No	No	No	No	No
			No	No	No	No	No	No
			Yes	No	Yes	No	No	No
			Yes	No	No	Yes	No	No
adequate	adequate (	adequate (	Yes	Yes	Yes	Yes	No	No
marked de	Marked or	Marked or	No	No	No	No	No	No
moderate (	Marked or	adequate (	No	No	No	No	No	No
			No	No	No	No	No	No
moderate (	adequate (	adequate (	No	No	No	No	No	No
			No	Yes	No	Yes	No	No
			No	Yes	No	No	No	No
			No	Yes	No	No	No	No
			No	No	Yes	No	No	No
adequate	Marked or	adequate (	Yes	No	Yes	No	No	No
			No	Yes	No	Yes	No	No
			No	No	No	No	No	No
			No	No	No	No	No	No
moderate (	adequate (	adequate (	Yes	No	No	No	No	No
			No	No	No	No	No	No
			No	No	No	No	No	No
			No	No	No	No	No	No
			Yes	No	Yes	No	No	No
			Yes	Yes	No	No	No	No
			No	No	No	No	No	Yes

			No	Yes	No	No	No	No
			No	Yes	No	No	No	No
			No	Yes	No	No	No	No
			No	Yes	No	Yes	No	No
adequate	adequate (	adequate (	No	No	No	No	No	No
			No	No	No	No	No	No
			No	No	No	No	No	No
			No	No	No	No	No	No
moderate	adequate (	adequate (	No	No	No	No	No	No
adequate	adequate (	adequate (	No	No	No	No	No	No
adequate	adequate (	adequate (	No	No	No	No	No	No
			No	No	No	Yes	No	No
			No	No	No	No	No	No
moderate	Marked or	adequate (	Yes	No	No	No	No	No
			Yes	No	No	No	Yes	Yes
			No	No	Yes	No	No	No
			Yes	No	No	Yes	No	No
adequate	Marked or	adequate (	Yes	No	No	No	No	No
adequate	Marked or	adequate (	Yes	No	No	No	No	No
adequate	adequate (	adequate (	No	No	No	No	No	No
			No	Yes	No	No	No	No
			Yes	No	No	No	No	No
deficiency	adequate or moderate		Yes	Yes	No	Yes	No	Yes
moderate	adequate (	adequate (	Yes	No	Yes	No	No	No
			No	No	Yes	No	No	No
			No	No	No	No	No	No
			Yes	No	No	No	No	No
marked de	Marked or	Marked or	Yes	No	Yes	No	No	No
adequate	adequate (	adequate (	No	No	Yes	No	No	No
moderate	adequate (	adequate (	Yes	No	No	Yes	No	No
adequate	adequate (	adequate (	No	Yes	No	No	No	Yes
adequate	adequate (	adequate (	Yes	No	No	No	No	No
			No	Yes	No	No	No	No
			No	No	No	No	No	No
			No	Yes	No	No	No	No
			Yes	No	No	No	No	No
adequate	adequate (	adequate (	Yes	No	Yes	Yes	No	No
moderate	adequate (	adequate (	Yes	No	No	No	No	No
			Yes	No	No	Yes	No	No
adequate	adequate (	adequate (	No	Yes	No	No	No	No
			No	No	No	No	No	No
moderate	adequate (	adequate (	No	No	No	No	No	No
			No	No	No	No	No	No
			No	No	No	No	No	No
			No	No	No	No	No	No
			No	No	No	Yes	No	No
moderate	adequate (	adequate (	No	No	Yes	No	No	No
moderate	adequate (	adequate (	No	No	No	Yes	No	No
adequate	adequate (	adequate (	No	No	No	No	No	No
moderate	adequate (	adequate (	No	Yes	No	No	No	Yes
moderate	adequate (	adequate (	No	No	No	Yes	No	No

marked de	adequate (	Marked or	No	No	No	No	No	No
			No	No	Yes	No	No	No
			No	No	No	No	No	No
			No	Yes	No	Yes	No	Yes
			No	No	Yes	No	No	No
			No	Yes	No	No	No	No
			No	No	Yes	No	No	No
			No	No	No	No	No	No
			Yes	Yes	Yes	Yes	No	No
			No	Yes	Yes	No	No	No
			Yes	No	Yes	No	No	No
			No	No	No	No	No	No
			No	No	No	No	No	No
			No	No	No	No	No	No
			No	No	No	No	No	No
			No	No	No	No	No	No
			No	No	Yes	No	No	No
			No	Yes	No	Yes	No	No
adequate	adequate (	adequate (	No	No	No	No	No	No
			No	No	No	No	No	No
			No	Yes	No	No	No	No
			No	No	No	No	No	No
			No	No	No	No	No	No
moderate	adequate (	adequate (	No	Yes	No	No	No	No
moderate	Marked or adequate (		No	No	No	No	No	No
			No	No	No	No	No	No
adequate	adequate (	adequate (	No	No	No	No	No	No
adequate	adequate (	adequate (	No	Yes	Yes	No	Yes	No
			No	No	No	No	No	No
moderate	adequate (	adequate (	Yes	No	No	Yes	No	No
moderate	Marked or adequate (		Yes	No	No	No	No	No
			Yes	No	No	No	No	No
			Yes	No	No	Yes	Yes	Yes
adequate	adequate (	adequate (	No	No	No	No	No	No
moderate	Marked or adequate (		No	No	No	Yes	No	No
moderate	Marked or adequate (		No	Yes	No	No	No	No
moderate	Marked or adequate (		No	No	No	No	No	No
moderate	adequate (	adequate (	No	No	Yes	No	No	No
adequate	adequate (	adequate (	No	No	Yes	No	No	No
moderate	adequate (	adequate (	No	No	No	No	No	No
moderate	Marked or adequate (		No	No	Yes	No	No	No
moderate	Marked or adequate (		Yes	No	No	No	No	No
adequate	Marked or adequate (		No	No	No	No	No	No
moderate	adequate (	adequate (	No	No	No	No	No	No
adequate	adequate (	adequate (	Yes	No	No	No	No	No
moderate	Marked or adequate (		No	No	No	No	No	No
moderate	adequate (	adequate (	No	No	No	Yes	Yes	No
			No	No	No	No	No	No
			No	No	No	No	No	No
			Yes	Yes	No	No	No	No
adequate	adequate (	adequate (	Yes	Yes	No	No	No	No

			No	No	Yes	No	No	No
severe def	Marked or	Marked or	No	No	Yes	No	No	No
			No	No	No	No	No	No
			No	Yes	Yes	Yes	No	No
			Yes	No	Yes	No	No	No
			Yes	No	Yes	No	No	No
			Yes	No	No	Yes	No	No
			Yes	No	No	No	No	No
			Yes	No	No	Yes	No	No
			Yes	No	No	No	No	No
moderate	adequate	adequate	Yes	No	Yes	No	No	No
			Yes	No	No	Yes	No	No
			No	Yes	No	No	No	No
			Yes	No	Yes	Yes	No	No
			Yes	No	No	No	No	No
			No	No	Yes	No	No	No
			Yes	No	No	No	No	No
			Yes	No	No	No	No	No
			No	No	No	Yes	No	No
			No	No	Yes	No	No	No
			No	No	No	No	No	No
			Yes	No	Yes	No	No	No
			Yes	No	Yes	No	No	No
			No	No	Yes	No	No	No
			No	Yes	No	No	No	Yes
			No	No	Yes	No	No	No
moderate	adequate	adequate	Yes	Yes	Yes	Yes	No	No
			No	Yes	Yes	Yes	No	No
moderate	adequate	adequate	No	Yes	No	Yes	No	No
adequate	adequate	adequate	Yes	Yes	No	Yes	No	Yes
			No	No	Yes	No	No	No
			No	No	Yes	No	No	No
			Yes	No	No	Yes	No	No
			No	No	Yes	Yes	No	Yes
adequate	Marked or	adequate	No	No	No	Yes	No	No
			No	No	Yes	No	No	No
			No	Yes	Yes	Yes	No	No
			No	No	Yes	Yes	No	No
			Yes	No	Yes	Yes	No	No
			No	No	No	Yes	No	No
			No	No	No	Yes	No	No
			No	No	No	Yes	No	No
			No	No	No	Yes	No	No
			No	Yes	No	Yes	No	No
moderate	adequate	adequate	No	Yes	No	Yes	No	No
			No	No	No	No	No	No
marked de	Marked or	Marked or	Yes	Yes	No	No	No	No
			No	No	No	No	No	Yes
			No	No	No	No	No	No
			No	No	No	Yes	No	No
			No	No	No	Yes	No	No

			No	No	No	No	No	No
			No	No	No	Yes	No	No
			No	No	No	No	No	No
adequate	adequate (	adequate (	No	No	No	Yes	No	No
marked de	Marked or	Marked or	No	No	No	No	No	No
moderate	adequate (	adequate (	No	No	No	No	No	No
adequate	adequate (	adequate (	No	No	No	Yes	No	No
adequate	adequate (	adequate (	Yes	Yes	Yes	Yes	No	No
			No	No	Yes	No	No	No
moderate	Marked or	adequate (	Yes	Yes	Yes	Yes	No	Yes
iciency	Marked or severate		No	No	No	No	No	No
			No	Yes	No	Yes	No	No
			No	No	Yes	No	No	No
marked de	Marked or	Marked or	No	No	No	No	No	No
			No	No	No	No	No	No
			No	No	No	No	No	No
			No	No	No	No	Yes	No
marked de	Marked or	Marked or	No	No	Yes	No	No	No
moderate	adequate (	adequate (	No	No	Yes	No	No	No
adequate	adequate (	adequate (	No	No	Yes	No	No	No
moderate	Marked or	adequate (	No	Yes	No	No	No	No
severe def	Marked or	Marked or	No	No	No	Yes	No	No
moderate	Marked or	adequate (	Yes	No	Yes	Yes	No	No
moderate	Marked or	adequate (	No	No	No	No	No	No
			No	No	No	No	No	No
			No	No	No	No	No	No
moderate	Marked or	adequate (	No	No	Yes	Yes	No	No
adequate	adequate (	adequate (	No	No	Yes	No	No	No
adequate	adequate (	adequate (	Yes	No	No	No	No	No
moderate	Marked or	adequate (	No	No	No	Yes	No	No
			No	No	Yes	No	No	No
			Yes	No	No	No	No	No
			No	Yes	No	No	No	No
adequate	adequate (	adequate (	No	Yes	No	No	No	No
moderate	adequate (	adequate (	No	No	No	No	Yes	No
moderate	adequate (	adequate (	No	No	No	No	No	Yes
adequate	adequate (	adequate (	Yes	No	No	Yes	No	No
			Yes	No	No	No	No	No
moderate	adequate (	adequate (	Yes	No	No	No	No	Yes
adequate	adequate (	adequate (	No	No	No	No	Yes	No
moderate	adequate (	adequate (	Yes	No	No	Yes	Yes	No
moderate	adequate (	adequate (	Yes	No	No	Yes	No	No
moderate	adequate (	adequate (	No	No	Yes	Yes	No	No
			Yes	No	No	Yes	No	No
moderate	Marked or	adequate (	No	Yes	No	No	No	No
moderate	adequate (	adequate (	No	No	No	No	No	No
moderate	Marked or	adequate (	No	No	No	Yes	No	No
	adequate or moderat(		Yes	No	No	No	No	No
			Yes	No	No	No	No	No
moderate	adequate (	adequate (	No	No	No	No	No	No
adequate	adequate (	adequate (	Yes	Yes	No	Yes	No	No

		No	No	No	Yes	No	No
moderate	Marked or adequate (	Yes	No	Yes	No	No	No
adequate	Marked or adequate (	No	Yes	No	Yes	No	No
moderate	Marked or adequate (	Yes	No	No	No	No	No
adequate	adequate (adequate (	No	No	Yes	No	No	Yes
moderate	adequate (adequate (	Yes	No	No	Yes	No	No
moderate	Marked or adequate (	Yes	No	No	Yes	No	No
deficiency	adequate or moderat	No	No	No	No	No	No
moderate	adequate (adequate (	No	No	Yes	No	No	No
marked de	Marked or Marked or	Yes	No	No	No	No	Yes
adequate	Marked or adequate (	Yes	No	No	No	No	No
moderate	Marked or adequate (	No	No	No	No	No	Yes
moderate	adequate (adequate (	No	No	No	No	No	No
adequate	adequate (adequate (	Yes	No	Yes	No	No	No
adequate	adequate (adequate (	No	No	No	Yes	Yes	No
moderate	adequate (adequate (	No	No	No	No	Yes	No
moderate	adequate (adequate (	No	Yes	Yes	No	No	No
moderate	Marked or adequate (	No	No	No	No	No	No
adequate	adequate (adequate (	Yes	No	Yes	No	No	No
moderate	adequate (adequate (	Yes	Yes	No	No	No	No
moderate	adequate (adequate (	No	No	Yes	Yes	No	No
moderate	Marked or adequate (	Yes	No	No	Yes	No	No
		No	No	No	No	No	No
adequate	adequate (adequate (	No	No	No	No	No	No
		No	Yes	No	No	No	No
moderate	adequate (adequate (	No	No	Yes	No	No	No
		No	No	No	No	No	No
		No	No	No	No	No	No
adequate	adequate (adequate (	No	No	Yes	No	No	No
		No	No	No	Yes	No	No
iciency	Marked or severate	No	No	No	No	No	No
adequate	Marked or adequate (	Yes	No	No	No	No	No
moderate	adequate (adequate (	No	No	Yes	Yes	No	No
moderate	adequate (adequate (	No	No	No	Yes	No	No
		No	No	Yes	Yes	No	No
deficiency	adequate or moderat	No	No	Yes	No	No	No
iciency	Marked or severate	No	No	Yes	No	No	No
moderate	adequate (adequate (	No	No	Yes	Yes	No	No
		No	No	No	No	No	No
moderate	Marked or adequate (	No	No	No	No	No	No
adequate	adequate (adequate (	No	No	Yes	Yes	No	No
		No	No	No	No	No	No
moderate	adequate (adequate (	No	No	No	No	No	No
		No	No	No	Yes	No	No
adequate	adequate (adequate (	No	No	No	Yes	No	No
severe def	Marked or Marked or	No	No	No	Yes	No	No
adequate	adequate (adequate (	No	No	No	No	Yes	No
moderate	Marked or adequate (	No	No	No	No	No	No
adequate	adequate (adequate (	No	Yes	No	No	No	No
		No	No	No	Yes	No	Yes
		No	No	No	Yes	No	No

moderate	Marked or adequate (		No	No	No	No	No	No
			Yes	No	No	No	No	No
			Yes	No	Yes	No	No	Yes
			Yes	Yes	No	No	Yes	Yes
			Yes	Yes	No	No	No	No
			Yes	No	No	No	Yes	No
			Yes	No	No	No	Yes	Yes
			Yes	No	No	Yes	No	No
			Yes	No	Yes	No	No	No
			No	No	No	No	No	No
			Yes	No	Yes	No	No	No
			Yes	Yes	No	Yes	No	No
			No	No	Yes	No	No	No
			No	No	No	No	No	No
			No	No	No	No	No	Yes
moderate	adequate (	adequate (	No	No	Yes	No	No	No
			No	No	Yes	Yes	No	No
			No	No	No	Yes	No	No
			No	Yes	No	No	No	No
moderate	adequate (	adequate (	No	No	No	Yes	No	No
			No	No	Yes	Yes	No	Yes
adequate	adequate (	adequate (	Yes	Yes	No	Yes	No	No
			No	No	No	No	No	No
			Yes	No	No	Yes	No	No
moderate	Marked or adequate (		No	No	No	No	No	No
adequate	adequate (	adequate (	Yes	Yes	Yes	Yes	No	No
			No	No	No	No	No	No
			No	Yes	Yes	Yes	No	No
			No	No	Yes	Yes	No	No
adequate	Marked or adequate (		No	Yes	No	Yes	No	No
adequate	adequate (	adequate (	Yes	No	No	Yes	No	No
moderate	adequate (	adequate (	No	No	Yes	No	No	No
adequate	adequate (	adequate (	No	No	No	No	No	No
			No	No	No	No	No	No
moderate	Marked or adequate (		Yes	No	No	No	No	No
			No	Yes	Yes	No	No	No
adequate	Marked or adequate (		Yes	No	No	No	No	No
adequate	adequate (	adequate (	Yes	No	No	Yes	No	No
marked de	Marked or	Marked or	Yes	Yes	No	No	No	No
			Yes	No	No	No	No	No
moderate	adequate (	adequate (	No	No	No	No	No	No
adequate	adequate (	adequate (	Yes	No	Yes	No	No	No
			Yes	No	Yes	No	No	No
moderate	Marked or adequate (		Yes	Yes	No	No	Yes	Yes
moderate	adequate (	adequate (	Yes	Yes	No	No	No	No
			No	No	Yes	No	No	No
adequate	adequate (	adequate (	Yes	Yes	No	No	No	No
adequate	adequate (	adequate (	Yes	Yes	No	No	No	No
adequate	adequate (	adequate (	Yes	No	No	No	No	No
adequate	adequate (	adequate (	Yes	No	No	No	No	No
moderate	Marked or adequate (		Yes	No	No	No	No	No

			Yes	No	No	No	No	Yes
moderate	Marked or adequate (		No	Yes	No	No	No	No
			Yes	Yes	No	Yes	Yes	No
			Yes	Yes	No	No	No	Yes
			No	Yes	No	No	No	No
			Yes	Yes	No	No	No	No
			No	No	No	No	No	No
			No	No	No	No	No	No
marked de	Marked or	Marked or	Yes	No	Yes	No	No	No
moderate	adequate (	adequate (	No	No	Yes	No	No	No
			No	No	No	No	No	No
			No	Yes	Yes	No	No	No
			Yes	No	No	No	No	No
adequate	adequate (	adequate (	Yes	No	No	No	No	No
			Yes	No	No	Yes	No	No
			No	No	No	No	No	No
			No	Yes	No	Yes	No	No
moderate	adequate (	adequate (	No	No	No	No	No	No
deficiency	adequate or moderat		Yes	No	No	No	No	No
moderate	Marked or adequate (		Yes	No	No	No	No	No
			No	No	Yes	No	No	No
moderate	Marked or adequate (		No	No	No	No	No	No
moderate	adequate (	adequate (	Yes	Yes	No	No	No	No
marked de	Marked or	Marked or	Yes	No	No	No	No	No
adequate	adequate (	adequate (	Yes	No	No	Yes	No	No
moderate	adequate (	adequate (	No	No	No	Yes	No	No
adequate	adequate (	adequate (	Yes	No	Yes	No	No	No
			No	No	No	Yes	No	No
moderate	adequate (	adequate (	No	No	No	No	No	No
moderate	adequate (	adequate (	No	No	Yes	No	No	No
			No	No	No	No	No	No
adequate	adequate (	adequate (	No	No	Yes	No	No	No
adequate	adequate (	adequate (	No	No	No	No	No	No
adequate	adequate (	adequate (	No	No	No	No	No	No
			No	No	Yes	No	No	No
moderate	adequate (	adequate (	No	No	Yes	No	Yes	Yes
adequate	adequate (	adequate (	No	No	Yes	No	No	No

diar_exit	fever_exit	los	los_cat
No	Yes	21	los<8 weeks
No	No	70	los>=8 weeks
No	Yes	112	los>=8 weeks
No	No	112	los>=8 weeks
Yes	No	112	los>=8 weeks
No	No	91	los>=8 weeks
No	No	49	los<8 weeks
No	No	112	los>=8 weeks
Yes	No	7	
No	No	112	los>=8 weeks
No	No	77	los>=8 weeks
No	No	84	los>=8 weeks
No	No	112	los>=8 weeks
No	No	28	los<8 weeks
No	No	63	los>=8 weeks
No	No	28	los<8 weeks
No	No	28	los<8 weeks
No	Yes	49	los<8 weeks
No	No	42	los<8 weeks
No	No	28	los<8 weeks
No	No	42	los<8 weeks
No	No	49	los<8 weeks
No	No	49	los<8 weeks
No	No	70	los>=8 weeks
No	No	7	los<8 weeks
No	No	63	los>=8 weeks
No	No	98	los>=8 weeks
No	No	28	los<8 weeks
No	No	49	los<8 weeks
No	Yes	112	los>=8 weeks
No	No	28	los<8 weeks
No	No	105	los>=8 weeks
No	No	56	los>=8 weeks
No	No	70	los>=8 weeks
No	No	70	los>=8 weeks
No	No	21	los<8 weeks
No	No	49	los<8 weeks
No	No	84	los>=8 weeks
No	No	63	los>=8 weeks
No	No	112	los>=8 weeks
No	No	21	los<8 weeks
No	No	35	los<8 weeks
No	No	84	los>=8 weeks
No	No	7	los<8 weeks
No	No	63	los>=8 weeks
No	No	21	los<8 weeks
Yes	No	112	los>=8 weeks
No	No	63	los>=8 weeks
No	No	112	los>=8 weeks
No	No	28	los<8 weeks

No	No	35	los<8 weeks
No	No	42	los<8 weeks
No	No	35	los<8 weeks
No	No	56	los>=8 weeks
No	No	77	los>=8 weeks
No	No	112	los>=8 weeks
No	No	42	los<8 weeks
No	Yes	14	los<8 weeks
Yes	No	7	los<8 weeks
No	No	70	los>=8 weeks
No	No	112	los>=8 weeks
No	No	112	los>=8 weeks
No	No	112	los>=8 weeks
No	No	14	los<8 weeks
No	No	63	los>=8 weeks
No	No	91	los>=8 weeks
No	No	35	los<8 weeks
No	No	70	los>=8 weeks
No	No	28	los<8 weeks
No	No	28	los<8 weeks
No	No	91	los>=8 weeks
No	No	56	los>=8 weeks
No	No	49	los<8 weeks
No	No	21	los<8 weeks
Yes	No	112	los>=8 weeks
No	No	105	los>=8 weeks
No	No	112	los>=8 weeks
No	No	63	los>=8 weeks
No	No	49	los<8 weeks
No	No	42	los<8 weeks
No	No	0	
No	No	112	los>=8 weeks
No	No	42	los<8 weeks
No	Yes	84	los>=8 weeks
No	No	70	los>=8 weeks
No	No	112	los>=8 weeks
No	No	56	los>=8 weeks
No	No	56	los>=8 weeks
No	No	56	los>=8 weeks
No	No	112	los>=8 weeks
No	No	7	los<8 weeks
No	No	28	los<8 weeks
No	No	35	los<8 weeks
Yes	No	0	
No	No	49	los<8 weeks
No	No	28	los<8 weeks
No	No	98	los>=8 weeks
No	No	70	los>=8 weeks
No	No	63	los>=8 weeks
No	No	70	los>=8 weeks
No	No	77	los>=8 weeks

No	No	56	los>=8 weeks
No	No	35	los<8 weeks
No	No	35	los<8 weeks
No	No	28	los<8 weeks
No	No	28	los<8 weeks
No	No	70	los>=8 weeks
No	No	112	los>=8 weeks
No	No	91	los>=8 weeks
No	No	77	los>=8 weeks
No	No	63	los>=8 weeks
No	No	42	los<8 weeks
No	No	112	los>=8 weeks
No	No	42	los<8 weeks
No	Yes	56	los>=8 weeks
No	No	49	los<8 weeks
No	No	98	los>=8 weeks
No	No	112	los>=8 weeks
No	No	112	los>=8 weeks
No	No	77	los>=8 weeks
No	No	84	los>=8 weeks
No	No	28	los<8 weeks
No	No	35	los<8 weeks
No	No	77	los>=8 weeks
No	No	56	los>=8 weeks
No	No	63	los>=8 weeks
No	No	105	los>=8 weeks
No	No	21	los<8 weeks
Yes	Yes	28	los<8 weeks
No	No	56	los>=8 weeks
No	No	91	los>=8 weeks
No	No	91	los>=8 weeks
No	No	112	los>=8 weeks
No	No	77	los>=8 weeks
No	No	28	los<8 weeks
No	No	70	los>=8 weeks
No	No	70	los>=8 weeks
No	No	49	los<8 weeks
No	No	105	los>=8 weeks
No	No	21	los<8 weeks
No	No	28	los<8 weeks
No	No	112	los>=8 weeks
No	No	56	los>=8 weeks
No	No	112	los>=8 weeks
No	No	28	los<8 weeks
No	No	28	los<8 weeks
No	No	28	los<8 weeks
No	No	14	los<8 weeks
No	No	91	los>=8 weeks
No	No	56	los>=8 weeks
No	No	105	los>=8 weeks
No	No	21	los<8 weeks

No	No	49	los<8 weeks
No	No	112	los>=8 weeks
No	Yes	42	los<8 weeks
Yes	No	112	los>=8 weeks
No	No	112	los>=8 weeks
No	No	21	los<8 weeks
No	No	42	los<8 weeks
No	No	56	los>=8 weeks
No	No	21	los<8 weeks
No	Yes	56	los>=8 weeks
No	No	21	los<8 weeks
No	Yes	49	los<8 weeks
No	Yes	35	los<8 weeks
No	No	35	los<8 weeks
No	No	35	los<8 weeks
No	No	28	los<8 weeks
No	No	91	los>=8 weeks
No	No	28	los<8 weeks
No	No	35	los<8 weeks
No	No	112	los>=8 weeks
No	No	35	los<8 weeks
No	No	42	los<8 weeks
No	No	56	los>=8 weeks
No	No	105	los>=8 weeks
No	No	112	los>=8 weeks
No	No	84	los>=8 weeks
No	No	42	los<8 weeks
No	No	35	los<8 weeks
No	No	49	los<8 weeks
Yes	No	112	los>=8 weeks
No	No	84	los>=8 weeks
No	No	35	los<8 weeks
No	No	84	los>=8 weeks
No	No	35	los<8 weeks
Yes	No	112	los>=8 weeks
No	No	119	los>=8 weeks
No	No	42	los<8 weeks
No	No	70	los>=8 weeks
No	No	49	los<8 weeks
No	No	35	los<8 weeks
No	No	105	los>=8 weeks
No	No	98	los>=8 weeks
No	No	42	los<8 weeks
No	No	56	los>=8 weeks
No	No	84	los>=8 weeks
No	No	42	los<8 weeks
No	No	112	los>=8 weeks
No	No	7	los<8 weeks
No	No	14	los<8 weeks
No	No	112	los>=8 weeks
No	No	49	los<8 weeks

No	No	42	los<8 weeks
No	No	49	los<8 weeks
No	No	98	los>=8 weeks
No	No	14	los<8 weeks
No	No	70	los>=8 weeks
No	No	42	los<8 weeks
No	No	98	los>=8 weeks
No	No	21	los<8 weeks
Yes	Yes	70	los>=8 weeks
Yes	No	112	los>=8 weeks
No	Yes	98	los>=8 weeks
Yes	Yes	93	los>=8 weeks
No	No	56	los>=8 weeks
Yes	Yes	7	los<8 weeks
No	No	84	los>=8 weeks
No	No	42	los<8 weeks
No	No	28	los<8 weeks
No	No	63	los>=8 weeks
Yes	No	70	los>=8 weeks
No	No	49	los<8 weeks
Yes	Yes	35	los<8 weeks
No	No	35	los<8 weeks
No	No	70	los>=8 weeks
No	No	28	los<8 weeks
No	No	70	los>=8 weeks
No	No	112	los>=8 weeks
No	No	63	los>=8 weeks
No	No	84	los>=8 weeks
No	No	42	los<8 weeks
No	No	70	los>=8 weeks
No	No	14	los<8 weeks
No	No	98	los>=8 weeks
No	No	77	los>=8 weeks
No	No	42	los<8 weeks
No	No	56	los>=8 weeks
No	No	0	
No	No	42	los<8 weeks
No	No	105	los>=8 weeks
No	No	84	los>=8 weeks
No	No	112	los>=8 weeks
No	No	35	los<8 weeks
No	No	70	los>=8 weeks
No	No	42	los<8 weeks
No	No	7	los<8 weeks
No	No	49	los<8 weeks
No	Yes	0	
No	No	77	los>=8 weeks
No	No	42	los<8 weeks
No	No	28	los<8 weeks
No	No	42	los<8 weeks
No	No	84	los>=8 weeks

No	No	49	los<8 weeks
No	No	84	los>=8 weeks
No	No	105	los>=8 weeks
No	No	105	los>=8 weeks
No	No	41	los<8 weeks
No	No	35	los<8 weeks
No	No	21	los<8 weeks
No	No	21	los<8 weeks
No	No	28	los<8 weeks
No	No	42	los<8 weeks
No	No	112	los>=8 weeks
No	No	35	los<8 weeks
No	No	112	los>=8 weeks
No	No	42	los<8 weeks
No	No	35	los<8 weeks
No	No	56	los>=8 weeks
No	No	70	los>=8 weeks
No	No	49	los<8 weeks
No	No	21	los<8 weeks
No	No	56	los>=8 weeks
No	No	77	los>=8 weeks
No	No	56	los>=8 weeks
No	Yes	14	los<8 weeks
No	No	21	los<8 weeks
No	No	63	los>=8 weeks
No	No	112	los>=8 weeks
No	No	28	los<8 weeks
No	No	63	los>=8 weeks
No	Yes	35	los<8 weeks
No	No	42	los<8 weeks
No	No	70	los>=8 weeks
No	No	35	los<8 weeks
No	No	28	los<8 weeks
No	No	49	los<8 weeks
No	No	105	los>=8 weeks
No	No	49	los<8 weeks
No	No	35	los<8 weeks
No	No	35	los<8 weeks
No	No	105	los>=8 weeks
No	No	112	los>=8 weeks
No	Yes	21	los<8 weeks
No	No	35	los<8 weeks
No	No	105	los>=8 weeks
No	No	98	los>=8 weeks
No	No	98	los>=8 weeks
No	No	28	los<8 weeks
No	No	112	los>=8 weeks
No	No	112	los>=8 weeks
Yes	Yes	7	los<8 weeks
No	No	28	los<8 weeks
No	No	112	los>=8 weeks

No	No	49	los<8 weeks
No	No	84	los>=8 weeks
No	No	42	los<8 weeks
No	No	42	los<8 weeks
No	No	112	los>=8 weeks
No	No	21	los<8 weeks
No	No	70	los>=8 weeks
No	No	70	los>=8 weeks
No	No	28	los<8 weeks
No	No	56	los>=8 weeks
No	No	91	los>=8 weeks
No	No	56	los>=8 weeks
No	No	21	los<8 weeks
No	No	35	los<8 weeks
No	No	105	los>=8 weeks
No	No	56	los>=8 weeks
No	No	28	los<8 weeks
No	No	28	los<8 weeks
No	No	42	los<8 weeks
No	Yes	7	
No	No	112	los>=8 weeks
No	No	70	los>=8 weeks
No	No	63	los>=8 weeks
No	No	49	los<8 weeks
No	No	35	los<8 weeks
No	No	14	los<8 weeks
No	No	70	los>=8 weeks
No	No	112	los>=8 weeks
No	No	84	los>=8 weeks
No	No	77	los>=8 weeks
No	No	105	los>=8 weeks
No	No	112	los>=8 weeks
No	No	105	los>=8 weeks
No	No	21	los<8 weeks
No	No	28	los<8 weeks
No	No	42	los<8 weeks
No	No	21	los<8 weeks
No	No	21	los<8 weeks
No	No	105	los>=8 weeks
No	No	63	los>=8 weeks
No	No	112	los>=8 weeks
No	No	42	los<8 weeks
No	No	28	los<8 weeks
No	No	77	los>=8 weeks
No	No	77	los>=8 weeks
No	No	21	los<8 weeks
No	No	49	los<8 weeks
No	No	105	los>=8 weeks
No	No	56	los>=8 weeks
No	No	35	los<8 weeks
No	No	21	los<8 weeks

No	No	42	los<8 weeks
No	No	21	los<8 weeks
No	No	28	los<8 weeks
No	No	28	los<8 weeks
No	No	105	los>=8 weeks
No	No	28	los<8 weeks
No	No	56	los>=8 weeks
No	No	7	los<8 weeks
No	No	105	los>=8 weeks
Yes	No	35	los<8 weeks
No	No	28	los<8 weeks
No	No	112	los>=8 weeks
Yes	Yes	49	los<8 weeks
No	No	42	los<8 weeks
No	No	112	los>=8 weeks
No	No	77	los>=8 weeks
No	No	70	los>=8 weeks
No	No	112	los>=8 weeks
No	No	98	los>=8 weeks
No	No	77	los>=8 weeks
No	No	42	los<8 weeks
No	No	28	los<8 weeks
No	No	42	los<8 weeks
No	No	105	los>=8 weeks
No	No	21	los<8 weeks
No	No	21	los<8 weeks
No	No	49	los<8 weeks
No	No	112	los>=8 weeks
No	No	112	los>=8 weeks
No	No	35	los<8 weeks
No	No	21	los<8 weeks
No	No	70	los>=8 weeks
No	No	35	los<8 weeks
No	No	49	los<8 weeks
No	No	77	los>=8 weeks
No	No	105	los>=8 weeks
No	No	91	los>=8 weeks
No	No	112	los>=8 weeks
No	No	49	los<8 weeks
No	Yes	112	los>=8 weeks
No	Yes	112	los>=8 weeks
No	No	14	los<8 weeks
No	No	77	los>=8 weeks
No	No	14	los<8 weeks
No	No	28	los<8 weeks
No	No	49	los<8 weeks
No	No	98	los>=8 weeks
No	No	21	los<8 weeks
No	No	42	los<8 weeks
Yes	No	112	los>=8 weeks
No	No	7	los<8 weeks

No	Yes	28	los<8 weeks
No	No	28	los<8 weeks
No	No	98	los>=8 weeks
No	No	84	los>=8 weeks
No	No	35	los<8 weeks
No	No	35	los<8 weeks
No	Yes	105	los>=8 weeks
No	No	28	los<8 weeks
No	No	70	los>=8 weeks
No	No	112	los>=8 weeks
No	No	70	los>=8 weeks
No	No	119	los>=8 weeks
No	No	63	los>=8 weeks
No	No	0	
No	No	21	los<8 weeks
Yes	Yes	77	los>=8 weeks
No	No	84	los>=8 weeks
No	No	7	los<8 weeks
No	No	91	los>=8 weeks
No	No	28	los<8 weeks
No	No	84	los>=8 weeks
No	No	49	los<8 weeks
No	No	112	los>=8 weeks
No	No	112	los>=8 weeks
No	No	77	los>=8 weeks
No	No	105	los>=8 weeks
No	No	112	los>=8 weeks
No	No	42	los<8 weeks
No	No	49	los<8 weeks
No	No	35	los<8 weeks
No	No	14	los<8 weeks
No	No	35	los<8 weeks
No	No	42	los<8 weeks
No	No	21	los<8 weeks
No	No	42	los<8 weeks
No	No	35	los<8 weeks
No	No	14	los<8 weeks
No	No	112	los>=8 weeks
No	No	112	los>=8 weeks
No	No	98	los>=8 weeks
No	No	56	los>=8 weeks
No	No	84	los>=8 weeks
No	No	112	los>=8 weeks
No	No	91	los>=8 weeks
No	No	112	los>=8 weeks
Yes	Yes	7	los<8 weeks
No	No	7	los<8 weeks
No	No	105	los>=8 weeks
Yes	No	28	los<8 weeks
No	No	49	los<8 weeks
No	Yes	56	los>=8 weeks

No	No	49	los<8 weeks
No	No	77	los>=8 weeks
No	No	84	los>=8 weeks
No	No	112	los>=8 weeks
No	No	91	los>=8 weeks
No	No	56	los>=8 weeks
No	No	42	los<8 weeks
No	No	35	los<8 weeks
No	No	49	los<8 weeks
No	No	42	los<8 weeks
No	No	35	los<8 weeks
No	No	112	los>=8 weeks
Yes	Yes	35	los<8 weeks
No	No	91	los>=8 weeks
No	No	63	los>=8 weeks
No	No	77	los>=8 weeks
No	No	91	los>=8 weeks
No	No	49	los<8 weeks
No	No	105	los>=8 weeks
No	No	112	los>=8 weeks
No	No	56	los>=8 weeks
No	No	49	los<8 weeks
No	No	42	los<8 weeks
No	No	28	los<8 weeks
No	No	91	los>=8 weeks
No	No	112	los>=8 weeks
No	No	112	los>=8 weeks
No	No	21	los<8 weeks
No	No	70	los>=8 weeks
No	No	56	los>=8 weeks
No	No	35	los<8 weeks
No	No	84	los>=8 weeks
No	No	42	los<8 weeks
No	No	0	
No	No	105	los>=8 weeks
No	No	21	los<8 weeks
No	No	56	los>=8 weeks
No	No	42	los<8 weeks
No	No	35	los<8 weeks
No	No	42	los<8 weeks
No	No	28	los<8 weeks
No	No	112	los>=8 weeks
No	No	42	los<8 weeks
No	No	35	los<8 weeks
No	No	21	los<8 weeks
No	No	105	los>=8 weeks
No	No	105	los>=8 weeks
No	No	91	los>=8 weeks
No	No	21	los<8 weeks
No	No	77	los>=8 weeks
No	No	56	los>=8 weeks

No	No	42	los<8 weeks
No	No	28	los<8 weeks
No	No	49	los<8 weeks
No	Yes	7	los<8 weeks
No	No	112	los>=8 weeks
No	No	105	los>=8 weeks
No	No	77	los>=8 weeks
No	No	49	los<8 weeks
No	No	7	los<8 weeks
No	No	7	los<8 weeks
No	No	63	los>=8 weeks
No	No	91	los>=8 weeks
No	No	91	los>=8 weeks
No	No	112	los>=8 weeks
No	No	35	los<8 weeks
No	No	57	los>=8 weeks
No	No	77	los>=8 weeks
No	No	35	los<8 weeks
No	No	63	los>=8 weeks
No	No	49	los<8 weeks
Yes	No	28	los<8 weeks
No	No	35	los<8 weeks
Yes	No	35	los<8 weeks
No	No	105	los>=8 weeks
No	No	105	los>=8 weeks
No	No	91	los>=8 weeks
No	No	35	los<8 weeks
Yes	No	112	los>=8 weeks
No	No	91	los>=8 weeks
No	No	70	los>=8 weeks
No	No	98	los>=8 weeks
No	Yes	21	los<8 weeks
Yes	No	105	los>=8 weeks
No	No	42	los<8 weeks
No	No	49	los<8 weeks
No	No	112	los>=8 weeks
No	No	56	los>=8 weeks
No	No	105	los>=8 weeks
No	No	42	los<8 weeks
Yes	No	112	los>=8 weeks
No	Yes	49	los<8 weeks
No	No	28	los<8 weeks
No	No	112	los>=8 weeks
No	No	112	los>=8 weeks
No	No	21	los<8 weeks
No	No	84	los>=8 weeks
No	No	112	los>=8 weeks
No	No	35	los<8 weeks
No	No	105	los>=8 weeks
No	No	112	los>=8 weeks
No	No	70	los>=8 weeks

No	No	112	los>=8 weeks
No	No	21	los<8 weeks
No	No	56	los>=8 weeks
No	Yes	42	los<8 weeks
No	No	56	los>=8 weeks
No	No	91	los>=8 weeks
No	No	56	los>=8 weeks
No	No	77	los>=8 weeks
No	No	35	los<8 weeks
No	No	42	los<8 weeks
No	No	56	los>=8 weeks
No	No	28	los<8 weeks
No	No	112	los>=8 weeks
No	No	35	los<8 weeks
Yes	No	28	los<8 weeks
No	No	70	los>=8 weeks
No	No	49	los<8 weeks
No	No	56	los>=8 weeks
No	No	0	
Yes	No	0	
No	No	84	los>=8 weeks
No	Yes	70	los>=8 weeks
Yes	No	49	los<8 weeks
No	No	49	los<8 weeks
No	Yes	105	los>=8 weeks
No	No	56	los>=8 weeks
No	No	63	los>=8 weeks
No	Yes	49	los<8 weeks
No	No	84	los>=8 weeks
No	No	49	los<8 weeks
No	No	42	los<8 weeks
No	No	35	los<8 weeks
No	No	42	los<8 weeks
No	No	28	los<8 weeks
No	No	70	los>=8 weeks
No	Yes	21	los<8 weeks
No	No	42	los<8 weeks
No	No	84	los>=8 weeks
No	No	14	los<8 weeks
No	No	35	los<8 weeks
No	Yes	21	los<8 weeks
No	No	28	los<8 weeks
No	No	28	los<8 weeks
No	No	49	los<8 weeks
No	No	112	los>=8 weeks
No	No	35	los<8 weeks
No	No	70	los>=8 weeks
Yes	No	63	los>=8 weeks
No	No	112	los>=8 weeks
No	No	112	los>=8 weeks
No	No	35	los<8 weeks

No	No	14	los<8 weeks
No	No	21	los<8 weeks
No	No	14	los<8 weeks
No	No	105	los>=8 weeks
No	No	28	los<8 weeks
No	No	35	los<8 weeks
Yes	No	112	los>=8 weeks
No	No	84	los>=8 weeks
No	No	56	los>=8 weeks
No	No	112	los>=8 weeks
No	No	49	los<8 weeks
No	No	70	los>=8 weeks
No	No	105	los>=8 weeks
No	No	84	los>=8 weeks
No	No	56	los>=8 weeks
No	No	56	los>=8 weeks
No	Yes	14	los<8 weeks
No	No	21	los<8 weeks
No	No	21	los<8 weeks
No	No	49	los<8 weeks
No	No	49	los<8 weeks
No	No	35	los<8 weeks
No	No	49	los<8 weeks
No	No	49	los<8 weeks
No	No	49	los<8 weeks
No	No	49	los<8 weeks
No	No	112	los>=8 weeks
No	No	42	los<8 weeks
No	No	63	los>=8 weeks
No	No	105	los>=8 weeks
No	No	42	los<8 weeks
No	No	21	los<8 weeks
No	No	28	los<8 weeks
No	No	98	los>=8 weeks
No	No	70	los>=8 weeks
No	Yes	63	los>=8 weeks
No	No	98	los>=8 weeks
No	No	42	los<8 weeks
No	No	84	los>=8 weeks
No	No	84	los>=8 weeks
Yes	No	49	los<8 weeks
No	No	105	los>=8 weeks
No	No	42	los<8 weeks
No	No	42	los<8 weeks
No	No	42	los<8 weeks
No	No	49	los<8 weeks
No	No	91	los>=8 weeks
No	No	98	los>=8 weeks
No	Yes	70	los>=8 weeks
No	No	112	los>=8 weeks
No	No	49	los<8 weeks

Yes	No	35	los<8 weeks
No	No	14	los<8 weeks
No	No	77	los>=8 weeks
No	No	42	los<8 weeks
Yes	No	35	los<8 weeks
No	No	21	los<8 weeks
No	No	21	los<8 weeks
No	No	70	los>=8 weeks
No	No	70	los>=8 weeks
Yes	No	42	los<8 weeks
No	No	49	los<8 weeks
No	No	112	los>=8 weeks
No	No	14	los<8 weeks
No	No	35	los<8 weeks
No	Yes	112	los>=8 weeks
No	Yes	42	los<8 weeks
No	No	28	los<8 weeks
No	Yes	112	los>=8 weeks
No	No	42	los<8 weeks
No	No	21	los<8 weeks
No	No	28	los<8 weeks
No	No	28	los<8 weeks
No	No	28	los<8 weeks
No	No	77	los>=8 weeks
No	No	105	los>=8 weeks
No	Yes	35	los<8 weeks
No	No	28	los<8 weeks
No	No	21	los<8 weeks
No	No	49	los<8 weeks
No	No	28	los<8 weeks
No	No	56	los>=8 weeks
No	No	70	los>=8 weeks
No	No	35	los<8 weeks
No	No	84	los>=8 weeks
No	No	77	los>=8 weeks
No	No	77	los>=8 weeks
No	Yes	28	los<8 weeks
No	No	35	los<8 weeks
No	No	42	los<8 weeks
No	No	42	los<8 weeks
No	No	42	los<8 weeks
No	No	35	los<8 weeks
No	No	63	los>=8 weeks
No	No	91	los>=8 weeks
No	No	56	los>=8 weeks
No	No	35	los<8 weeks
Yes	No	63	los>=8 weeks
No	No	35	los<8 weeks
No	No	42	los<8 weeks
No	Yes	105	los>=8 weeks
No	No	112	los>=8 weeks

No	No	49	los<8 weeks
No	No	70	los>=8 weeks
No	Yes	112	los>=8 weeks
No	No	0	
No	No	70	los>=8 weeks
No	No	0	
Yes	No	112	los>=8 weeks
No	No	63	los>=8 weeks
No	No	57	los>=8 weeks
Yes	No	49	los<8 weeks
No	No	56	los>=8 weeks
No	No	112	los>=8 weeks
No	No	70	los>=8 weeks
No	Yes	42	los<8 weeks
No	No	63	los>=8 weeks
No	No	91	los>=8 weeks
No	No	35	los<8 weeks
No	No	98	los>=8 weeks
Yes	No	70	los>=8 weeks
No	No	70	los>=8 weeks
Yes	Yes	14	los<8 weeks
No	No	77	los>=8 weeks
No	No	112	los>=8 weeks
No	No	35	los<8 weeks
No	No	28	los<8 weeks
No	No	42	los<8 weeks
No	Yes	7	los<8 weeks
No	No	14	los<8 weeks
No	No	21	los<8 weeks
No	No	91	los>=8 weeks
No	No	70	los>=8 weeks
No	No	21	los<8 weeks
No	No	28	los<8 weeks
No	Yes	112	los>=8 weeks
No	No	56	los>=8 weeks
No	No	63	los>=8 weeks
No	Yes	112	los>=8 weeks
No	No	98	los>=8 weeks
No	No	42	los<8 weeks
No	No	84	los>=8 weeks
Yes	No	49	los<8 weeks
No	No	42	los<8 weeks
No	No	14	los<8 weeks
No	Yes	42	los<8 weeks
No	No	56	los>=8 weeks
No	No	0	
No	No	49	los<8 weeks
No	No	49	los<8 weeks
No	No	56	los>=8 weeks
No	No	56	los>=8 weeks
No	No	56	los>=8 weeks

No	No	28	los<8 weeks
No	No	63	los>=8 weeks
No	No	112	los>=8 weeks
No	No	42	los<8 weeks
No	No	84	los>=8 weeks
No	No	28	los<8 weeks
No	No	112	los>=8 weeks
No	No	28	los<8 weeks
No	No	84	los>=8 weeks
No	No	63	los>=8 weeks
No	No	77	los>=8 weeks
No	No	112	los>=8 weeks
No	No	91	los>=8 weeks
No	No	112	los>=8 weeks
No	No	98	los>=8 weeks
No	No	91	los>=8 weeks
No	Yes	70	los>=8 weeks
No	No	112	los>=8 weeks
No	No	84	los>=8 weeks
No	No	28	los<8 weeks
No	No	84	los>=8 weeks
No	No	70	los>=8 weeks
No	No	105	los>=8 weeks
No	No	63	los>=8 weeks
No	No	70	los>=8 weeks
No	No	63	los>=8 weeks
No	No	56	los>=8 weeks
No	No	56	los>=8 weeks
No	No	84	los>=8 weeks
No	Yes	34	los<8 weeks
No	No	98	los>=8 weeks
No	No	105	los>=8 weeks
No	No	84	los>=8 weeks
No	No	21	los<8 weeks
No	No	49	los<8 weeks
No	No	112	los>=8 weeks
No	No	70	los>=8 weeks
